# Polar functional group-containing glycolipid CD1d ligands modulate cytokine-biasing responses and prevent experimental colitis

**DOI:** 10.1038/s41598-020-72280-4

**Published:** 2020-09-25

**Authors:** Shinsuke Inuki, Natsumi Hirata, Emi Kashiwabara, Junichiro Kishi, Toshihiko Aiba, Toshiaki Teratani, Wataru Nakamura, Yoshimi Kojima, Toru Maruyama, Takanori Kanai, Yukari Fujimoto

**Affiliations:** 1 Graduate School of Science and Technology, Keio University, Hiyoshi, Kohoku-ku, Yokohama, Kanagawa 223-8522 Japan; 2 Graduate School of Pharmaceutical Sciences, Kyoto University, Sakyo-ku, Kyoto, 606-8501 Japan; 3 Department of Chemistry, Graduate School of Science, Osaka University, Machikaneyama-cho, Toyonaka, Osaka 560-0043 Japan; 4 School of Medicine, Keio University, Shinanomachi, Shinjuku-ku, Tokyo, 160-8582 Japan; 5 Discovery and Research, ONO Pharmaceutical Co., Ltd., Sakurai, Shimamoto, Mishima, Osaka 618-8585 Japan

**Keywords:** Carbohydrates, Drug development

## Abstract

The MHC class I-like molecule CD1d is a nonpolymorphic antigen-presenting glycoprotein, and its ligands include glycolipids, such as α-GalCer. The complexes between CD1d and ligands activate natural killer T cells by T cell receptor recognition, leading to the secretion of various cytokines (IFN-γ, IL-4, IL-17A, etc.). Herein, we report structure–activity relationship studies of α-GalCer derivatives containing various functional groups in their lipid acyl chains. Several derivatives have been identified as potent CD1d ligands displaying higher cytokine induction levels and/or unique cytokine polarization. The studies also indicated that flexibility of the lipid moiety can affect the binding affinity, the total cytokine production level and/or cytokine biasing. Based on our immunological evaluation and investigation of physicochemical properties, we chose bisamide- and Bz amide-containing derivatives **2** and **3**, and evaluated their in vivo efficacy in a DSS-induced model of ulcerative colitis. The derivative **3** that exhibits Th2- and Th17-biasing responses, demonstrated significant protective effects against intestinal inflammation in the DSS-induced model, after a single intraperitoneal injection.

## Introduction

Invariant natural killer T (iNKT) cells expressing an invariant α chain T cell receptor (TCR; Vα14–Jα18 in mice and Vα24-Jα18 in humans), play a critical role in regulating various immune responses^1,2^. iNKT cells recognize glycolipid ligands present in the nonpolymorphic MHC class I-like molecule CD1d protein by using TCRs, and then are activated. iNKT cell activation induces the secretion of various cytokines including Th1 (e.g. IFN-γ), Th2 (e.g. IL-4) or Th17 cytokines (e.g. IL-17A). Th1 cytokines are involved in the enhancement of inflammatory responses, and mainly induce cellular immune responses leading to antitumor^3,4^ or antiviral effects^5,6^. Thus, controlling Th1 responses has recently attracted significant attention as a therapeutic target for a range of cancers and viral infections. Th2 cytokines promote humoral immunity by participating in the antibody-mediated immunity control of extracellular pathogens. The production of Th2 cytokines is also associated with the amelioration of certain autoimmune diseases (e.g., IBD, type 1 diabetes and multiple sclerosis)^7–9^. Th17 cytokines can contribute to host defense against extracellular pathogens such as *Streptococcus pneumoniae*^10–13^. Thus, controlling Th17 cytokines can facilitate the development of vaccine adjuvants against pneumococcal infection. However, the details of the role Th17 cytokines in the CD1d-NKT system remain unclear. In addition to each characteristic, these cytokines can positively or negatively interact with each other, leading to the modulation of various immune responses. For instance, the Th1 and Th2 signaling pathways can negatively cross-regulate each other^14^. The Th1 and Th17 cytokines demonstrate synergistic behavior, leading to protection against extracellular pathogens, although in some cases, the disturbance of Th1 and Th17 can result in immunopathology^12^. The regulation of cytokine induction levels and/or balance is a key factor in the development of effective immunotherapies^15–17^. Therefore, multiple research groups have focused on the selective regulation of cytokine production^14^.

Importantly, previous structure–activity relationship studies (SAR) on CD1d ligands have shown that differences in the ligand structure can influence cytokine polarization from NKT cells^14^, although the underlying mechanism is not fully understood. Thus, to date, many researchers have actively explored and developed novel CD1d ligands on the basis of SAR studies of glycolipid ligands. Representative CD1d ligands, namely, α-galactosyl ceramide (α-GalCer, KRN7000)^18^, 7DW8-5^19^, OCH^20^ and a phytosphingosine-modified α-GalCer derivative^21^ are shown in Fig. S1. α-GalCer, one of the most potent ligands, induces high level production of various cytokines, and has had good therapeutic effects in some models, including cancer^18^ or autoimmune models^9^. 7DW8-5 displays Th1 polarization and has been identified as a lead candidate for HIV and malaria vaccine adjuvants^19^. OCH is an α-GalCer variant containing a truncated sphingosine moiety, and it shows IL-4 selectivity. OCH has been effective in autoimmune models such as a murine experimental autoimmune encephalitis (EAE) model^20^ or a murine dextran sulfate sodium (DSS) model of ulcerative colitis (UC)^22^. The phytosphingosine-modified α-GalCer derivative induced greater secretion of a Th2 cytokine, IL-4, over those of IFN-γ and IL-17, and was effective in the EAE model^21^.

In this context, we conducted SAR studies based on α-GalCer to obtain the potent and/or cytokine-biasing ligands^23–25^. In particular, we focused on a large hydrophobic pocket of CD1d (A’ pocket) that recognizes long fatty acyl chains of glycolipid ligands and developed CD1d ligands with polar functional groups such as amides or amines that display potent cytokine induction levels and Th2 cytokine polarization. Our molecular dynamics (MD) simulation studies predicted that amide groups on the ligands can interact with confined polar amino acid residues, Gln14 and Ser28 in the large hydrophobic pocket through direct and/or water-bridged hydrogen bonds^23^. However, the ligand-recognition mechanism of the lipid binding sites is still not understood, and the functional groups introduced onto the fatty acyl chains have not been fully optimized.

In this study, we conducted a detailed SAR study of α-GalCer derivatives containing functional groups in their long fatty acyl chains to obtain new potent ligands and elucidate the ligand-recognition mechanism of lipid binding. Additionally, their cytokine production selectivity (for IFN-γ, IL-4 and IL-17A) was investigated. We also evaluated their physicochemical and pharmacokinetic (PK) profiles and in vivo efficacy in a murine autoimmune model, a DSS model of inflammatory bowel diseases (IBD).

## Results and discussion

### Ligand design

**Figure 1:**
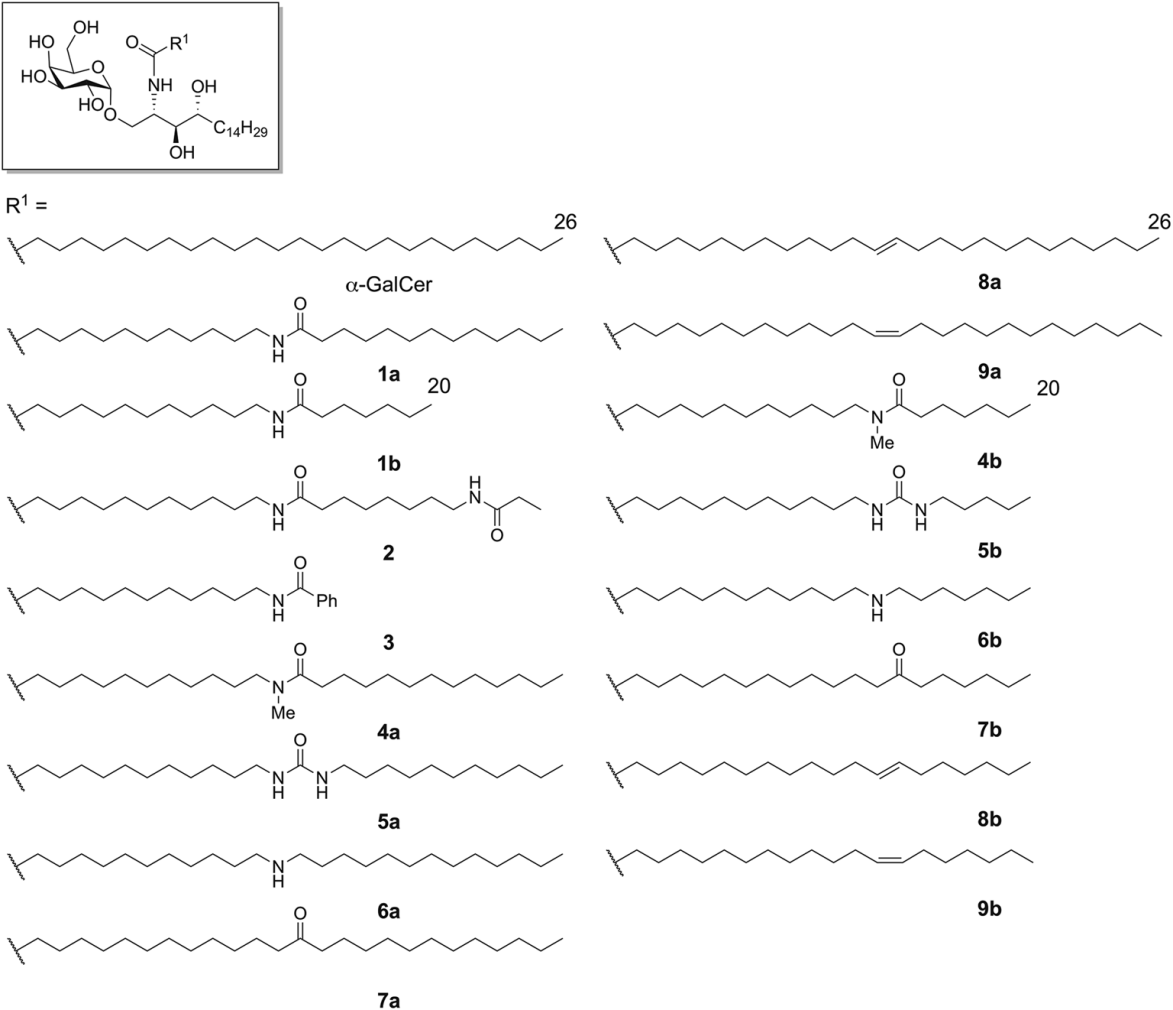
Structure of the **α**-GalCer derivatives containing polar and alkene groups.

We have previously reported several α-GalCer derivatives (**1**, **2**, **3**, and **6**) containing amide and amine groups in their lipid moieties, as shown in Fig. 1 (**1a**: C26:0 α-GalCer derivative having one amide-containing acyl chain, **1b**: C20:0 α-GalCer derivative having one amide-containing acyl chain, **2**: bisamide-containing α-GalCer derivative having two amide-containing acyl chain, **3**: Bz amide-containing α-GalCer derivative having one amide containing terminal Ar group, **6a**: C26:0 α-GalCer derivative having one amine-containing acyl chain, and **6b**: C20:0 α-GalCer derivative having one amine-containing acyl chain)^23,24^. The cytokine induction levels of all the ligands were higher or equal to that of C26:0 α-GalCer. Based on these findings, we designed CD1d ligands containing polar functional groups (**4a**: *N*-methyl amide, **5a**: urea and **7a**: carbonyl groups) to obtain highly potent and/or cytokine-biasing ligands and investigate the recognition of their polar residues by the A’ pocket in detail (Fig. 1). Furthermore, their corresponding C20:0 analogues (**4b**, **5b** and **7b**) were also prepared. Previous computational studies, including MD simulations, indicated that the formation of hydrogen bonds (shielded hydrogen bonds) between the amide group of the ligands and the polar residues in the hydrophobic A’ pocket of CD1d might enhance the recognition of the ligands by CD1d^23^. In contrast, the introduction of amide groups to saturated long acyl chains would partially rigidify the flexible lipid chains due to the planar structure of amide bonds, and this would mostly likely affect the interaction of these chains with CD1d. Thus, we also designed *trans*/*cis*-alkene-containing ligands **8** and **9** to elucidate the relationship between lipid conformation and CD1d affinity.

### Synthesis of the ligands

**Figure 2:**
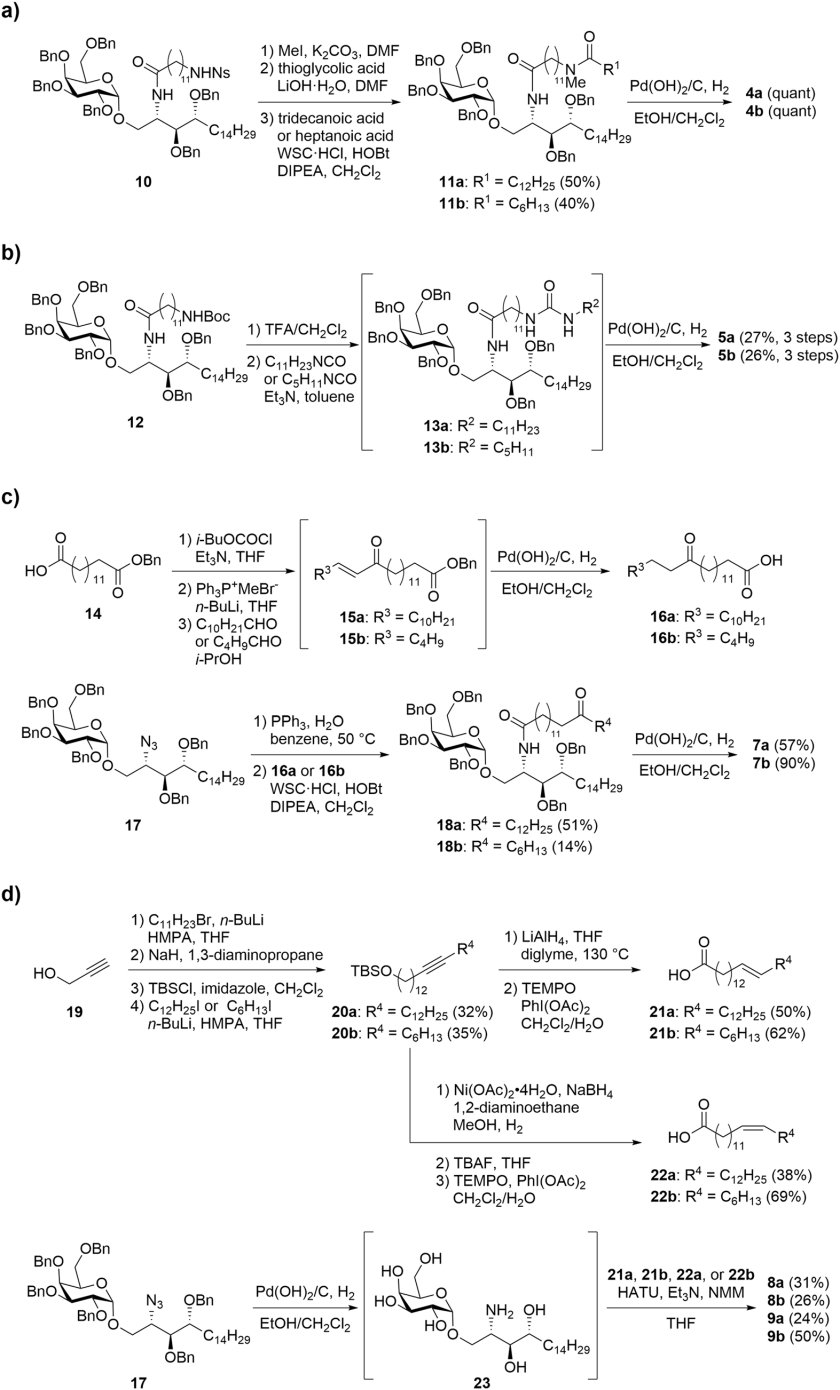
(**a**) Synthesis of α-GalCer derivatives **4a** and **4b**; (**b**) **5a** and **5b**; (**c**) **7a** and **7b**; (**d**) **8a**, **8b**, **9a** and **9b**.

The synthesis of the α-GalCer derivatives containing polar and alkene groups is shown in Fig. 2. First, we synthesized *N*-methyl amide derivatives **4a** and **4b** (Fig. 2a). The selective methylation of Ns amide derivative **10**^24^, removal of the Ns group and acylation with tridecanoic acid or heptanoic acid gave protected *N*-methyl amide derivatives **11a** and **11b**. Cleavage of the Bn groups with Pd(OH)_2_/C afforded desired *N*-methyl amide derivatives **4a** and **4b**. The preparation of urea analogues **5a** and **5b** starting from known compound **12**^24^ is shown in Fig. 2b. Removal of the Boc group under acidic conditions provided the corresponding amine, which was treated with the appropriate isocyanates (C_11_H_23_NCO or C_5_H_11_NCO) to give protected urea derivatives **13a** and **13b**. Then, the protected urea derivatives were converted to desired products **5a** and **5b**. Next, we examined the synthesis of carbonyl derivatives **7a** and **7b** (Fig. 2c). The required fatty acids containing carbonyl groups were prepared from known dicarboxylic acid derivative **14**^26^. After the activation of mono-acid **14** with *i*-BuOCOCl, treatment with methylenetriphenylphosphorane gave the corresponding phosphorane intermediate, which was reacted with the appropriate aldehydes to provide carbonyl compounds **15a** and **15b**^27^. Removal of the Bn groups and reduction of the alkene moieties furnished desired carbonyl-containing products **16a** and **16b**. With the desired fatty acid derivatives in hand, the sphingosine fragments were installed. The reduction of known azide **17**^28^ followed by condensation with **16a** and **16b** and removal of the Bn groups provided carbonyl derivatives **7a** and **7b**. Finally, we focused on the synthesis of the alkene-containing derivatives (Fig. 2d). We planned to selectively synthesize both *trans*/*cis*-alkene-containing derivatives **8a**, **8b**, **9a** and **9b** to examine the differences in the affinities of these isomers toward CD1d. Propargyl alcohol **19** was converted to alkynes **20a** and **20b** in moderate yields (4 steps)^29,30^. After investigating the *trans*-selective reduction of alkyne **20**, we found that the treatment of **20** with LiAlH_4_ in diglyme and THF at 130 °C gave the alcohol containing *trans*-alkene moieties^30^, and subsequent TEMPO oxidation afforded desired fatty acids **21a** and **21b**. In contrast, the *cis*-selective reduction of alkyne **20** was accomplished using nickel boride under a H_2_ atmosphere^31^. The subsequent removal of the TBS groups and TEMPO oxidation provided desired fatty acids **22a** and **22b**. With the alkene-containing fatty acids in hand, we next attempted to convert the glycolipid forms starting from known azide **17**. Cleavage of the Bn groups, reduction of the azide group and selective *N*-acylation with **21** and **22** in the presence of HATU furnished alkene-containing α-GalCer derivatives **8a**, **8b**, **9a** and **9b**.

### The immunological evaluation of the α-GalCer derivatives in vitro

With the α-GalCer derivatives in hand, we next performed in vitro immunological evaluations of the CD1d-NKT cell axis based on the following: (1) an antigen-presenting cell (APC)-free assay^32–34^, (2) a binding assay using AlphaScreen^24^, (3) the evaluation of cytokine production by mouse splenocytes. We used the APC-free assay to investigate the agonistic activities and binding characteristics of these compounds to CD1d. In particular, the stability of the ligand-CD1d complexes and the binding potential of the ligand-CD1d complex to NKT-TCR can be evaluated. The binding assay using AlphaScreen can reveal the binding affinities of these compounds for CD1d. The evaluation of cytokine production by mouse splenocytes was conducted to examine cytokine polarization through the CD1d-NKT cell system. The previously reported derivatives, amide-containing derivative **1** and amine-containing derivative **6**, were also evaluated in these assays to allow comparisons between different functional groups.

**Figure 3:**
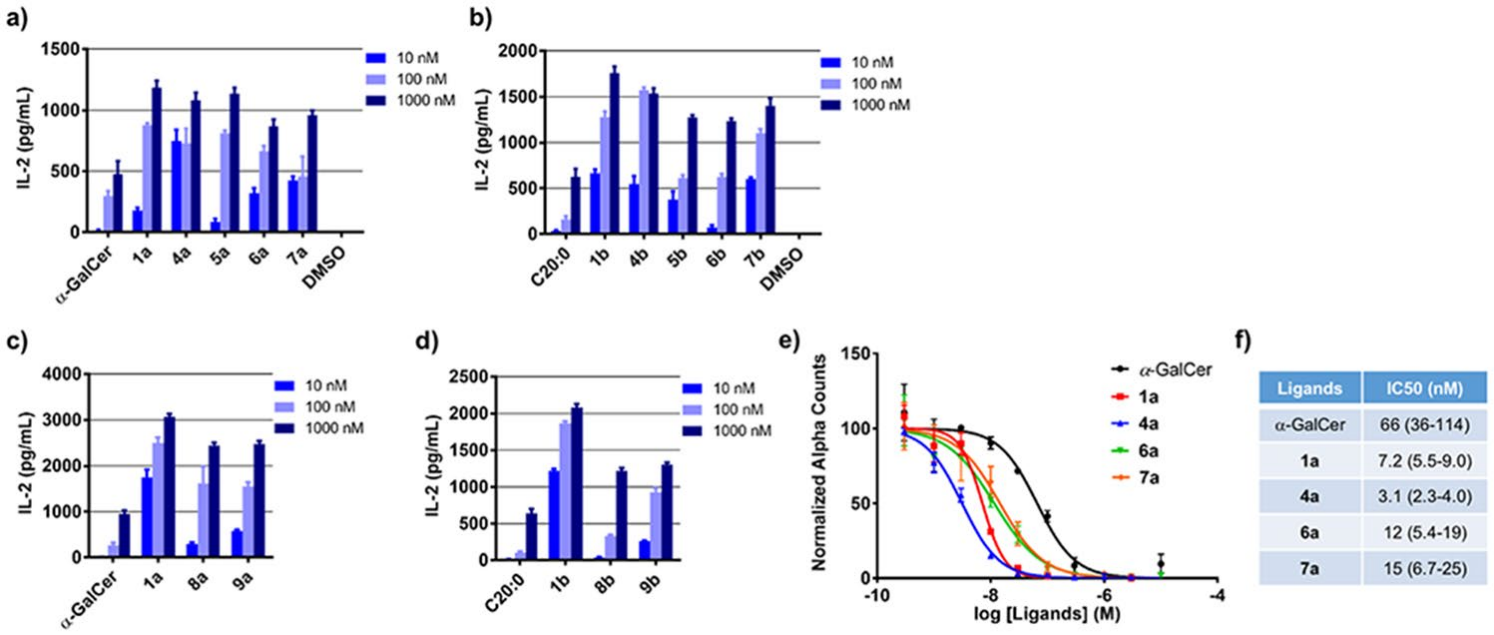
Binding potential of α-GalCer, C20:0 (α-GalCer-C20:0) and its analogue to mCD1d proteins. (**a**) Antigen presenting cell (APC)-free assay for lipid binding to mCD1d-Fc fusion protein using the indicated ligands (**1a**, **4a**‒**7a**). α-GalCer and C20:0 (α-GalCer-C20:0) were used as references. The graphs show the mean ± SEM of triplicate measurements, and the results shown are representative of at least three independent experiments. (**b**) APC-free assay using the indicated ligands (**1b**, **4b**‒**7b**). (**c**) APC-free assay using the indicated ligands (**1a**, **8a** and **9a**). (**d**) APC-free assay using the indicated ligands (**1b**, **8b** and **9b**). (**e**) AlphaScreen assay measuring the binding of α-GalCer and analogues (**1a**, **4a**, **6a** and **7a**) to mCD1d-Fc fusion protein. Each data point represents the mean ± SEM of triplicate measurements. (**f**) The IC_50_ values were calculated with the sigmoid dose–response formula using GraphPad Prism. The figures in brackets represent the 95% confidential interval (95% CI).

Initially, we conducted the APC-free assay using the synthesized α-GalCer derivatives (Fig. 3a–d). The APC-free assay with derivatives **1a**, **1b**, **2**, and **3** has already been reported, and the results demonstrated that all four derivatives resulted in higher levels of cytokine production relative to that caused by α-GalCer^23^.

The α-GalCer derivatives containing polar functional groups in their long fatty acyl chain (C26:0) were tested as shown in Fig. 3a. The introduction of any of the tested polar functional groups enhanced the cytokine production levels in a dose-dependent manner. Notably, the modification of the amide group to an *N*-methyl amide group (**4a**) led to an improvement in the cytokine production activity at 10 nM concentration (exact values of IL-2; **1a**: 177 ± 27 vs. **4a**: 749 ± 92). Urea-containing derivative **5a** showed activity equal to that of amide-containing derivative **1a** (**1a**: 177 ± 27 vs. **5a**: 85 ± 26 at 10 nM). The introduction of amine or carbonyl groups, which are considered to be more flexible than amide or urea groups, resulted in potencies slightly higher than those of amide- or urea-containing derivatives at 10 nM concentration (**1a**: 177 ± 27 vs. **6a**: 323 ± 41 and **7a**: 424 ± 32). Next, we examined the binding potential of derivatives **1a**, **4a**, **6a** and **7a** using AlphaScreen system previously reported by our group^24^. The dose response curves and IC50 values of these compounds are shown in Fig. 3e, f. All the polar functional group-containing derivatives exhibited higher binding affinities than that of α-GalCer. *N*-Methyl amide-containing derivative **4a** had a higher binding affinity for mCD1d. These results are consistent with those of the APC-free assay. In terms of the C20:0 α-GalCer derivatives, the introduction of polar functional groups significantly increased the cytokine production activities (Fig. 3b). Unlike the α-GalCer derivatives containing a long fatty acyl chain (C26:0), *N*-methyl amide-, urea-, and carbonyl-containing derivatives **4b**, **5b**, and **7b** exhibited activities comparable to that of amide-containing derivative **1b** (**1b**: 662 ± 46 vs. **4b**: 546 ± 87, **5b**: 376 ± 92 and **7b**: 600 ± 20 at 10 nM). Interestingly, carbonyl-containing derivative **7b** caused higher levels of cytokine production than did amine-containing derivative **6b** (**6b**: 74 ± 26 vs. **7b**: 600 ± 20 at 10 nM). These results support our previously reported MD simulation studies that suggested that the carbonyl group of the amide group is mainly associated with the Gln14 or Ser28 residues of mCD1d through hydrogen bond formation. The reduced activity of amine derivative **6b** might be due to its inability to form a strong hydrogen bonding network. Taken together, the agonistic activities and binding characteristics are dependent on the introduction of polar functional groups. Furthermore, the effects of each polar functional group on the activities varied depending on the length of the acyl chain.

We next investigated the relationship between the lipid conformation and the binding characteristics when using alkene-containing derivatives (Fig. 3c, d). Interestingly, the presence of alkene groups enhanced the levels of IL-2 production (α-GalCer: 274 ± 53 vs. **8a**: 1626 ± 371 and **9a**: 1556 ± 91 at 100 nM). Although significant differences in activities were not observed between the *trans*-alkenes and *cis*-alkenes, the *cis*-alkene-containing C20:0 derivatives tended to slightly increase cytokine production compared with the corresponding *trans*-alkene-containing derivative (**8b**: 334 ± 19 vs. **9b**: 925 ± 57 at 100 nM). The amide-containing derivative had higher levels of cytokine induction than the corresponding alkene- and alkane-containing derivatives (**1a**: 2,502 ± 114 vs. **8a**: 1626 ± 371 and α-GalCer: 274 ± 53 at 100 nM). These results indicated that lipid conformation could be an important factor for enhancing the stability of the ligand-CD1d complexes. Correspondingly, when considering that the introduction of an amide group to the acyl chain enhanced the cytokine production levels, the planarity of the amide bonds (conformational restriction of amides) could affect the CD1d recognition as well as the formation of hydrogen bonds between the amide group and the polar residues of CD1d, as supported by our previous reported computational studies^23^. Taken together, the order of the binding potentials upon introduction of various functional groups was *N*-methyl amide > amide > alkene > alkane. The binding of lipid acyl chains with amide groups to CD1d might be primarily affected by the rigidity and polarity of the chains.

In medicinal chemistry and chemical biology fields, the development and application of “isosteres” is important in the design of drug candidates and chemical probes. The “isosteres” can improve potency, alter physical properties, reduce metabolism, etc.^35^. For example, isosteres of peptide bonds (amide bonds) have been extensively studied^36,37^. Among these groups, alkenes have been designed and used as amide bond isosteres, focusing on the planar structure of amide bonds, and the introduction of alkenes can solve metabolism issues and reduce polarity^38,39^. In contrast, in this context, our designed amide- or *N*-methyl amide-containing lipids might be considered isosteres of alkene-containing lipids such as unsaturated lipids. The insertion of amide bonds into lipid chain would likely improve physical properties, including solubility, and make it easier to synthesize the desired functional lipids than the corresponding alkene-containing lipids. In fact, in our synthesis of the designed alkene-containing ligands, at least 5 steps were required to obtain the desired alkene-containing lipid, and several of these steps used toxic or flammable reagents (Fig. 2d). As such, the difficult syntheses of these lipids would impede SAR studies. In contrast, in most cases, the amide-containing derivatives can obtained in only a few steps, including a condensation of commercially available amino acids (Fig. 2).

**Figure 4:**
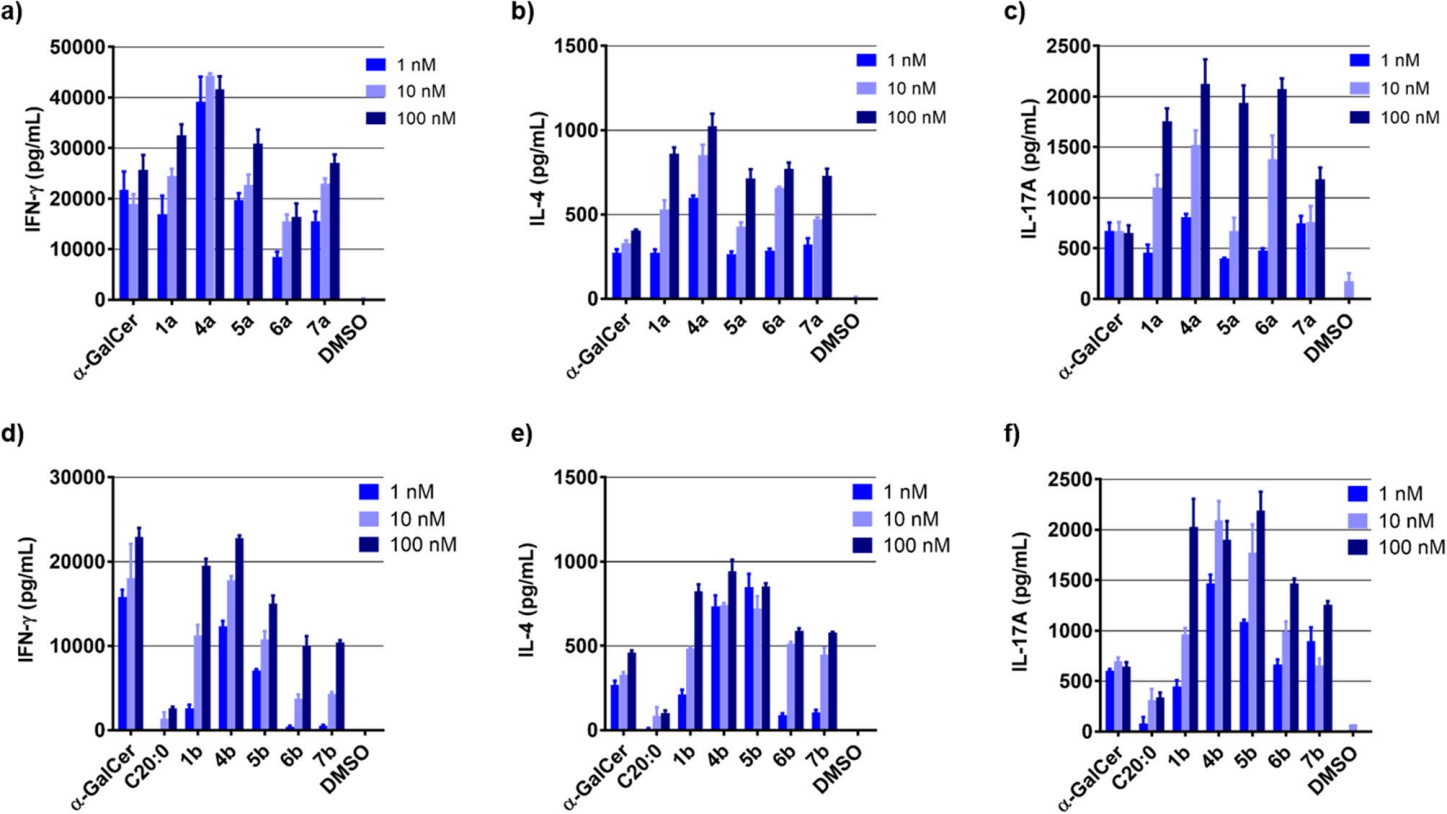
IFN-γ, IL-4 and IL-17A secretion by mouse splenocytes following stimulation by α-GalCer, C20:0 (α-GalCer-C20:0) or its analogues (**4**‒**7**). The graphs show the mean ± SEM of triplicate measurements, and the results shown are representative of two or three independent experiments. (**a**), (**d**) IFN-γ secretion, (**b**), (**e**) IL-4 secretion, and (**c**), (**f**) IL-17A secretion.

**Figure 5:**
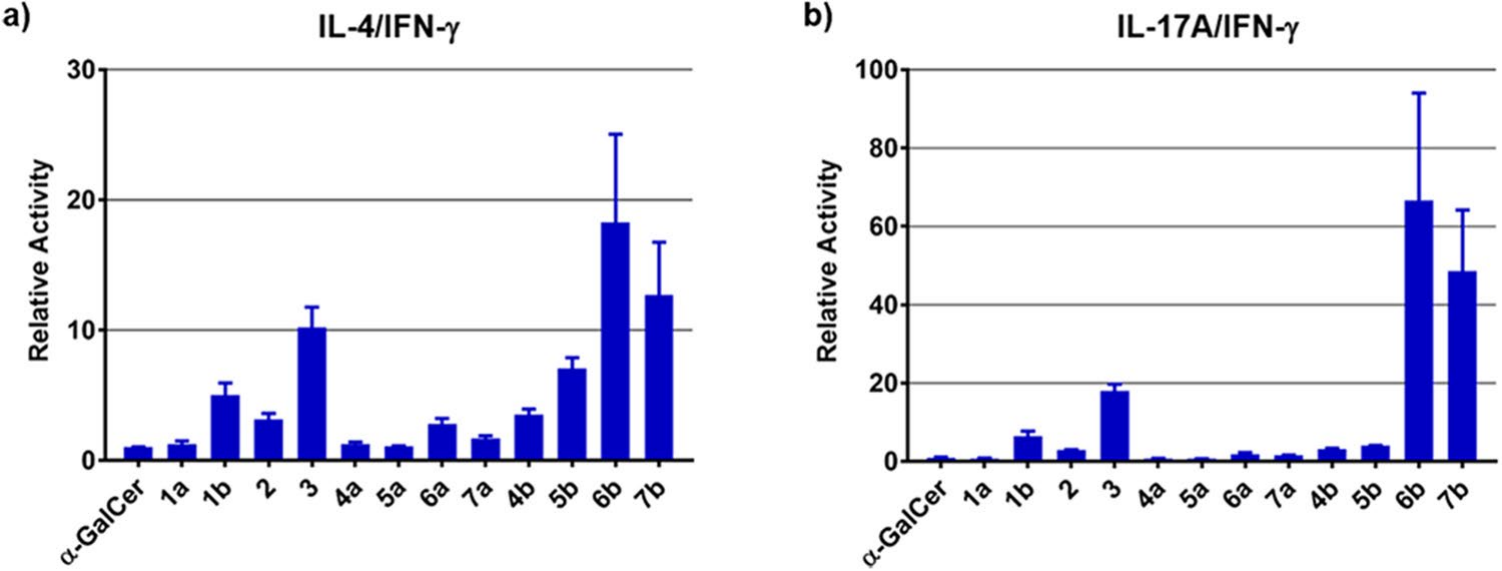
The relative ratios of cytokine production with the analogues compared with α-GalCer, at 1 nM concentrations (Figs. 4 and S2) are summarized (the relative ratios at 10 and 100 nM are shown in Fig S3). The graphs show the mean ± SEM of triplicate measurements, and the results shown are representative of two or three independent experiments. (**a**) Relative ratios (IL-4/IFN-γ) of cytokine production with the analogues compared to α-GalCer, all at 1 nM. (**b**) Relative ratios (IL-17/IFN-γ) of cytokine production with the analogues compared to α-GalCer, all at 1 nM.

We next studied the in vitro splenocyte cytokine polarization from C57BL/6 mice. The supernatant IFN-γ, IL-4 and IL-17A levels after 48 h of treatment with the synthesized derivatives were quantified (Figs. 4 and S2). Initially, we evaluated derivatives **1a**, **1b**, **2**, and **3**, and α-GalCer as reference (Figs. S2a–c and 5). We measured IFN-γ, IL-4 and IL-17A with these derivatives. IFN-γ, IL-4 and IL-17A were recovered and measured from the supernatant of a ligand-stimulated splenocyte (We previously reported the IFN-γ and IL-4 production levels with derivatives **1a**, **1b**, **2**, and **3**)^23^. As shown in Fig. S2a–c, the IFN-γ, IL-4 and IL-17A production levels induced by the amide and bisamide derivatives (**1a** and **2**) were higher than or equal to that of α-GalCer (IFN-γ; α-GalCer: 12,297 ± 61 vs. **1a**: 9,152 ± 373 and **2**: 9,467 ± 362, IL-4; α-GalCer: 247 ± 14 vs. **1a**: 232 ± 41 and **2**: 593 ± 65, IL-17A; α-GalCer: 2,258 ± 242 vs. **1a**: 1,276 ± 60 and **2**: 5,179 ± 154 at 1 nM). In contrast, the C20:0 amide and Bz amide derivatives (**1b** and **3**) produced lower levels of IFN-γ (Fig. S2a, α-GalCer: 12,297 ± 61 vs. **1b**: 822 ± 11 and **3**: 514 ± 79 at 1 nM), and induced IL-17A production comparable to that of α-GalCer (Fig. S2c, α-GalCer: 2,258 ± 242 vs. **1b**: 976 ± 207 and **3**: 1651 ± 127 at 1 nM). Next, we tested CD1d ligands containing polar functional groups (**4a–7a**, Fig. 4a–c), and their corresponding C20:0 type ligands **4b–7b** (Fig. 4d–f). Interestingly, *N*-methyl amide-containing ligand **4a** was more potent than the other derivatives (IFN-γ: 39,159 ± 4,960, IL-4: 599 ± 13 at 1 nM). In contrast, ligand **6a**, with an amine as the polar functional group, showed slightly lower IFN-γ production and exhibited an IL-4-biasing response relative to corresponding amide-containing ligand **1a** (IFN-γ; **1a**: 16,907 ± 3,679 vs. **6a**: 8,515 ± 1,016, IL-4; **1a**: 247 ± 19 vs. **6a**: 286 ± 12 at 1 nM), as previously reported^24^. Among C20:0-type ligands **4b**–**7b**, the introduction of *N*-methyl amide and urea groups markedly increased the levels of IFN-γ, IL-4 and IL-17A production compared with the corresponding C20:0-α-GalCer Fig. 4d–f, IFN-γ; C20:0-α-GalCer: 1,353 ± 731 vs. **4b**: 17,753 ± 542 and **5b**: 10,799 ± 950, IL-4; C20:0-α-GalCer: 85 ± 52 vs. **4b**: 743 ± 11 and **5b**: 723 ± 73, IL-17A; C20:0-α-GalCer: 696 ± 38 vs. **4b**: 2095 ± 190 and **5b**: 1774 ± 278 at 10 nM). In contrast, switching from an amide to an amine or a carbonyl group was associated with slight decrease in potency (IFN-γ; **6b**: 3,764 ± 450 and **7b**: 4,276 ± 261, IL-4; **6b**: 513 ± 11 and **7b**: 448 ± 40, IL-17A; **6b**: 990 ± 101 and **7b**: 656 ± 69 at 10 nM). Furthermore, based on each cytokine production (Figs. 4 and S2), we calculated the relative ratios of cytokine production with the analogues compared with α-GalCer as shown in Figs. 5 and S3. The relative ratios of cytokine production at 1 and 10 nM displayed the similar tendency, whereas comparing relative ratios at 100 nM was difficult because the cytokine production level could reach at saturated levels at 100 nM concentration of some derivatives. The C20:0 amide and Bz amide derivatives (**1b** and **3**) displayed IL-4- and IL-17A-biasing properties (Fig. 5, IL-4/IFN-γ; **1b**: 5.04 ± 0.90, **3**: 10.2 ± 1.6, IL-17A/IFN-γ; **1b**: 6.43 ± 1.27, **3**: 18.0 ± 1.7), while the amide and bisamide derivatives (**1a** and **2**) showed relatively less selective cytokine responses (Fig. 5, IL-4/IFN-γ; **1a**: 1.27 ± 0.23, **2**: 3.15 ± 0.46, IL-17A/IFN-γ; **1a**: 0.76 ± 0.06, **2**: 2.98 ± 0.03). Clearly, all C20:0-type ligands containing polar functional groups displayed IL-4 polarization, and ligands **6b** and **7b** had IL-4- and IL-17A-biasing properties (Fig. 5, IL-4/IFN-γ; **6b**: 18.3 ± 6.8, **7b**: 12.7 ± 4.0, IL-17A/IFN-γ; **6b**: 66.7 ± 27.4, **7b**: 48.6 ± 15.6). Taken together, we found that the use of different polar functional groups in the lipid chain could allow the control of cytokine production levels and their balance (Th1/Th2/Th17). In particular, the introduction of *N*-methyl amide tended to increase cytokine production levels. Bz amide-containing ligand **3** and more flexible ligands **6b** and **7b** (e.g. amine and carbonyl groups) did bias the immune response toward Th2 and Th17.

### Evaluation of water solubility and metabolic stability in vitro and phamacokinetics (PK) profiles

With the in vitro immunological data in hand, we next evaluated the water solubility and metabolic stability of the α-GalCer derivatives (Table S1) to select compounds for further in vivo evaluation. Although many SAR studies focusing on α-GalCer as a CD1d ligand have been reported, little information is available about their ADME (absorption, distribution, metabolism and excretion) properties. Moreover, α-GalCer is known to exhibit limited solubility in aqueous solvents due to the high lipophilicity of its two long lipid chains. Indeed, α-GalCer is extremely insoluble in aqueous buffer, and even DMSO. Thus, it requires specialized techniques, such as sonication or heating, to obtain reproducible results. On the other hand, our newly deigned ligands were expected to offer better water solubility due to the introduction of polar functional groups into the hydrophobic lipid chains. The solubilities of α-GalCer and a series of α-GalCer derivatives were evaluated (Table S1). Some compounds, including α-GalCer, showed poor solubility in this examination (< 5 μM), meaning that their levels were below the detection limits. The C20:0 amide- and Bz amide-containing derivatives (**1b** and **3**) exhibited measurable solubilities (5 and 7.2 μM, respectively). Next, the metabolic stabilities of the derivatives were tested using human and rat liver microsome assays (Table S1). Although the C26:0 amide-, C20:0 amide- and amine-containing derivatives (**1a**, **1b** and **6a**) displayed good metabolic stability for both species, the *N*-methyl amide derivative (**4a**) was metabolized more rapidly. The bisamide- and Bz amide-containing derivatives (**2** and **3**) were resistant to rat microsomal metabolism.

**Figure 6:**
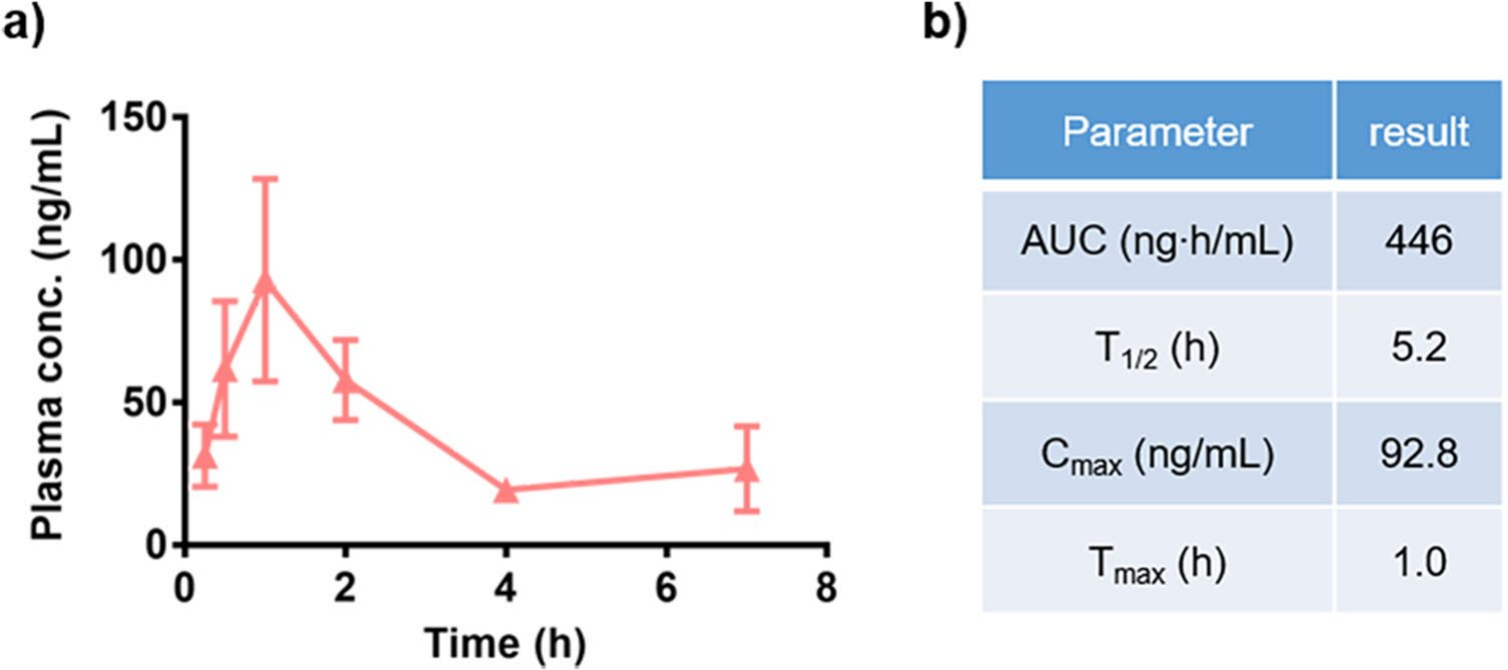
Plasma pharmacokinetic (PK) profiles of **3** following 100 μg/kg ip dosing in C57BL/6J mice (n = 6). (**a**) Plasma concentration − time profiles of **3**. Data are mean ± SEM (n = 6). (**b**) PK parameter of **3**.

Next, the PK profile of the glycolopid ligand in mice was evaluated as shown in Fig. 6. A limited number of studies have reported on PK profiles of glycolipids including α-GalCer^40^. As shown in Fig. 6a, despite its relatively large size (MW = 781.1) and high lipophilicity, a measurable plasma concentration was achieved after intraperitoneal injection of 100 μg/kg of **3** (AUC = 466 ng∙h/mL; T_1/2_ = 5.2 h; T_max_ = 1.0 h).

### Efficacy of in vivo CD1d ligand treatment in DSS-induced colitis

To date, several researchers have revealed that NKT cells play an important role in intestinal inflammation^41,42^. NKT cell activation by CD1d ligands prevented the murine DSS model of UC, which is a clinical manifestation observed in chronic inflammatory diseases of the gastrointestinal (GI) tract (IBD)^43^. In particular, the potent CD1d ligands, α-GalCer and OCH (a representative Th2-biasing ligand) were effective in the murine DSS model^22^.

On the other hand, some groups have reported that IL-17A has protective effects in inflamed colonic tissue. For instance, Ogawa et al. reported that IL-17A neutralization exacerbates tissue damage in a DSS model^44^. Lee et al. demonstrated that IL-17A-dependent regulation supports barrier function during DSS injury^45^. Thus, we tested whether the polar functional group-containing α-GalCer derivatives that showed Th2- and Th17-biasing responses would display in vivo efficacy in a DSS model. The DSS-induced model based on oral DSS administration is widely used as an intestinal inflammatory model. With the results of the immunological activities and physiological properties in hand, we chose Bz amide-containing derivative **3** and determined the in vivo efficacy in a model of ulcerative colitis. The derivative **3** displayed Th2 and Th17-biasing responses (Figs. 4 and S2) and reasonable physicochemical properties relative to other derivatives, except for the suboptimal metabolic property for human liver microsome. The derivative **3** also demonstrated higher agonistic activity than α-GalCer using the APC-free assay as shown in our previous report^23^. Furthermore, from a synthetic point of view, the Bz amide-containing derivative **3** was the attractive candidate, because amide-containing derivatives are easier to synthesize than the amine- or carbonyl-containing derivatives (**6b** or **7b**) that also showed Th2 and Th17-biasing responses. Indeed, compared to the Bz amide-containing derivative **3**, two or more extra steps were required to synthesize the derivatives **6b** or **7b**. Additionally, we also selected bisamide-containing derivative **2** that displayed relatively less selective cytokine responses but potent cytokine induction activities.

**Figure 7:**
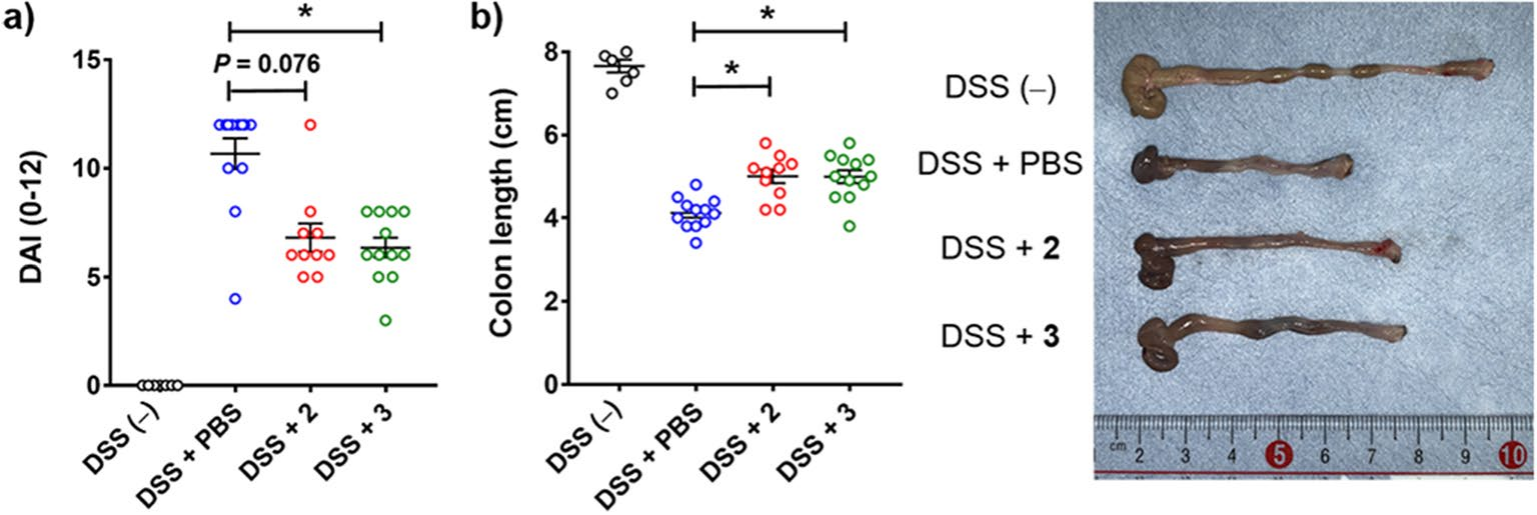
The immune protective effects of derivatives **2** and **3** against DSS-induced colitis. C57BL/6J mice (n = 12 per group) were continuously treated with 2.0% DSS in their drinking water from day 1 to 7. The mice were injected intraperitoneally with 100 μg/kg of the derivatives or PBS (control) on day 4. (**a**) The DAI scores compared with those of the mice administered PBS. (**b**) Comparison of the colon lengths on day 8 with those of the mice administered PBS. Data are mean ± SEM (DSS (−): n = 7, DSS + PBS: n = 12, DSS + derivative **2**: n = 10, derivative **3**: n = 12). *P < 0.05 compared with control. The DAI score and the colon length represent data for 10 mice, since two of twelve mice treated with bisamide-containing derivative **2** died by day 8.

The evaluation of the derivatives using DSS-induced model was carried out according to the procedure reported by Ueno et al.^22^ in which α-GalCer and OCH were administered intraperitoneally, and demonstrated their in vivo efficacy in the model. DSS treatment causes progressive weight loss, diarrhea and colon inflammation with a significant reduction in colon length, which leads to increases in the clinical scores of disease symptoms (DAI: disease activity index). C57BL/6 mice (n = 12 per group) were continuously treated with 2.0% DSS in their drinking water from day 1 to day 7. The mice were injected intraperitoneally with 100 μg/kg of the derivatives or PBS (control) on day 4. On day 8, the DAI scores and colon lengths of the mice administered the derivatives were compared with those of the mice administered PBS (control). Two out of twelve mice treated with bisamide-containing derivative **2** (less selective but potent ligand) had died by day 8. As shown in Fig. 7, treatment with the Bz amide-containing derivative **3** (Th2- and Th17-biasing ligand) resulted in a significant improvement in the DAI scores (*P* < 0.05). In contrast, treatment with the bisamide-containing derivative **2** did not significantly alter the DAI scores to control group. Colon shortening is a feature of colon inflammation in the DSS-induced model. In the model, the colon length of healthy mice is about 8 cm (DSS (−)), whereas that of mice upon DSS treatment is about 4 cm (DSS + PBS) as shown in Fig. 7b. Relative to PBS (control), treatment with both derivatives **2** and **3** significantly prevented the colon shortening caused by DSS (*P* < 0.05). As such, Th2- and Th17-biasing derivative **3** displayed protective effects against intestinal inflammation after a single intraperitoneal injection. In this study, we selected the intraperitoneal injection as the administration method of lipid ligands, and used DAI scores and colon length to investigate the protective effects of derivatives against colitis. On the other hand, the difference in the administration method of immunomodulators, such as lipid ligands, might have a significant impact on their efficacy^46,47^. In addition, the cytokine profiles in the colon upon ligand treatment could also provide useful information regarding their efficacy. Further research is currently being conducted into the effects of administration methods on efficacy and the examination of colon cytokine profiles.

## Conclusion

In this study, we conducted a detailed SAR study of α-GalCer derivatives containing various functional groups in their lipid acyl chains and identified several potent CD1d ligands that effected cytokine induction or showed cytokine polarization. In particular, the *N*-methyl amide-containing ligand is among the most potent CD1d ligands reported to date. The studies have indicated that the binding affinities and/or total cytokine production levels could depend on the flexibility of lipid part in addition to hydrophilic interactions (e.g. hydrogen bond formation). We also demonstrated the potential use of amide-containing lipids as the isosteres of alkene-containing lipids. Many alkene-containing lipid moieties, such as naturally occurring fatty acids and sphingosines, are known to exhibit activities distinct from those of the corresponding saturated lipids. Our approaches might be applicable to SAR studies to effectively identify chemical probes for elucidating the function of lipid binding proteins, or drug candidates.

In terms of cytokine polarization, most of the ligands containing polar functional groups displayed Th2- and Th17-biasing immune responses. Intriguingly, the ligands containing the Bz-amide group and flexible functional groups such as amine and carbonyl groups tended to show Th2- and Th17-biasing profiles.

Finally, based on our in vitro immunological evaluation and investigation of the physicochemical properties, we selected two ligands, bisamide- and Bz amide-containing derivatives **2** and **3**, and evaluated their in vivo efficacies in a DSS-induced model of UC. A single dose of derivative **3** (Th2- and Th17-biasing ligand) significantly attenuated the severity of disease in DSS-induced colitis. Therefore, our designed Th2- and Th17-biasing ligands could be used to regulate autoimmune disorders through the CD1d-NKT cell axis.

## Methods

### Lipid ligands

α-GalCer and its derivative (C20:0-α-GalCer) were prepared following a literature procedure^48^. The amide-containing ligands were synthesized as described in the Supplementary Information. All lipids were dissolved in DMSO and stored as aliquots at − 30 °C.

### Cell culture

NKT hybridoma 2E10 cells were cultured in RPMI-1640 (NACALAI TESQUE, INC) supplemented with 10% fetal bovine serum (FBS; Biowest), and 1% penicillin–streptomycin (Gibco).

### Cell-free assay for CD1d-lipid binding

For this assay, 96-well microplates (Bio-one 96-well cell culture plate, Greiner) were coated with mouse CD1d:Ig Fusion protein (BD Biosciences) (0.25 μg/well) in PBS at 37 °C for 24 h. After washing with PBS, the lipid ligands (100 nM) were added and incubated at 37 °C for 24 h. After washing, 2E10 NKT hybridoma cells (2.5 × 10^5^ cells/well) were added and cultured at 37 °C for 48 h. IL-2 release was measured with an ELISA kit (Affymetrix).

### AlphaScreen assay for mCD1d-lipid binding

The Mouse IgG Detection Kit (Perkin-Elmer Life Sciences) was used to determine CD1d–ligand interactions. Mouse CD1d:Ig fusion protein (10 μL/well, BD Biosciences, final concentration of 5 nM) in PBS containing 0.005% Tween 20 was mixed with anti-mouse IgG acceptor beads (10 μL/well, final concentration of 10 μg/mL) in PBS containing 0.005% Tween 20. After 60 min, biotinyl-PE (10 μL/well, Avanti, final concentration of 2 μM) in PBS containing 0.005% Tween 20, 5% DMSO and 0.5% EtOH), and α-GalCer analogues (10 μL/well, final concentration range from 0.0003 to 10 μM) in PBS containing 0.005% Tween 20 and 15% DMSO were added to the wells. After incubation at 37 °C for 18 h, streptavidin donor beads (10 μL/well, final concentration of 10 μg/mL) in PBS containing 0.005% Tween 20 were added, and the plates were incubated for another 60 min. Samples were measured at 520–620 nm with a Spark 10 M microplate reader (TECAN).

### Cytokine secretion assay using mouse splenocyte

Spleen cell suspensions (from C57BL/6 mice) were prepared in complete medium (RPMI 1,640 media supplemented with 10% FBS and 1% penicillin–streptomycin) and seeded into 96-well plates (6.0 × 10^5^ cells/well). Lipid ligands were added, and the mixture were incubated at 37 °C for 48 h. IFN-γ, IL-4 and IL-17A release were measured with an ELISA kit (Affymetrix).

### Pharmacokinetic profiles in mice

The pharmacokinetic study was approved by The Institutional Animal Care and Use Committee of Ono Pharmaceutical Co., Ltd. and was performed in accordance with the ethics criteria contained in the animal welfare bylaws of the Committee. The pharmacokinetics of **3** were studied following intraperitoneal administration. The 10 μg/mL dosing solution, which contained DMSO/Phosphate buffered saline = 1/9, was administered intraperitoneally to six male C57BL/6 mice (Charles River Laboratories, Japan, Inc.). Blood samples were collected into heparinized syringes at designated times and centrifuged to obtain the plasma samples. The plasma samples were assayed after protein precipitation by acetonitrile/ethanol (70/30, v/v), followed by HPLC/MS/MS analysis. Plasma concentration–time data were analyzed by noncompartmental methods.

### Efficacy of in vivo CD1d ligand treatment in DSS-induced colitis

The animal experiments using murine DSS model were conducted in accordance with the guidelines of Keio university institutional animal care and use committee. Specific pathogen-free C57BL/6 mice were purchased from Japan Clea (Tokyo, Japan). All mice were housed under specific pathogen-free conditions in microisolator cages in an animal facility, and male mice (7 weeks of age) were used. DSS (dextran sulfate sodium) was added to the water supply provided to the animals at a concentration of 2.0% (wt/vol) for days 1–7. Derivatives **2** and **3** were first dissolved in DMSO at 100 μg/mL and then diluted in PBS solution. The derivatives (100 μg/kg in 200 μL of solution) were injected intraperitoneally on day 4 after the induction of colitis. Control animals received 200 μL of PBS solution containing the same concentration of DMSO. Mice were sacrificed on day 8 after DSS administration. Intestinal tissues were removed and opened longitudinally. The length of the colon was measured after exclusion of cecum. The disease activity index (DAI), defined as stool blood, stool form and weight loss, and the length of the colon were used to analyze the therapeutic benefits of derivatives **2** and **3**.

### Statistical analysis

All the results were expressed as the mean ± SEM. All statistical analyses were performed with GraphPad Prism 7.0 software package. Statistical analyses of data were performed by using the one-way ANOVA test when more than 2 groups were compared with post-hoc analysis (Tukey’s Test) to determine statistical significance. Statistical significance was set at a p value of less than 0.05.

### Reporting summary

Further information on the research design is available in the Nature Research Reporting Summary linked in this article.

## Supplementary information

Supplementary information

## Data Availability

The authors declare that the data supporting the findings of this study are available within the article (and its Supplementary Information files).

## References

[1] Brennan PJ, Brigl M, Brenner MB (2013). Invariant natural killer T cells: An innate activation scheme linked to diverse effector functions. Nat. Rev. Immunol..

[2] Shissler SC, Webb TJ (2019). The ins and outs of type I iNKT cell development. Mol. Immunol..

[3] Wolf BJ, Choi JE, Exley MA (2018). Novel approaches to exploiting invariant NKT cells in cancer immunotherapy. Front. Immunol..

[4] Bae EA, Seo H, Kim IK, Jeon I, Kang CY (2019). Roles of NKT cells in cancer immunotherapy. Arch. Pharm. Res..

[5] Juno JA, Keynan Y, Fowke KR (2012). Invariant NKT cells: Regulation and function during viral infection. PLoS Pathog..

[6] Gaya M, Barral P, Burbage M, Aggarwal S, Montaner B, Warren Navia A, Aid M, Tsui C, Maldonado P, Nair U, Ghneim K, Fallon PG, Sekaly RP, Barouch DH, Shalek AK, Bruckbauer A, Strid J, Batista FD (2018). 2018 Initiation of antiviral B cell immunity relies on innate signals from spatially positioned NKT cells. Cell.

[7] Linsen L, Somers V, Stinissen P (2005). Immunoregulation of autoimmunity by natural killer T cells. Hum Immunol.

[8] Novak J, Lehuen A (2011). Mechanism of regulation of autoimmunity by iNKT cells. Cytokine.

[9] Van Kaer L, Wu L (2018). Therapeutic potential of invariant natural killer T cells in autoimmunity. Front. Immunol..

[10] Kinjo Y, Takatsuka S, Kitano N, Kawakubo S, Abe M, Ueno K, Miyazaki Y (2018). Functions of CD1d-restricted invariant natural killer T cells in antimicrobial immunity and potential applications for infection control. Front. Immunol..

[11] Kim BS, Park YJ, Chung Y (2016). Targeting IL-17 in autoimmunity and inflammation. Arch. Pharm. Res..

[12] Veldhoen M (2017). Interleukin 17 is a chief orchestrator of immunity. Nat. Immunol..

[13] McGeachy MJ, Cua DJ, Gaffen SL (2019). The IL-17 family of cytokines in health and disease. Immunity.

[14] Laurent X, Bertin B, Renault N, Farce A, Speca S, Milhomme O, Millet R, Desreumaux P, Henon E, Chavatte P (2014). Switching invariant natural killer T (iNKT) cell response from anticancerous to anti-inflammatory effect: Molecular bases. J. Med. Chem..

[15] O'Shea JJ, Murray PJ (2008). Cytokine signaling modules in inflammatory responses. Immunity.

[16] Neurath MF (2014). Cytokines in inflammatory bowel disease. Nat. Rev. Immunol..

[17] Becher B, Spath S, Goverman J (2017). Cytokine networks in neuroinflammation. Nat. Rev. Immunol..

[18] Morita M, Motoki K, Akimoto K, Natori T, Sakai T, Sawa E, Yamaji K, Koezuka Y, Kobayashi E, Fukushima H (1995). Structure–activity relationship of Alpha-Galactosylceramides against B16-bearing mice. J. Med. Chem..

[19] Li X, Fujio M, Imamura M, Wu D, Vasan S, Wong C-H, Ho DD, Tsuji M (2010). Design of a potent CD1d-binding NKT cell ligand as a vaccine adjuvant. Proc. Natl. Acad. Sci. USA.

[20] MIyamoto K, MIyake S, Yamamura T (2001). A synthetic glycolipid prevents autoimmune encephalomyelitis by inducing TH2 bias of natural killer T cells. Nature.

[21] Kim Y, Oh K, Song H, Lee DS, Park SB (2013). Synthesis and biological evaluation of alpha-galactosylceramide analogues with heteroaromatic rings and varying positions of a phenyl group in the sphingosine backbone. J. Med. Chem..

[22] Ueno Y, Tanaka S, Sumii M, Miyake S, Tazuma S, Taniguchi M, Yamamura T, Chayama K (2005). Single dose of OCH improves mucosal T Helper Type 1/T Helper Type 2 cytokine balance and prevents experimental colitis in the presence of Va14 Natural Killer T cells in mice. Inflamm. Bowel Dis..

[23] Inuki S, Aiba T, Hirata N, Ichihara O, Yoshidome D, Kita S, Maenaka K, Fukase K, Fujimoto Y (2016). Isolated polar amino acid residues modulate lipid binding in the large hydrophobic cavity of CD1d. ACS Chem. Biol..

[24] Inuki S, Kashiwabara E, Hirata N, Kishi J, Nabika E, Fujimoto Y (2018). Potent Th2 cytokine bias of natural killer T cell by CD1d glycolipid ligands: Anchoring effect of polar groups in the lipid component. Angew. Chem. Int. Ed..

[25] Kishi J, Inuki S, Hirata N, Kashiwabara E, Yoshidome D, Ichihara O, Fujimoto Y (2019). Structure–activity relationship studies of Bz amide-containing alpha-GalCer derivatives as natural killer T cell modulators. Bioorg. Med. Chem. Lett..

[26] Gokel G, Curvey N, Luderer S, Walker J (2014). Improved syntheses of benzyl hydraphile synthetic cation-conducting channels. Synthesis.

[27] Keen SP, Cowden CJ, Bishop BC, Brands KMJ, Davies AJ, Dolling UH, Lieberman DR, Stewart GW (2005). Practical asymmetric synthesis of a non-peptidic alpha(v)beta(3) antagonist. J. Org. Chem..

[28] Fan G-T, Pan Y-S, Lu K-C, Cheng Y-P, Lin W-C, Lin S, Lin C-H, Wong C-H, Fang J-M, Lin C-C (2005). Synthesis of α-galactosyl ceramide and the related glycolipids for evaluation of their activities on mouse splenocytes. Tetrahedron.

[29] Brown CA, Yamashita A (1975). Saline hydrides and superbases in organic reactions. 9. Acetylene zipper—Exceptionally facile contrathermodynamic multipositional isomerization of alkynes with potassium 3-aminopropylamide. J. Am. Chem. Soc..

[30] Shikichi Y, Akasaka K, Tamogami S, Shankar S, Yew JY, Mori K (2012). Pheromone synthesis. Part 250: Determination of the stereostructure of CH503, a sex pheromone of male Drosophila melanogaster, as (3R,11Z,19Z)-3-acetoxy-11,19-octacosadien-1-ol by synthesis and chromatographic analysis of its eight isomers. Tetrahedron.

[31] Gansäuer A, Fan C-A, Keller F, Keil J (2007). Titanocene-catalyzed regiodivergent epoxide openings. J. Am. Chem. Soc..

[32] Sidobre S, Hammond KJ, Benazet-Sidobre L, Maltsev SD, Richardson SK, Ndonye RM, Howell AR, Sakai T, Besra GS, Porcelli SA, Kronenberg M (2004). The T cell antigen receptor expressed by Valpha14i NKT cells has a unique mode of glycosphingolipid antigen recognition. Proc. Natl. Acad. Sci. USA.

[33] Li X, Shiratsuchi T, Chen G, Dellabona P, Casorati G, Franck RW, Tsuji M (2009). Invariant TCR rather than CD1d shapes the preferential activities of C-glycoside analogues against human versus murine invariant NKT cells. J. Immunol..

[34] Zeissig S, Olszak T, Melum E, Blumberg RS (2013). Analyzing antigen recognition by natural killer T cells. Methods Mol. Biol..

[35] Meanwell NA (2011). Synopsis of some recent tactical application of bioisosteres in drug design. J. Med. Chem..

[36] Avan I, Hall CD, Katritzky AR (2014). Peptidomimetics via modifications of amino acids and peptide bonds. Chem. Soc. Rev..

[37] Qvit N, Rubin SJS, Urban TJ, Mochly-Rosen D, Gross ER (2017). Peptidomimetic therapeutics: scientific approaches and opportunities. Drug Discov. Today.

[38] Tomita K, Oishi S, Ohno H, Peiper SC, Fujii N (2008). Development of novel G-protein-coupled receptor 54 agonists with resistance to degradation by matrix metalloproteinase. J. Med. Chem..

[39] Kobayashi K, Oishi S, Hayashi R, Tomita K, Kubo T, Tanahara N, Ohno H, Yoshikawa Y, Furuya T, Hoshino M, Fujii N (2012). Structure–activity relationship study of a CXC chemokine receptor type 4 antagonist, FC131, using a series of alkene dipeptide isosteres. J. Med. Chem..

[40] Crul M, Mathot RA, Giaccone G, Punt CA, Rosing H, Hillebrand MX, Ando Y, Nishi N, Tanaka H, Schellens JM, Beijnen JH (2002). Population pharmacokinetics of the novel anticancer agent KRN7000. Cancer Chemother. Pharmacol..

[41] Wilson MT, Van Kaer L (2003). Natural killer T cells as targets for therapeutic intervention in autoimmune diseases. Curr. Pharm. Design..

[42] Friedrich M, Pohin M, Powrie F (2019). Cytokine networks in the pathophysiology of inflammatory bowel disease. Immunity.

[43] de Mattos BR, Garcia MP, Nogueira JB, Paiatto LN, Albuquerque CG, Souza CL, Fernandes LG, Tamashiro WM, Simioni PU (2015). Inflammatory bowel disease: An overview of immune mechanisms and biological treatments. Mediators. Inflamm..

[44] Ogawa A, Andoh A, Araki Y, Bamba T, Fujiyama Y (2004). Neutralization of interleukin-17 aggravates dextran sulfate sodium-induced colitis in mice. Clin. Immunol..

[45] Lee JS, Tato CM, Joyce-Shaikh B, Gulen MF, Cayatte C, Chen Y, Blumenschein WM, Judo M, Ayanoglu G, McClanahan TK, Li X, Cua DJ (2015). Interleukin-23-independent IL-17 production regulates intestinal epithelial permeability. Immunity.

[46] Furlan R, Bergami A, Cantarella D, Brambilla E, Taniguchi M, Dellabona P, Casorati G, Martino G (2003). Activation of invariant NKT cells by alphaGalCer administration protects mice from MOG35-55-induced EAE: Critical roles for administration route and IFN-gamma. Eur. J. Immunol..

[47] Nicol AJ, Tazbirkova A, Nieda M (2011). Comparison of clinical and immunological effects of intravenous and intradermal administration of alpha-galactosylceramide (KRN7000)-pulsed dendritic cells. Clin. Cancer. Res..

[48] Du W, Gervay-Hague J (2005). Efficient synthesis of alpha-galactosyl ceramide analogues using glycosyl iodide donors. Org. Lett..

