# Supplementary material for: Polar functional group-containing glycolipid CD1d ligands modulate cytokine-biasing responses and prevent experimental colitis
Source: Sci Rep. 2020 Sep 25;10:15766. doi: 10.1038/s41598-020-72280-4 (PMC7519074; doi:10.1038/s41598-020-72280-4)

Supporting Information

Polar Functional Group-Containing Glycolipid CD1d Ligands Modulate Cytokine-Biasing Responses and Prevent Experimental Colitis

Shinsuke Inuki,[1,2] Natsumi Hirata,[1] Emi Kashiwabara,[1] Junichiro Kishi,[1] Toshihiko Aiba,[1,3] Toshiaki Teratani,[4] Wataru Nakamura,[5] Yoshimi Kojima,[5] Toru Maruyama,[5] Takanori Kanai,[4] Yukari Fujimoto*,[1]

[1]*Graduate School of Science and Technology, Keio University, Hiyoshi, Kohoku-ku, Yokohama, Kanagawa 223-8522, Japan*

[2]*Graduate School of Pharmaceutical Sciences, Kyoto University, Sakyo-ku, Kyoto 606-8501 Japan*

[3]*Department of Chemistry, Graduate School of Science, Osaka University, Machikaneyama-cho, Toyonaka, Osaka 560-0043, Japan*

[4]*School of Medicine, Keio University, Tokyo, Shinanomachi, Shinjuku-ku, Tokyo 160-8582, Japan*

[5]*Discovery & Research, ONO Pharmaceutical Co., Ltd. Sakurai, Shimamoto, Mishima, Osaka 618-8585, Japan*

E-mail: fujimotoy@chem.keio.ac.jp

Table of Contents

Supplemental Figure S2
Experimental Section S4
NMR Spectra S18

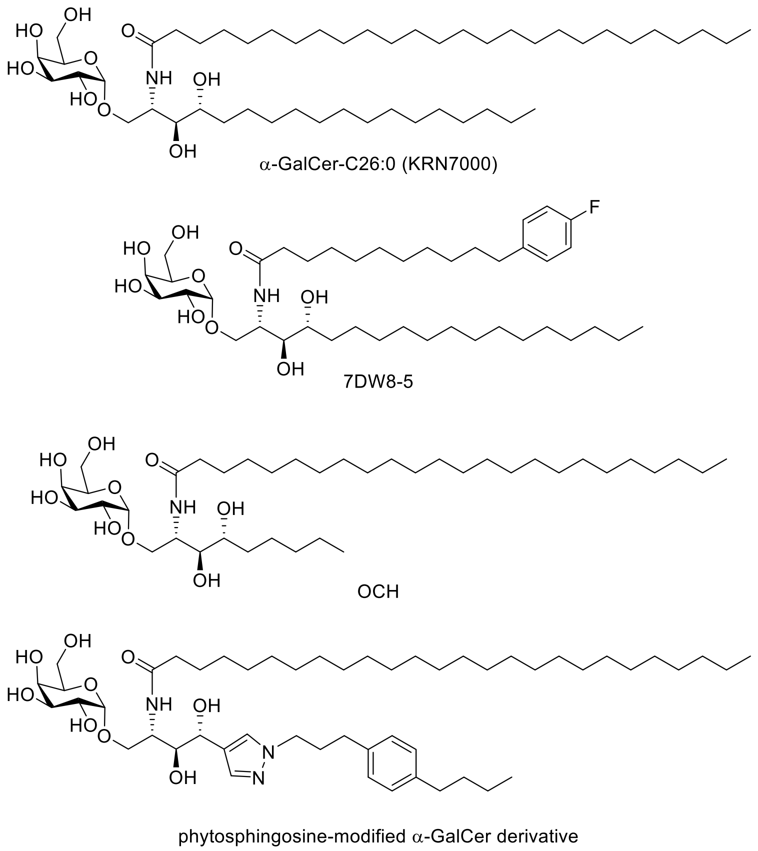

Figure S1. Structure of α-GalCer and its previously reported analogues.

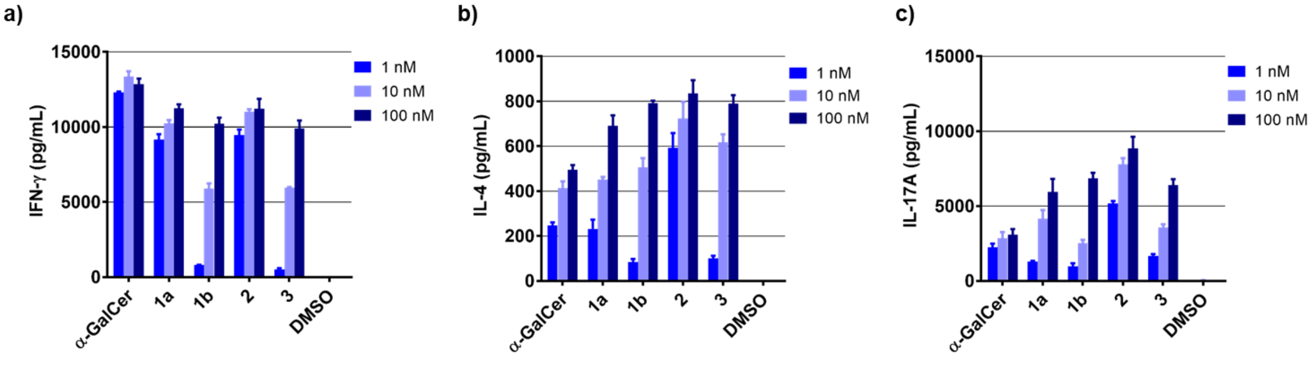

Figure S2. IFN-γ, IL-4 and IL-17A secretion by mouse splenocytes following stimulation by α-GalCer or its analogues **1**–**3**. The graphs show the mean ± SEM of triplicate measurements, and the results shown are representative of two or three independent experiments. (a) IFN-γ secretion, (b) IL-4 secretion, and (c) IL-17A secretion.

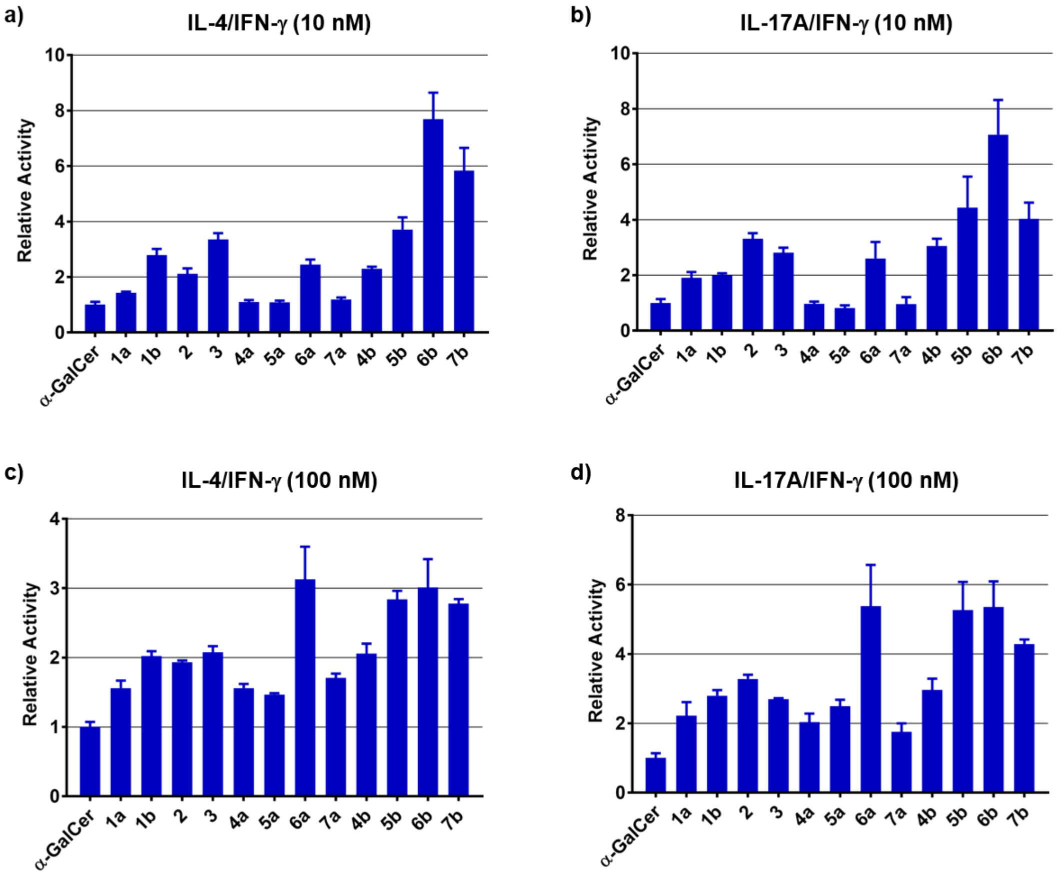

Figure S3. The relative ratios of cytokine production with the analogues compared with α-GalCer at 10 and 100 nM concentrations (Figs. 4 and S2) are summarized. The graphs show the mean ± SEM of triplicate measurements, and the results shown are representative of two or three independent experiments. Relative ratios (IL-4/IFN-γ) of cytokine production with the analogues compared to α-GalCer, all at (a) 10 nM, (c) 100 nM. Relative ratios (IL-17/IFN-γ) of cytokine production with the analogues compared to α-GalCer, all at (b) 10 nM, (d) 100 nM.

Table S1. Physicochemical properties of the polar functional group-containing α-GalCer derivatives.

Compounds	solubility (μM)	metabolic stability (liver microsome, $T_{1/2}$)	
		human (min)	rat (min)
α-GalCer	-	-	-
**1a**	-	60	47
**1b**	5.0	>200	>200
**2**	-	<15	64
**3**	7.2	<15	169
**4a**	-	15	23
**6a**	-	>200	>200

Experimental Section

General Methods. All moisture-sensitive reactions were performed using syringe-septum cap techniques under an argon atmosphere and all glassware was dried in an oven at 80 °C for 2 h prior to use. Reactions at –78 °C employed a CO_2–acetone bath. Analytical thin layer chromatography (TLC) was performed on Silica gel 60 F_{254} Plates (Merck, 0.25 mm thickness). For flash chromatography, Silica gel 60 N [spherical neutral (Kanto Chemical Co., 40-50 μm)] was employed. All NMR spectral data were recorded on a JEOL ECX-400 spectrometer for ^1H (400 MHz) and ^{13}C (100 MHz). Chemical shifts are reported in δ (ppm) relative to TMS in $CDCl_3$ as internal standard (^1H NMR) or the residual $CHCl_3$ signal (^{13}C NMR). ^1H NMR spectra are tabulated as follows: chemical shift, multiplicity (b = broad, s = singlet, d = doublet, t = triplet, q = quartet, m = multiplet), number of protons, and coupling constant(s). Exact mass (HRMS) spectra were recorded on an electronspray ionization quadrupole time of flight (ESI-QTOF) mass spectrometer (micrOTOF-QII-HC; BRUKER).

Synthesis of Compound 11a.

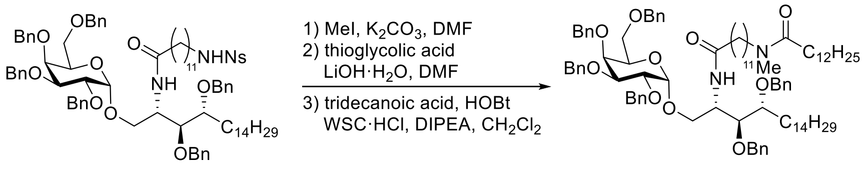

10 **11a**

To a stirred solution of **10** (22.5 mg, 0.0160 mmol) in DMF (1.0 mL) were added MeI (24.0 μL, 0.385 mmol) and K_2CO_3 (133 mg, 0.962 mmol). After being stirred overnight at room temperature, the mixture was diluted with Et_2O. The whole was washed with H_2O and brine, dried over Na_2SO_4, and concentrated under reduced pressure to give an oily residue. The residue (10.0 mg) was dissolved in DMF (700 μL). $LiOH \cdot H_2O$ (29.5 mg, 0.705 mmol) and thioglycolic acid (9.60 μL, 0.141 mmol) were added to the stirred mixture. After being stirred at room temperature for 9.5 h, the mixture was diluted with EtOAc. The whole was washed with saturated aqueous NH_4Cl, saturated aqueous $NaHCO_3$ and brine, dried over Na_2SO_4, and concentrated under reduced pressure to give an oily residue. The residue was dissolved in CH_2Cl_2 (355 μL). Tridecanoic acid (22.7 mg, 0.106 mmol), HOBt (14.7 mg, 0.109 mmol), WSC·HCl (20.3 mg, 0.108 mmol) and DIPEA (22.1 μL, 0.127 mmol) were added to the stirred mixture at 0 °C. After being stirred overnight at room temperature, the mixture was diluted with CH_2Cl_2. The whole was washed with saturated aqueous $NaHCO_3$, dried over Na_2SO_4, and concentrated under reduced pressure to give an oily residue, which was purified by column chromatography over silica gel with *n*-hexane-EtOAc (4:1) to give **11a** as colorless oil (5.0 mg, 50% yield): ^1H-NMR (400 MHz, $CDCl_3$) δ: 0.88 (t, J = 6.7 Hz, 6H), 1.22-1.30 (m, 56H), 1.41-1.54 (m, 6H), 1.60-1.65 (m, 2H), 1.86-1.98 (m, 2H), 2.25-2.30 (m, 2H), 2.90-2.95 (m, 3H), 3.20-3.24 (m, 1H), 3.31-3.35 (m, 1H), 3.38-3.42 (m, 1H), 3.47-3.51 (m, 2H), 3.73 (dd, J = 10.8, 3.6 Hz, 1H), 3.84-3.88 (m, 2H), 3.90-3.94 (m, 2H), 4.00-4.06 (m, 2H), 4.13-4.18 (m, 1H), 4.36 (d, J = 11.7 Hz, 1H), 4.42 (d, J = 11.7 Hz, 1H), 4.45-4.60 (m, 4H), 4.64 (d, J = 11.7 Hz, 1H), 4.71-4.82 (m, 4H), 4.84 (d, J = 3.6 Hz, 1H), 4.92 (d, J = 11.7 Hz, 1H), 6.11-6.14 (m, 1H), 7.21-7.38 (m, 30H); ^{13}C-NMR (100 MHz, $CDCl_3$) δ: 14.1 (2C), 22.7 (2C),

25.2, 25.5, 25.6, 26.1, 26.8, 26.9, 27.3, 28.6, 29.3, 29.4, 29.5 (5C), 29.6 (3C), 29.7 (7C), 29.8, 31.9 (2C), 33.0, 33.3, 35.3, 36.6, 36.9, 47.7, 50.0, 50.3, 69.3, 70.0, 71.7, 72.9, 73.4, 73.6, 74.7 (2C), 77.2, 78.6, 78.9, 80.1, 90.6, 99.9, 127.4 (2C), 127.5 (3C), 127.7 (2C), 127.8 (7C), 127.9 (2C), 128.2 (2C), 128.3 (8C), 128.4 (4C), 137.5, 138.4 (2C), 138.6 (2C), 138.7, 172.8 (2C); HRMS (ESI-QTOF) calcd for $C_{92}H_{134}N_2NaO_{10}$ $[M + Na]^+$ 1449.9931, found 1449.9927.

Synthesis of Compound 11b.

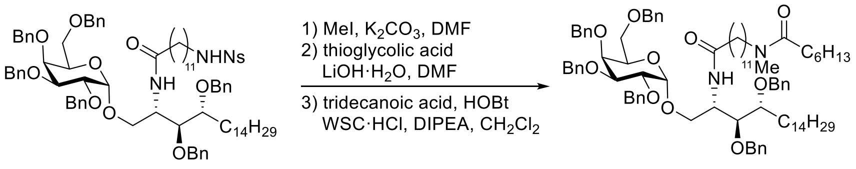

10 **11b**

By a procedure identical with that described for synthesis of **11a** from **10**, the amide **10** (17.8 mg, 0.0127 mmol) was converted into **11b** as colorless oil (6.8 mg, 40% yield): ^{1}H-NMR (400 MHz, $CDCl_3$) δ: 0.86-0.90 (m, 6H), 1.22-1.30 (m, 44H), 1.44-1.55 (m, 6H), 1.61-1.65 (m, 2H), 1.88-1.97 (m, 2H), 2.26-2.30 (m, 2H), 2.90-2.95 (m, 3H), 3.20-3.24 (m, 1H), 3.31-3.35 (m, 1H), 3.39-3.43 (m, 1H), 3.47-3.51 (m, 2H), 3.73 (dd, J = 11.0, 3.6 Hz, 1H), 3.84-3.88 (m, 2H), 3.89-3.94 (m, 2H), 3.99-4.06 (m, 2H), 4.13-4.19 (m, 1H), 4.36 (d, J = 11.9 Hz, 1H), 4.42 (d, J = 11.9 Hz, 1H), 4.45-4.59 (m, 4H), 4.64 (d, J = 11.9 Hz, 1H), 4.71-4.79 (m, 4H), 4.84 (d, J = 3.6 Hz, 1H), 4.91 (d, J = 11.7 Hz, 1H), 6.13-6.16 (m, 1H), 7.21-7.38 (m, 30H); ^{13}C-NMR (100 MHz, $CDCl_3$) δ: 14.0, 14.1, 22.5, 22.7, 25.1, 25.5, 25.6, 26.0, 26.8, 26.9, 28.5, 29.1, 29.2, 29.3, 29.4 (2C), 29.5 (2C), 29.6 (4C), 29.7, 29.8, 31.6, 31.9, 33.0, 33.3, 33.7, 36.6 (2C), 47.6, 50.0, 50.3, 69.2, 69.9, 71.6, 72.8, 73.5, 73.6, 74.7 (2C), 77.2, 78.6, 78.8, 80.1, 90.6, 99.5, 127.4 (2C), 127.5 (3C), 127.6, 127.7, 127.8 (7C), 127.9 (2C), 128.2 (4C), 128.3 (8C), 128.4 (2C), 137.5, 138.3, 138.4, 138.6 (2C), 138.7, 172.8, 173.0; HRMS (ESI-QTOF) calcd for $C_{86}H_{122}N_2NaO_{10}$ $[M + Na]^+$ 1365.8992, found 1365.8993.

Synthesis of Compound 4a.

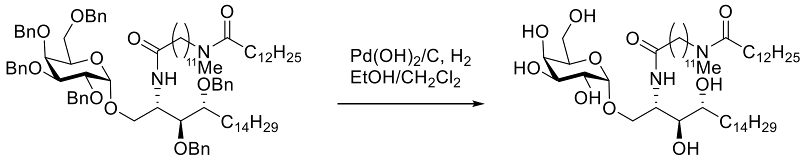

11a **4a**

To a stirred solution of **11a** (2.80 mg, 1.96 μmol) in ethanol/CH_2Cl_2 = 3/1 (1.0 mL) was added $Pd(OH)_2/C$ (3.00 mg, 0.0214 mmol). After being stirred overnight under H_2 (0.8 MPa), the reaction mixture was filtered off through a Celite pad. The filtrate was concentrated under reduced pressure to afford **4a** as white solid (1.7 mg, quant.): ^{1}H-NMR (400 MHz, $CDCl_3$:CD_3OD = 10:1) δ: 0.88 (t, J = 6.6 Hz, 6H), 1.26-1.40 (m, 56H), 1.48-1.69 (m, 8H), 2.18-2.22 (m, 2H), 2.28-2.32 (m, 2H), 2.90-2.99 (m, 3H), 3.23-3.44 (m, 2H), 3.50-3.57 (m, 2H), 3.67-3.74 (m, 3H), 3.76-3.82 (m, 3H), 3.89 (dd, J = 10.7, 4.4 Hz, 1H), 3.94-3.96 (m, 1H), 4.17-4.22 (m, 1H), 4.91 (d, J = 3.9 Hz,

1H), 7.17-7.20 (m, 1H); ^{13}C-NMR (100 MHz, $CDCl_3$:CD_3OD = 10:1) δ: 13.8 (2C), 22.4, 22.5, 25.0, 25.4, 25.6, 25.7, 26.6 (2C), 27.0, 28.3, 29.1 (2C), 29.2 (3C), 29.3 (5C), 29.4, 29.5 (4C), 29.6, 31.4, 31.7 (2C), 32.5, 32.9, 33.3, 33.6, 35.4, 36.3, 47.7, 50.1, 61.8, 67.3, 68.7, 69.6, 70.0, 70.4, 71.9, 74.6, 99.5, 173.7, 173.8; HRMS (ESI-QTOF) calcd for $C_{50}H_{98}N_2NaO_{10}$ [M + Na]$^+$ 909.7114, found 909.7122.

Synthesis of Compound 4b.

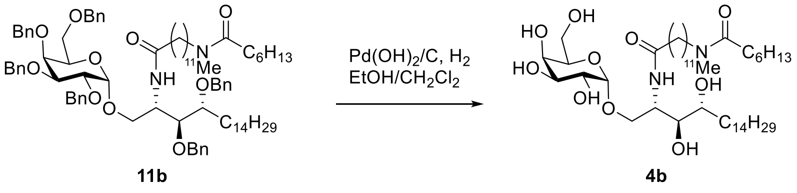

By a procedure identical with that described for synthesis of **4a** from **11a**, the amide **11b** (6.00 mg, 4.46 μmol) was converted into **4b** as white solid (3.6 mg, quant.): ^{1}H-NMR (400 MHz, $CDCl_3$:CD_3OD = 10:1) δ: 0.86-0.90 (m, 6H), 1.26-1.30 (m, 44H), 1.48-1.69 (m, 8H), 2.18-2.22 (m, 2H), 2.28-2.33 (m, 2H), 2.90-2.99 (m, 3H), 3.25-3.42 (m, 2H), 3.51-3.57 (m, 2H), 3.67-3.74 (m, 3H), 3.77-3.82 (m, 3H), 3.89 (dd, J = 10.8, 4.5 Hz, 1H), 3.94-3.97 (m, 1H), 4.16-4.22 (m, 1H), 4.92 (d, J = 3.1 Hz, 1H), 7.17-7.21 (m, 1H); ^{13}C-NMR (100 MHz, $CDCl_3$:CD_3OD = 10:1) δ: 13.8, 13.9, 22.4, 22.5, 25.0, 25.4, 25.6, 25.7, 26.5, 26.6, 27.0, 28.3, 28.9, 29.0, 29.1, 29.2 (2C), 29.3 (2C), 29.4, 29.5 (2C), 29.6, 31.4, 31.7, 32.6, 32.9, 33.3, 33.6, 35.4, 36.3, 47.7, 50.1, 61.8, 67.4, 68.7, 69.6, 70.1, 70.4, 71.9, 74.7, 99.5, 173.6, 173.7; HRMS (ESI-QTOF) calcd for $C_{44}H_{86}N_2NaO_{10}$ [M + Na]$^+$ 825.6175, found 825.6176.

Synthesis of Compound 5a.

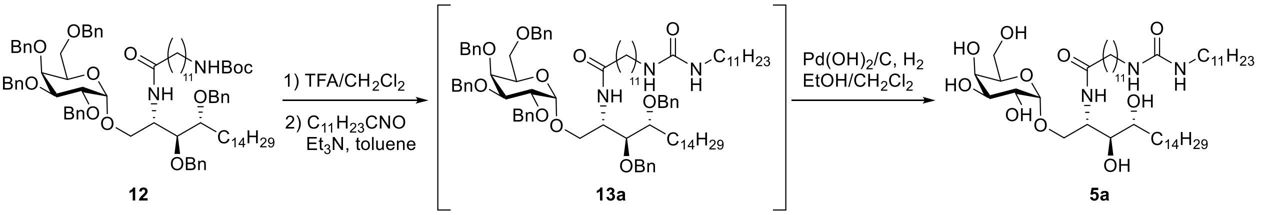

A mixture of **12** (20.0 mg, 0.0152 mmol) in TFA/ CH_2Cl_2 = 1/1 (110 μL) was stirred at room temperature. After being stirred for 7 h, the mixture was diluted with CH_2Cl_2. The whole was washed with saturated aqueous $NaHCO_3$, H_2O and brine, dried over Na_2SO_4, and concentrated under reduced pressure to give a crude amine. The amine was dissolved in toluene (205 μL). Isocyanate (0.0934 mmol) in toluene (150 μL) and Et_3N (15.0 μL, 0.107 mmol) was added to the stirred mixture. After being stirred overnight at room temperature, the mixture was concentrated under reduced pressure to give an oily residue. The residue was purified by column chromatography over silica gel with *n*-hexane-EtOAc (gradient 3:1 to 2:1) to give **13a** as white solid (5.7 mg), which was used without further purification. To a stirred solution of **13a** (5.1 mg) in ethanol/CH_2Cl_2 = 3/1 (1.0 mL) was added $Pd(OH)_2$/C (3.0 mg, 0.0214 mmol). After being stirred overnight under H_2 (0.8 MPa), the reaction mixture was filtered off through a Celite pad. The filtrate was concentrated under reduced pressure to afford **5a** as white solid

(3.2 mg, 27%): ^{1}H-NMR (400 MHz, $CDCl_3$:CD_3OD = 10:1) δ: 0.88 (t, *J* = 6.3 Hz, 6H), 1.26-1.28 (m, 50H), 1.42-1.51 (m, 6H), 1.57-1.66 (m, 6H), 2.19-2.22 (m, 2H), 3.10 (t, *J* = 6.6 Hz, 4H), 3.41-3.45 (m, 3H), 3.52-3.54 (m, 1H), 3.68-3.79 (m, 4H), 3.87-3.91 (m, 1H), 3.96-3.98 (m, 1H), 4.18-4.20 (m, 1H), 4.91-4.92 (m, 1H); ^{13}C NMR (100 MHz, $CDCl_3$:CD_3OD = 10:1) δ: 13.7 (2C), 22.4, 25.5, 26.5, 26.6, 29.0 (3C), 29.1 (6C), 29.2 (2C), 29.3 (4C), 29.4 (7C), 29.5, 29.8, 29.9, 31.6, 32.4, 36.2, 39.9 (2C), 50.1, 61.6, 67.1, 68.6, 69.5, 70.0, 70.4, 71.7, 74.5, 99.4, 159.3, 174.2; HRMS (ESI-QTOF) calcd for $C_{48}H_{95}N_3NaO_{10}$ $[M + Na]^+$ 896.6910, found 896.6905.

Synthesis of Compound 5b.

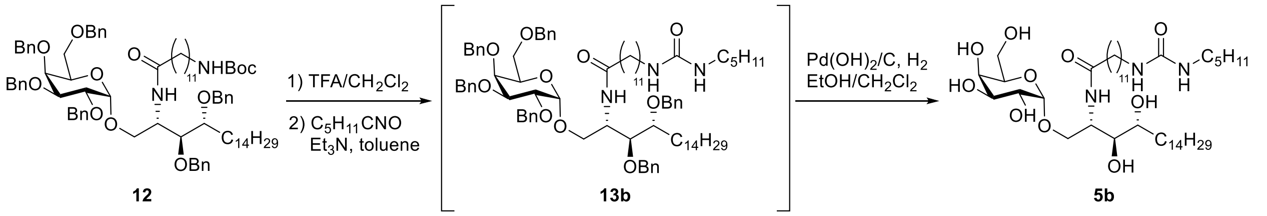

By a procedure identical with that described for synthesis of **13a** from **12**, the amide **12** (20.6 mg, 0.0156 mmol) was converted into **5b** as white solid (2.0 mg, 26%): ^{1}H-NMR (400 MHz, $CDCl_3$:CD_3OD = 10:1) δ: 0.86-0.92 (m, 6H), 1.26-1.34 (m, 40H), 1.45-1.49 (m, 4H), 1.54-1.65 (m, 6H), 2.18-2.23 (m, 2H), 3.08-3.12 (m, 4H), 3.20-3.26 (m, 1H), 3.37-3.38 (m, 2H), 3.53-3.55 (m, 1H), 3.58-3.62 (m, 2H), 3.77-3.78 (m, 2H), 3.86-3.90 (m, 1H), 3.95-3.97 (m, 1H), 4.18-4.19 (m, 1H), 4.91-4.92 (m, 1H); ^{13}C NMR (100 MHz, $CDCl_3$:CD_3OD = 10:1) δ: 13.7, 13.9, 22.2, 22.5, 25.6, 25.7, 26.6, 28.8, 29.0 (2C), 29.1, 29.2 (3C), 29.5 (8C), 29.6 (2C), 29.8, 31.7, 32.6, 36.3, 40.0 (2C), 50.1, 61.8, 67.3, 68.7, 69.6, 70.0, 70.4, 71.9, 74.6, 99.5, 159.2, 174.2; HRMS (ESI-QTOF) calcd for $C_{42}H_{83}N_3NaO_{10}$ $[M + Na]^+$ 812.5971, found 812.5977.

Synthesis of Compound 14.

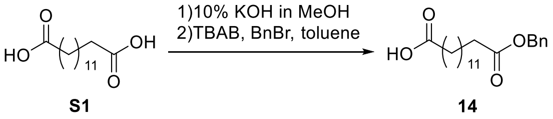

To a stirred solution of **S1** (200 mg, 0.774 mmol) in MeOH (6.0 mL) was added 10% KOH in MeOH (550 μL). After being stirred at room temperature for 20 min, the mixture was concentrated under reduced pressure. To a stirred solution of the residue in toluene (2.0 mL) were added TBAB (25 mg, 0.0775 mmol) and BnBr (101 μL, 0.850 mmol). After being stirred reflux for 6 h, the mixture was diluted with Et_2O. The whole was washed with aqueous 0.5 M HCl, dried over Na_2SO_4, and concentrated under reduced pressure to give an oily residue, which was purified by column chromatography over silica gel with *n*-hexane-EtOAc (gradient 4:1 to 3:1) to give **14** as white solid (83.7 mg, 31% yield). All the spectral data were in agreement with those reported by Gokel, G. et al. (*Synthesis* **2014,** *46*, 2771-2779): ^{1}H-NMR (400 MHz, $CDCl_3$) δ: 1.34-1.25 (m, 16H), 1.59-1.67 (m, 4H), 2.35 (t, *J* = 7.5 Hz, 2H), 2.35 (t, *J* = 7.5 Hz, 2H), 5.11 (s, 2H), 7.30-7.39 (m, 5H).

Synthesis of Compound 16a.

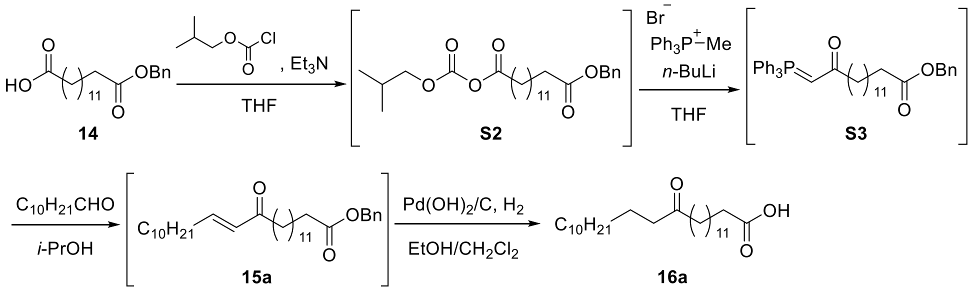

To a stirred solution of **14** (260 mg, 0.746 mmol) in THF (1.5 mL) were added Et_3N (261 μL, 1.87 mmol) and isobutyl chlorocarbonate (195 μL, 1.49 mmol) at 0 °C and stirred at room temperature for 2.5 h. The resulting solution of **S2** was used for the next reaction. To a stirred solution of methyltriphenylphosphonium bromide (813 mg, 2.28 mmol) in THF (3.7 mL) was added *n*-BuLi (1.35 mL, 2.21 mmol) at –60 °C. After being stirred overnight at –70 °C, the mixture was added to the above solution of **S2** at –70 °C. After being stirred at room temperature for 6 h, the mixture was diluted with EtOAc. The whole was washed with saturated aqueous NH_4Cl and brine, dried over Na_2SO_4, and concentrated under reduced pressure to give an oily residue, which was filtered by column chromatography over silica gel with EtOAc to give **S3** as yellow oil, which was used without further purification. To a stirred solution of **S3** (312 mg, 0.514 mmol) in 2-propanol (857 μL) was added undecanal (211 μL, 1.03 mmol). After being stirred overnight under reflux, the mixture was diluted with CH_2Cl_2. The whole was washed with aqueous $NaHCO_3$, H_2O and brine, dried over Na_2SO_4, and concentrated under reduced pressure to give an oily residue. The residue was purified by column chromatography over silica gel with *n*-hexane-EtOAc (13:1) to give **15a** as white solid (38.3 mg), which was used without further purification. To a stirred solution of **15a** (38.3 mg) in ethanol/CH_2Cl_2 = 3/1 (2 mL) was added $Pd(OH)_2/C$ (4.00 mg, 0.0285 mmol). After being stirred overnight under H_2, the reaction mixture was filtered off through a Celite pad. The filtrate was concentrated under reduced pressure to afford the crude **16a** as white solid (9.9 mg), which was used without further purification.

Synthesis of Compound 15b.

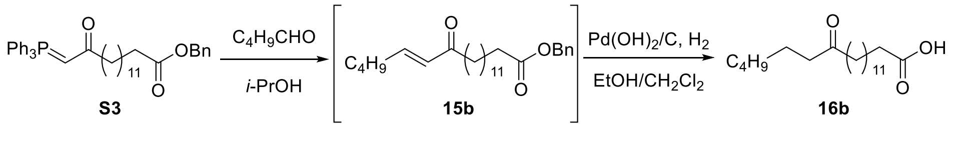

By a procedure identical with that described for synthesis of **16a** from **S3**, the benzyl ester **S3** (113 mg, 0.186 mmol) was converted into **16b** as white solid (34.8 mg), which was used without further purification.

Synthesis of Compound 18a.

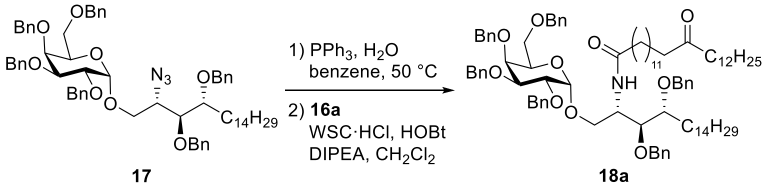

To a stirred solution of **17** (28.0 mg, 0.0268 mmol) in benzene (1.3 mL) and H_2O (52 μL) was added PPh_3 (17.5 mg, 0.0667 mmol). After being stirred overnight at 50 °C, the mixture was concentrated under reduced pressure, and azeotroped three times with toluene. The residue was dissolved in CH_2Cl_2 (1.0 mL). Carboxylic acid **16a** (9.90 mg, 0.0241 mmol), HOBt (40.2 mg, 0.297 mmol), WSC·HCl (57.0 mg, 0.297 mmol) and DIPEA (62.2 μL, 0.359 mmol) were added to the stirred mixture at 0 °C. After being stirred overnight at room temperature, the mixture was diluted with CH_2Cl_2. The whole was washed with saturated aqueous $NaHCO_3$, H_2O and brine, dried over Na_2SO_4, and concentrated under reduced pressure to give an oily residue, which was purified by column chromatography over silica gel with *n*-hexane-EtOAc (12:1) to give **18a** as colorless oil (17.4 mg, 51% yield): ^{1}H-NMR (400 MHz, $CDCl_3$) δ: 0.88 (t, *J* = 6.8 Hz, 6H), 1.20-1.31 (m, 56H), 1.43-1.68 (m, 10H), 1.86-1.99 (m, 2H), 2.35-2.37 (m, 4H), 3.40 (dd, *J* = 9.5, 6.1 Hz, 1H), 3.45-3.51 (m, 2H), 3.73 (dd, *J* = 11.0, 3.7 Hz, 1H), 3.85-3.94 (m, 4H), 4.01-4.06 (m, 2H), 4.12-4.19 (m, 1H), 4.36 (d, *J* = 11.7 Hz, 1H), 4.42 (d, *J* = 11.7 Hz, 1H), 4.45-4.61 (m, 4H), 4.64 (d, *J* = 11.7 Hz, 1H), 4.72-4.81 (m, 4H), 4.84 (d, *J* = 3.9 Hz, 1H), 4.92 (d, *J* = 11.2 Hz, 1H), 6.13 (d, *J* = 8.8 Hz, 1H), 7.19-7.38 (m, 30H); ^{13}C-NMR (100 MHz, $CDCl_3$) δ: 14.1 (2C), 22.7 (2C), 23.9, 25.7, 26.1, 29.3 (3C), 29.4 (3C), 29.5 (4C), 29.6 (5C), 29.7 (9C), 29.8 (2C), 31.9 (2C), 36.6, 42.8 (2C), 50.3, 69.3, 69.5, 70.0, 71.7, 72.9, 73.4, 73.6, 74.7, 74.8, 77.2, 78.7, 78.9, 80.1, 90.0, 99.6, 127.4 (2C), 127.5 (3C), 127.6, 127.7, 127.8 (3C), 127.9 (6C), 128.2 (2C), 128.3 (8C) 128.4 (4C), 137.6, 138.4 (2C), 138.6, 138.7 (2C), 172.8, 211.7; HRMS (ESI-QTOF) calcd for $C_{92}H_{133}NNaO_{10}$ [M + Na]$^+$ 1434.9822, found 1434.9813.

Synthesis of Compound 18b.

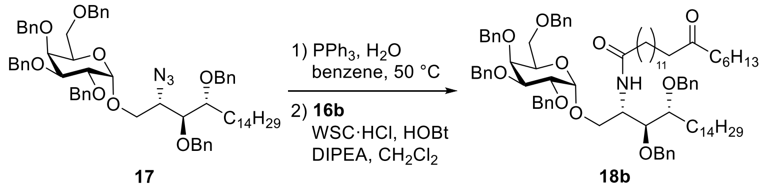

By a procedure identical with that described for synthesis of **18a** from **17**, the azide **17** (15.9 mg, 0.0152 mmol) was converted into **18b** as colorless oil (2.8 mg, 14% yield): ^{1}H-NMR (400 MHz, $CDCl_3$) δ: 0.88 (t, *J* = 6.6 Hz, 6H), 1.18-1.30 (m, 44H), 1.49-1.67 (m, 10H), 1.88-2.00 (m, 2H), 2.34-2.42 (m, 4H), 3.38-3.43 (m, 1H), 3.47-3.51 (m, 2H), 3.72 (dd, *J* = 10.5, 3.7 Hz, 1H), 3.84-3.93 (m, 4H), 4.00-4.06 (m, 2H), 4.11-4.18 (m, 1H), 4.36 (d, *J* = 11.7 Hz, 1H), 4.41 (d, *J* = 11.7 Hz, 1H), 4.45-4.59 (m, 4H), 4.64 (d, *J* = 11.7 Hz, 1H), 4.72-4.81 (m, 4H), 4.84 (d, *J* = 3.4 Hz, 1H), 4.91 (d, *J* = 11.7 Hz, 1H), 6.17 (d, *J* = 8.8 Hz, 1H), 7.23-7.38 (m, 30H); ^{13}C-NMR (100 MHz, $CDCl_3$) δ: 14.0, 14.1, 22.5, 22.7, 23.9 (2C), 26.1, 28.9, 29.3, 29.4 (3C), 29.5 (3C), 29.6 (3C), 29.7 (9C), 29.9, 31.6, 31.9, 42.8 (2C), 52.9, 69.3, 69.5, 70.0, 71.7, 72.9, 73.4, 73.6, 74.7, 74.8, 77.2, 78.7, 78.9, 80.2, 89.7, 99.6, 127.4 (2C), 127.5 (2C), 127.6 (2C), 127.7, 127.8 (7C), 127.9 (2C), 128.2 (2C), 128.3 (6C), 128.4 (6C), 137.6, 138.5

(2C), 138.6, 138.7 (2C), 172.8, 211.8; HRMS (ESI-QTOF) calcd for $C_{86}H_{121}NNaO_{10}$ [M + Na]$^+$ 1350.8883, found 1350.8876.

Synthesis of Compound 7a.

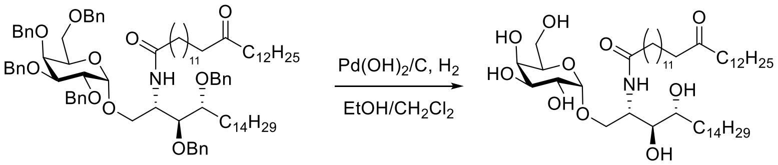

18a **7a**

By a procedure identical with that described for synthesis of **4a** from **11a**, the amide **18a** (9.10 mg, 6.44 μmol) was converted into **7a** as white solid (3.2 mg, 57%): ^{1}H-NMR (400 MHz, $CDCl_3$:CD_3OD = 10:1) δ: 0.88 (t, J = 6.6 Hz, 6H), 1.25-1.29 (m, 62H), 1.53-1.65 (m, 6H), 2.20 (t, J = 7.6 Hz, 2H), 2.40 (t, J = 7.6 Hz, 2H), 3.37-3.39 (m, 1H), 3.53-3.55 (m, 2H), 3.68-3.79 (m, 5H), 3.88 (dd, J = 9.9, 3.1 Hz, 1H), 3.95-3.96 (m, 1H), 4.16-4.19 (m, 1H), 4.91-4.92 (m, 1H), 7.25 (d, J = 8.5 Hz, 1H); ^{13}C-NMR (100 MHz, $CDCl_3$:CD_3OD = 10:1) δ: 13.9 (2C), 22.5 (2C), 23.7 (2C), 25.7, 29.1 (2C), 29.2 (4C), 29.3 (4C), 29.4 (3C), 29.5 (5C), 29.6 (7C), 31.7, 31.8, 32.4, 36.4, 42.7 (2C), 50.2, 61.8, 67.4, 68.7, 69.6, 70.1, 70.4, 71.9, 74.6, 99.5, 174.3, 212.9; HRMS (ESI-QTOF) calcd for $C_{50}H_{97}NNaO_{10}$ [M + Na]$^+$ 894.7005, found 894.6996.

Synthesis of Compound 7b.

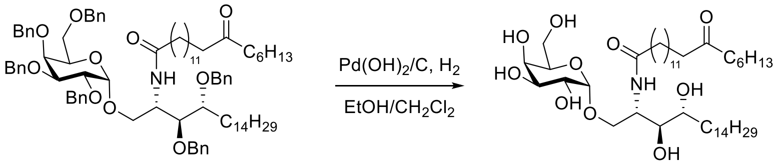

18b **7b**

By a procedure identical with that described for synthesis of **4a** from **11a**, the amide **18b** (2.40 mg, 1.81 μmol) was converted into **7b** as white solid (1.4 mg, quant.): ^{1}H-NMR (400 MHz, $CDCl_3$:CD_3OD = 10:1) δ: 0.88 (t, J = 6.8 Hz, 6H), 1.17-1.26 (m, 48H), 1.41-1.45 (m, 2H), 1.52-1.66 (m, 6H), 2.19 (t, J = 7.6 Hz, 2H), 2.40 (t, J = 7.6 Hz, 2H), 3.21-3.22 (m, 2H), 3.37-3.41 (m, 1H), 3.47-3.55 (m, 2H), 3.67-3.78 (m, 3H), 3.89 (dd, J = 10.5, 4.1 Hz, 1H), 3.94-3.97 (m, 1H), 4.18-4.19 (m, 1H), 4.91 (d, J = 2.0 Hz, 1H); ^{13}C-NMR (100 MHz, $CDCl_3$:CD_3OD = 10:1) δ: 14.1, 14.2, 22.6, 22.8, 24.0 (2C), 26.0, 29.0, 29.4, 29.5 (4C), 29.6 (2C), 29.7, 29.8 (9C), 29.9, 31.7, 32.1, 32.8, 36.6, 43.0 (2C), 50.4, 62.1, 67.6, 69.0, 69.9, 70.4, 70.7, 72.2, 74.9, 99.8, 174.6, 213.3; HRMS (ESI-QTOF) calcd for $C_{44}H_{85}NNaO_{10}$ [M + Na]$^+$ 810.6066, found 810.6068.

Synthesis of Compound S4.

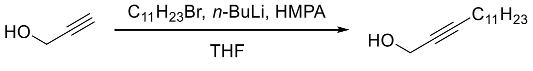

19 **S4**

To a stirred solution of propargylalcohol **19** (590 μL, 10.0 mmol) in THF (15 mL) and HMPA (4.2 mL) was added

n-BuLi (1.55 M solution in hexane; 12.9 mL, 20.0 mmol) at –78 °C. After the mixture was stirred for 30 min at –30 °C, 1-bromoundecane (2.44 mL, 11.0 mmol) was added. After being stirred overnight at room temperature, the mixture was diluted with EtOAc. The whole was washed with H_2O, dried over Na_2SO_4, and concentrated under reduced pressure to give an oily residue, which was purified by column chromatography over silica gel with *n*-hexane-EtOAc (4:1) to give **S4** as white solid (1.27 g, 60% yield): ^{1}H-NMR (400 MHz, $CDCl_3$) δ: 0.88 (t, *J* = 6.8 Hz, 3H), 1.26-1.30 (m, 16H), 1.37 (t, *J* = 7.1 Hz, 2H), 1.72 (t, *J* = 5.1 Hz, 1H), 2.19-2.23 (m, 2H), 4.25 (t, *J* = 2.2 Hz, 2H); ^{13}C-NMR (100 MHz, $CDCl_3$) δ: 14.1, 18.7, 22.7, 28.6, 28.9, 29.1, 29.3, 29.5, 29.6 (2C), 31.9, 51.4, 78.2, 86.6; HRMS (ESI-QTOF) calcd for $C_{14}H_{26}NaO$ $[M + Na]^+$ 233.1876, found 233.1875.

Synthesis of Compound S5.

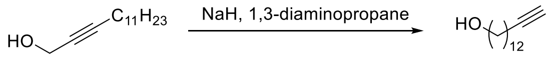

S4 S5

The mixture of 1, 3-diaminopropane (14.6 mL) and NaH (1.17 g, 29.3 mmol) was stirred for 30 min at 0 °C, warmed to 70 °C, and stirred for 1 h. **S4** (1.23 g, 5.85 mmol) was added to the stirred mixture at room temperature. After being stirred overnight at 55 °C, the mixture was diluted with EtOAc. The whole was washed with H_2O, dried over Na_2SO_4, and concentrated under reduced pressure to give an oily residue, which was purified by column chromatography over silica gel with *n*-hexane-EtOAc (4:1) to give **S5** as white solid (0.91 g, 74% yield): ^{1}H-NMR (400 MHz, $CDCl_3$) δ: 1.27-1.40 (m, 16H), 1.60-1.49 (m, 4H), 1.94 (t, *J* = 2.6 Hz, 1H), 2.18 (td, *J* = 7.1, 2.6 Hz, 2H), 3.64 (t, *J* = 5.6 Hz, 2H); ^{13}C-NMR (100 MHz, $CDCl_3$) δ: 18.4, 25.7, 28.5, 28.7, 29.1, 29.4 (2C), 29.5 (2C), 29.6, 32.8, 63.1, 68.0, 84.8; HRMS (ESI-QTOF) calcd for $C_{14}H_{26}NaO$ $[M + Na]^+$ 233.1876, found 233.1870.

Synthesis of Compound S6.

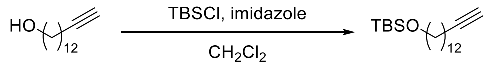

S5 S6

To a stirred solution of **S5** (858 mg, 4.08 mmol) in CH_2Cl_2 (13.6 mL) were added imidazole (555 mg, 8.15 mmol) and TBSCl (738 mg, 4.90 mmol) at 0 °C. After being stirred overnight at room temperature, the mixture was diluted with CH_2Cl_2. The whole was washed with brine, dried over Na_2SO_4, and concentrated under reduced pressure to give an oily residue, which was purified by column chromatography over silica gel with *n*-hexane-EtOAc (50:1) to give **S6** as white solid (1.25 g, 95% yield): ^{1}H-NMR (400 MHz, $CDCl_3$) δ: 0.05 (s, 6H), 0.89-0.89 (m, 9H), 1.27-1.32 (m, 14H), 1.37-1.42 (m, 2H), 1.47-1.54 (m, 4H), 1.94 (t, *J* = 2.7 Hz, 1H), 2.18 (td, *J* = 7.1, 2.7 Hz, 2H), 3.60 (t, *J* = 6.6 Hz, 2H); ^{13}C-NMR (100 MHz, $CDCl_3$) δ: –5.3 (2C), 18.4, 25.8, 26.0 (3C), 28.5, 28.8, 29.1, 29.4, 29.5, 29.6 (4C), 32.9, 63.3, 68.0, 84.9; HRMS (ESI-QTOF) calcd for $C_{20}H_{40}NaOSi$ $[M + Na]^+$ 347.2741, found 347.2742.

Synthesis of Compound 20a.

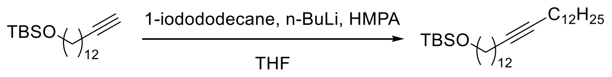

To a stirred solution of **S6** (575 mg, 1.77 mmol) in THF (2.23 mL) and HMPA (619 μL) was added *n*-BuLi (1.55 M solution in hexane; 1.26 mL, 1.95 mmol) at –65 °C. After being stirred for 10 min at –10 °C, the stirred mixture was added 1-iodododecane (525 μL, 2.13 mmol) in THF (619 μL). After being stirred overnight at room temperature, the mixture was diluted with EtOAc. The whole was washed with H_2O, dried over Na_2SO_4, and concentrated under reduced pressure to give an oily residue, which was purified by column chromatography over silica gel with *n*-hexane-EtOAc (70:1) to give **20a** as white solid (650 mg, 74% yield): ^{1}H-NMR (400 MHz, $CDCl_3$) δ: 0.05 (s, 6H), 0.88-0.93 (m, 12H), 1.32-1.26 (m, 30H), 1.34-1.38 (m, 4H), 1.43-1.52 (m, 6H), 2.12-2.15 (m, 4H), 3.60 (t, *J* = 6.6 Hz, 2H); ^{13}C-NMR (100 MHz, $CDCl_3$) δ: –5.3 (2C), 14.1, 18.8 (2C), 22.7, 25.8, 26.0 (3C), 28.9 (2C), 29.2 (5C), 29.4, 29.5, 29.6 (4C), 29.7 (4C), 31.9, 32.9, 63.3, 80.2 (2C); HRMS (ESI-QTOF) calcd for $C_{32}H_{64}NaOSi$ $[M + Na]^+$ 515.4619, found 515.4617.

Synthesis of Compound 20b.

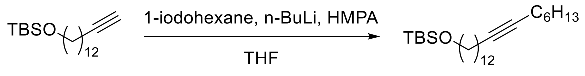

By a procedure identical with that described for synthesis of **20a** from **S6**, the alkyne **S6** (464 mg, 1.43 mmol) was converted into **20b** as white solid (480 mg, 82% yield): ^{1}H-NMR (400 MHz, $CDCl_3$) δ: 0.05 (s, 6H), 0.93-0.87 (m, 12H), 1.26-1.31 (m, 18H), 1.33-1.40 (m, 4H), 1.43-1.52 (m, 6H), 2.12-2.15 (m, 4H), 3.59 (t, *J* = 6.6 Hz, 2H); ^{13}C-NMR (100 MHz, $CDCl_3$) δ: –5.3 (2C), 14.1, 18.4, 18.8 (2C), 22.6, 25.8, 26.0 (3C), 26.1, 28.5, 28.9, 29.1, 29.2 (2C), 29.5, 29.6 (3C), 31.4, 32.9, 63.3, 80.2 (2C); HRMS (ESI-QTOF) calcd for $C_{26}H_{52}NaOSi$ $[M + Na]^+$ 431.3680, found 431.3678.

Synthesis of Compound S7.

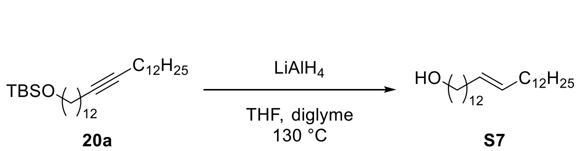

To a stirred solution of $LiAlH_4$ (158 mg, 4.2 mmol) in diglyme (757 μL) and THF (118 μL) was added **20a** (51.2 mg, 0.104 mmol) in diglyme (164 μL) at –65 °C. After being stirred overnight at 130 °C, the mixture was diluted with EtOAc. The whole was washed with 1M HCl, H_2O, brine, aqueous $NaHCO_3$ and brine, dried over Na_2SO_4, and concentrated under reduced pressure to give an oily residue, which was purified by column chromatography over silica gel with *n*-hexane-EtOAc (5:1) to give **S7** as white solid (28.7 mg, 73% yield): ^{1}H-NMR (400 MHz, $CDCl_3$) δ: 0.88 (t, *J* = 6.7 Hz, 3H), 1.31-1.26 (m, 38H), 1.53-1.60 (m, 2H), 1.95 (t, *J* = 5.4 Hz, 2H), 1.97 (t, *J* = 5.4 Hz, 2H), 3.64 (t, *J* = 6.7 Hz, 2H), 5.37-5.40 (m, 2H); ^{13}C-NMR (100 MHz, $CDCl_3$) δ: 14.1, 22.7, 25.7, 27.2, 29.2,

29.3, 29.4 (2C), 29.5, 29.6 (4C), 29.7 (6C), 29.8, 31.9, 32.6, 32.8, 63.1, 130.3 (2C); HRMS (ESI-QTOF) calcd for $C_{26}H_{52}NaO$ $[M + Na]^+$ 403.3910, found 403.3913.

Synthesis of Compound 21a.

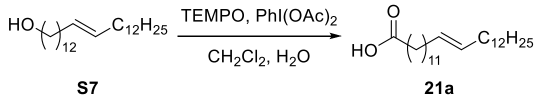

To a stirred solution of **S7** (5.60 mg, 0.0147 mmol) in CH_2Cl_2 (121 μL) and H_2O (61 μL) were added TEMPO (3.20 mg, 0.0205 mmol) and $PhI(OAc)_2$ (82.9 mg, 0.257 mmol). After being stirred for 7 h at room temperature, the mixture was diluted with CH_2Cl_2. The whole was washed with aqueous $Na_2S_2O_3$ and brine, dried over Na_2SO_4, and concentrated under reduced pressure to give an oily residue, which was purified by column chromatography over silica gel with *n*-hexane-EtOAc (4:1) to give **21a** as white solid (4.0 mg, 69% yield): ^{1}H-NMR (400 MHz, $CDCl_3$) δ: 0.88 (t, J = 6.8 Hz, 3H), 1.32-1.25 (m, 36H), 1.59-1.67 (m, 2H), 1.95 (t, J = 5.6 Hz, 2H), 1.97 (t, J = 5.6 Hz, 2H), 2.35 (t, J = 7.6 Hz, 2H), 5.37-5.39 (m, 2H); ^{13}C-NMR (100 MHz, $CDCl_3$) δ: 14.1, 22.7, 24.7, 29.1, 29.2 (3C), 29.4 (2C), 29.5 (3C), 29.6 (4C), 29.7 (4C), 31.9, 32.6, 33.8, 130.4 (2C), 178.8; HRMS (ESI-QTOF) calcd for $C_{26}H_{49}O_2$ $[M – H]^-$ 393.3738, found 393.3743.

Synthesis of Compound S8.

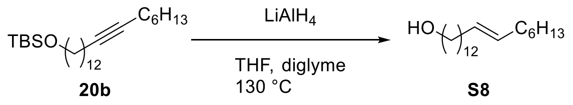

By a procedure identical with that described for synthesis of **S7** from **20a**, the alkyne **20b** (203 mg, 0.496 mmol) was converted into **S8** as white solid (102 mg, 70% yield): ^{1}H-NMR (400MHz, $CDCl_3$) δ: 0.90-0.86 (m, 3H), 1.26-1.34 (m, 26H), 1.52-1.60 (m, 2H), 1.94-1.99 (m, 4H), 3.62-3.67 (m, 2H), 5.34-5.43 (m, 2H); ^{13}C-NMR (100 MHz, $CDCl_3$) δ: 14.1, 22.7, 25.7, 28.8, 29.2, 29.4, 29.5, 29.6 (7C), 31.8, 32.6, 32.8, 63.1, 130.4 (2C); HRMS (ESI-QTOF) calcd for $C_{20}H_{40}NaO$ $[M + Na]^+$ 319.2971, found 319.2973.

Synthesis of Compound 21b.

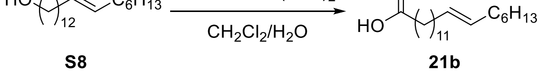

By a procedure identical with that described for synthesis of **21a** from **S7**, the alcohol **S8** (6.50 mg, 0.0219 mmol) was converted into **21b** as white solid (6.0 mg, 88% yield): ^{1}H-NMR (400 MHz, $CDCl_3$) δ: 0.89-0.86 (m, 3H), 1.26-1.32 (m, 24H), 1.59-1.67 (m, 2H), 1.96 (t, J = 5.8 Hz, 2H), 1.97 (t, J = 5.8 Hz, 2H), 2.35 (t, J = 7.5 Hz, 2H), 5.37-5.39 (m, 2H); ^{13}C-NMR (100 MHz, $CDCl_3$) δ: 14.1, 22.7, 24.7, 28.8, 29.1, 29.2 (2C), 29.4, 29.5, 29.6 (4C), 29.7, 31.8, 32.6, 33.9, 130.3, 130.4, 178.8; HRMS (ESI-QTOF) calcd for $C_{20}H_{37}O_2$ $[M – H]^-$ 309.2799, found

309.2805.

Synthesis of Compound S9.

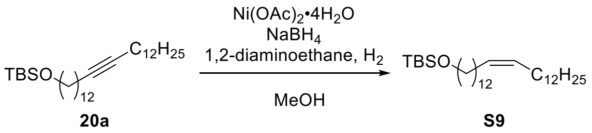

To a stirred solution of Ni(OAc)·4H_2O (38.2 mg, 0.153 mmol) in MeOH (791 μL) was added $NaBH_4$ (5.80 mg, 0.153 mmol). After the mixture was stirred for 5 min at room temperature, **20a** (302 mg, 0.613 mmol) was added. After being stirred overnight under H_2 (0.1 MPa) at room temperature, the reaction mixture was filtered off through a Celite pad. The mixture was diluted with CH_2Cl_2. The whole was washed with H_2O, dried over Na_2SO_4, and concentrated under reduced pressure to give an oily residue, which was purified by column chromatography over silica gel with *n*-hexane-EtOAc (100:1) to give **S9** as oil (0.25 g, 82% yield): ^{1}H-NMR (400 MHz, $CDCl_3$) δ: 0.05 (s, 6H), 0.86-0.90 (m, 12H), 1.26-1.34 (m, 38H), 1.47-1.52 (m, 2H), 2.00 (t, J = 6.1 Hz, 2H), 2.02 (t, J = 6.1 Hz, 2H), 3.60 (t, J = 6.7 Hz, 2H), 5.34-5.36 (m, 2H); ^{13}C-NMR (100 MHz, $CDCl_3$) δ: –5.3 (2C), 14.1, 18.4, 22.7, 25.8, 26.0 (3C), 27.2, 29.3 (2C), 29.4, 29.5, 29.6 (3C), 29.7 (7C), 29.8 (2C), 31.9, 32.9 (2C), 63.4, 129.9 (2C); HRMS (ESI-QTOF) calcd for $C_{32}H_{66}NaOSi$ $[M + Na]^+$ 517.4775, found 517.4779.

Synthesis of Compound 22a.

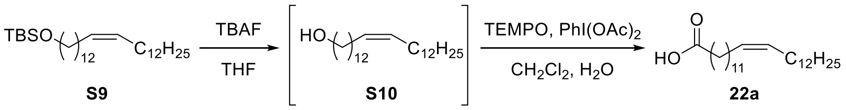

To a stirred solution of **S9** (50.6 mg, 0.102 mmol) in THF (511 μL) was added TBAF (307 μL, 0.307 mmol) at 0 °C. After being stirred for 5.5 h at room temperature, the mixture was diluted with EtOAc. The whole was washed with aqueous $NaHCO_3$, H_2O, and brine, dried over Na_2SO_4, and concentrated under reduced pressure to give an oily residue. The residue was purified by column chromatography over silica gel with *n*-hexane-EtOAc (5:1) to give **S10** as white solid (38.9 mg), which was used without further purification. By a procedure identical with that described for synthesis of **21a** from **S7**, the alcohol **S10** (16.3 mg) was converted into **22a** as white solid (7.8 mg, 46% yield): ^{1}H-NMR (400 MHz, $CDCl_3$) δ: 0.88 (t, J = 6.8 Hz, 3H), 1.34-1.26 (m, 36H), 1.59-1.67 (m, 2H), 2.00 (t, J = 6.1 Hz, 2H), 2.02 (t, J = 6.1 Hz, 2H), 2.35 (t, J = 7.5 Hz, 2H), 5.31-5.39 (m, 2H); ^{13}C-NMR (100 MHz, $CDCl_3$) δ: 14.1, 22.7, 24.7, 27.2 (2C), 29.1, 29.2, 29.3 (2C), 29.4 (2C), 29.5, 29.6 (3C), 29.7 (5C), 29.8, 31.9, 33.9, 129.9 (2C), 179.2; HRMS (ESI-QTOF) calcd for $C_{26}H_{49}O_2$ $[M – H]^-$ 393.3738, found 393.3743.

Synthesis of Compound S11.

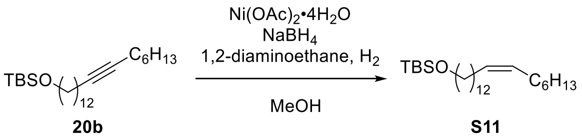

By a procedure identical with that described for synthesis of **S9** from **20a**, the alkyne **20b** (200 mg, 0.490 mmol) was converted into **S11** as oil (160 mg, 79% yield): ^{1}H-NMR (400 MHz, $CDCl_3$) δ: 0.05 (s, 6H), 0.87-0.90 (m, 12H), 1.34-1.26 (m, 26H), 1.47-1.54 (m, 2H), 2.01 (t, *J* = 6.2 Hz, 2H), 2.02 (t, *J* = 6.2 Hz, 2H), 3.60 (t, *J* = 6.6 Hz, 2H), 5.34-5.36 (m, 2H); ^{13}C-NMR (100 MHz, $CDCl_3$) δ: –5.3 (2C), 14.1, 18.4, 22.7, 25.8, 26.0 (3C), 27.2, 29.0, 29.3, 29.5, 29.6 (5C), 29.7, 29.8, 31.8, 32.9 (2C), 63.4, 129.9 (2C); HRMS (ESI-QTOF) calcd for $C_{26}H_{54}NaOSi$ $[M + Na]^+$ 433.3836, found 433.3833.

Synthesis of Compound 22b.

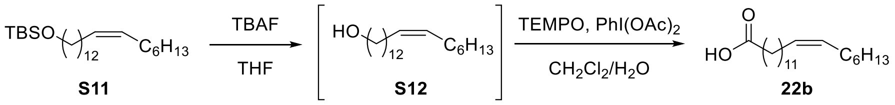

By a procedure identical with that described for synthesis of **S10** from **S9**, the alkene **S11** (106 mg, 0.257 mmol was converted into **S12** as white solid (65.3 mg), which was used without further purification. By a procedure identical with that described for synthesis of **21a** from **S7**, the alcohol **S12** (30.4 mg) was converted into **22b** as white solid (32.0 mg, 86%): ^{1}H-NMR (400 MHz, $CDCl_3$) δ: 0.88 (t, *J* = 6.6 Hz, 3H), 1.34-1.26 (m, 24H), 1.59-1.66 (m, 2H), 2.01 (t, *J* = 5.9 Hz, 2H), 2.02 (t, *J* = 5.9 Hz, 2H), 2.34 (t, *J* = 7.3 Hz, 2H), 5.31-5.39 (m, 2H); ^{13}C-NMR (100 MHz, $CDCl_3$) δ: 14.1, 22.7, 24.7, 27.2 (2C), 29.0, 29.1, 29.2, 29.3, 29.4, 29.5, 29.6 (2C), 29.7, 29.8, 31.8, 34.0, 129.9 (2C), 179.8; HRMS (ESI-QTOF) calcd for $C_{20}H_{37}O_2$ $[M – H]^-$ 309.2799, found 309.2795

Synthesis of Compound 8a.

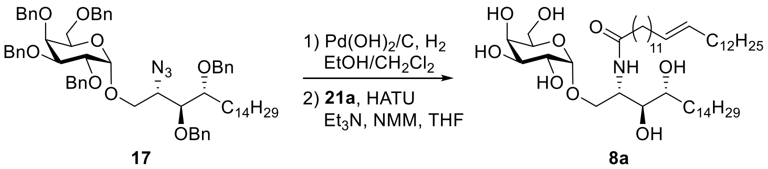

To a stirred solution of **17** (16.0 mg, 0.0153 mmol) in ethanol/CH_2Cl_2 = 3/1 (2.0 mL) was added $Pd(OH)_2$/C (16.0 mg, 0.114 mmol). After being stirred overnight under H_2 (0.8 MPa), the reaction mixture was filtered off through a Celite pad. The filtrate was concentrated under reduced pressure. The residue was dissolved in THF (91 μL). The carboxylic acid **21a** (5.30 mg, 0.0134 mmol) in THF (100 μL), HATU (5.80 mg, 0.0153 mmol), Et_3N (4.20 μL, 0.0303 mmol) and NMM (3.40 μL, 0.0309 mmol) were added to the stirred mixture. After being stirred overnight at room temperature, the mixture was concentrated under reduced pressure to give an oily residue, which was purified by column chromatography over silica gel with $CHCl_3$-MeOH (8:1) to give **8a** as white solid (4.1 mg, 31% yield): ^{1}H-NMR (400 MHz, $CDCl_3$:CD_3OD = 10:1) δ: 0.88 (t, *J* = 6.6 Hz, 6H), 1.22-1.32 (m, 60H), 1.52-1.69 (m, 4H), 1.96 (t, *J* = 5.4 Hz, 2H), 1.97 (t, *J* = 5.4 Hz, 2H), 2.20 (t, *J* = 7.6 Hz, 2H), 3.37-3.38 (m, 2H), 3.50-3.56 (m, 2H), 3.66-3.74 (m, 1H), 3.76-3.81 (m, 3H), 3.88 (dd, *J* = 10.7, 4.9 Hz, 1H), 3.95 (d, *J* = 3.4 Hz, 1H),

4.18-4.20 (m, 1H), 4.91 (d, *J* = 3.9 Hz, 1H), 5.38-5.40 (m, 2H), 7.23 (d, *J* = 8.8 Hz, 1H); ^{13}C-NMR (100 MHz, $CDCl_3$:CD_3OD = 10:1) δ: 13.9 (2C), 22.5 (2C), 25.7 (2C), 29.0 (2C), 29.2 (3C), 29.3 (2C), 29.4 (2C), 29.5 (16C), 29.6, 31.7 (2C), 32.4 (2C), 36.3, 50.2, 61.7, 67.3, 68.7, 69.6, 70.0, 70.5, 71.9, 74.6, 99.5, 130.2 (2C), 174.3; HRMS (ESI-QTOF) calcd for $C_{50}H_{97}NNaO_9$ $[M + Na]^+$ 878.7056, found 878.7058.

Synthesis of Compound 9a.

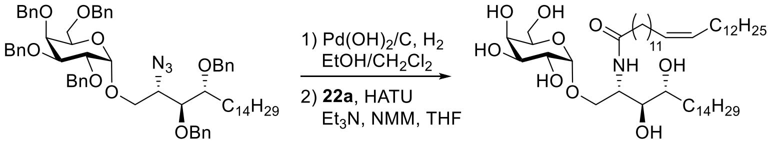

17 **9a**

By a procedure identical with that described for synthesis of **8a** from **17**, the azide **17** (16.0 mg, 0.0153 mmol) was converted into **9a** as white solid (3.4 mg, 26% yield): ^{1}H-NMR (400 MHz, $CDCl_3$:CD_3OD = 10:1) δ: 0.88 (t, *J* = 6.6 Hz, 6H), 1.26-1.31 (m, 60H), 1.55-1.60 (m, 4H), 2.01 (t, *J* = 5.9 Hz, 2H), 2.02 (t, *J* = 5.9 Hz, 2H), 2.20 (t, *J* = 7.8 Hz, 2H), 3.52-3.54 (m, 2H), 3.66-3.90 (m, 7H), 3.94 (d, *J* = 3.4 Hz, 1H), 4.18-4.22 (m, 1H), 4.91 (d, *J* = 3.9 Hz, 1H), 5.34-5.36 (m, 2H), 7.25 (d, *J* = 8.8 Hz, 1H); ^{13}C-NMR (100 MHz, $CDCl_3$:CD_3OD = 10:1) δ: 13.9 (2C), 22.5 (2C), 25.7, 27.0, 27.1, 29.1, 29.2 (3C), 29.3, 29.4 (2C), 29.5 (16C), 29.6 (3C), 31.8 (2C), 32.6, 36.4, 50.2, 61.8, 67.4, 68.8, 69.6, 70.1, 70.5, 72.0, 74.7, 99.5, 129.7, 129.8, 174.3; HRMS (ESI-QTOF) calcd for $C_{50}H_{97}NNaO_9$ $[M + Na]^+$ 878.7056, found 878.7064.

Synthesis of Compound 8b.

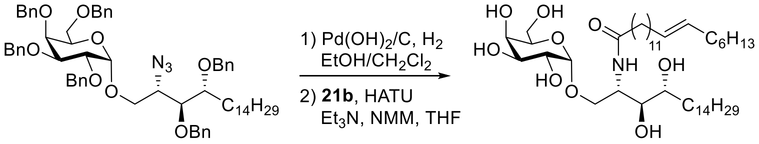

17 **8b**

By a procedure identical with that described for synthesis of **8a** from **17**, the azide **17** (14.8 mg, 0.0141 mmol) was converted into **8b** as white solid (2.6 mg, 24% yield): ^{1}H-NMR (400 MHz, $CDCl_3$:CD_3OD = 10:1) δ: 0.88 (t, *J* = 6.8 Hz, 6H), 1.26-1.33 (m, 48H), 1.54-1.69 (m, 4H), 1.96-1.97 (m, 4H), 2.20 (t, *J* = 7.6 Hz, 2H), 3.37-3.38 (m, 2H), 3.51-3.74 (m, 3H), 3.76-3.82 (m, 3H), 3.88 (dd, *J* = 10.7, 4.9 Hz, 1H), 3.95 (d, *J* = 2.9 Hz, 1H), 4.17-4.20 (m, 1H), 4.91 (d, *J* = 3.4 Hz, 1H), 5.38-5.40 (m, 2H), 7.22 (d, *J* = 8.3 Hz, 1H); ^{13}C-NMR (100 MHz, $CDCl_3$: CD_3OD = 10:1) δ: 13.9 (2C), 22.5 (2C), 25.7 (2C), 28.7, 29.1, 29.2, 29.3 (2C), 29.4 (2C), 29.5 (4C), 29.6 (7C), 29.7, 31.6, 31.8, 32.4, 32.5 (2C), 36.4, 50.2, 61.8, 67.5, 68.7, 69.7, 70.1, 70.5, 72.0, 74.6, 99.5, 130.2 (2C), 174.3; HRMS (ESI-QTOF) calcd for $C_{44}H_{85}NNaO_9$ $[M + Na]^+$ 794.6117, found 794.6110.

Synthesis of Compound 9b.

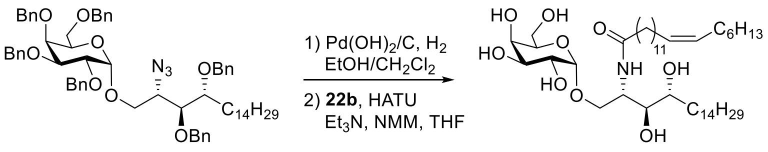

17 **9b**

By a procedure identical with that described for synthesis of **8a** from **17**, the azide **17** (15.0 mg, 0.0143 mmol) was converted into **9b** as white solid (5.5 mg, 50% yield): ^{1}H-NMR (400 MHz, $CDCl_3$:CD_3OD = 10:1) δ: 0.90-0.86 (m, 6H), 1.26-1.39 (m, 48H), 1.51-1.67 (m, 4H), 2.01 (t, J = 5.9 Hz, 2H), 2.02 (t, J = 5.9 Hz, 2H), 2.20 (t, J = 7.6 Hz, 2H), 3.51-3.56 (m, 2H), 3.67-3.83 (m, 6H), 3.87 (dd, J = 10.5, 4.6 Hz, 1H), 3.95 (d, J = 2.9 Hz, 1H), 4.15-4.18 (m, 1H), 4.91 (d, J = 3.4 Hz, 1H), 5.31-5.39 (m, 2H); ^{13}C-NMR (100 MHz, $CDCl_3$:CD_3OD = 10:1) δ: 13.9 (2C), 22.5 (2C), 25.7 (2C), 27.0 (2C), 28.8, 29.2 (3C), 29.3, 29.4, 29.5 (5C), 29.6 (8C), 31.6, 31.8, 32.3, 36.4, 50.2, 61.8, 67.4, 68.7, 69.7, 70.1, 70.5, 72.0, 74.5, 99.5, 129.7 (2C), 174.3; HRMS (ESI-QTOF) calcd for $C_{44}H_{85}NNaO_9$ $[M + Na]^+$ 794.6117, found 794.6124.

Water Solubility

The solubility of test compounds in Japanese Pharmacopoeia 2nd fluid for dissolution test (JP solution 2) was determined. Five microliter of the test compound (10 mmol/L solution in DMSO) was added to 495 μL of JP solution 2. The solution was stirred for 5 hours at room temperature and filtrated. The filtrate was diluted with acetonitrile and injected into the LC-MS/MS for analysis.

In Vitro Metabolic Stability in Liver Microsomes

The metabolic stability of test compounds at 1 μmol/L was determined in rat or human liver microsomes (Celsis In Vitro Technologies, Baltimore, USA, pooled donors). The reaction mixture containing 0.1 mol/L phosphate buffer, 0.5 mg/mL microsomes and NADPH-Co-factor was incubated at 37 °C. Then, 40 μL aliquots were taken at zero, 15 and 60 min and quenched using 160 μL of acetonitrile. The 50μL aliquot was diluted with 100 μL of 50% acetonitrile and filtrated. The filtrate was injected into the LC−MS/MS for analysis.

^{1}H NMR spectrum for compound 11a

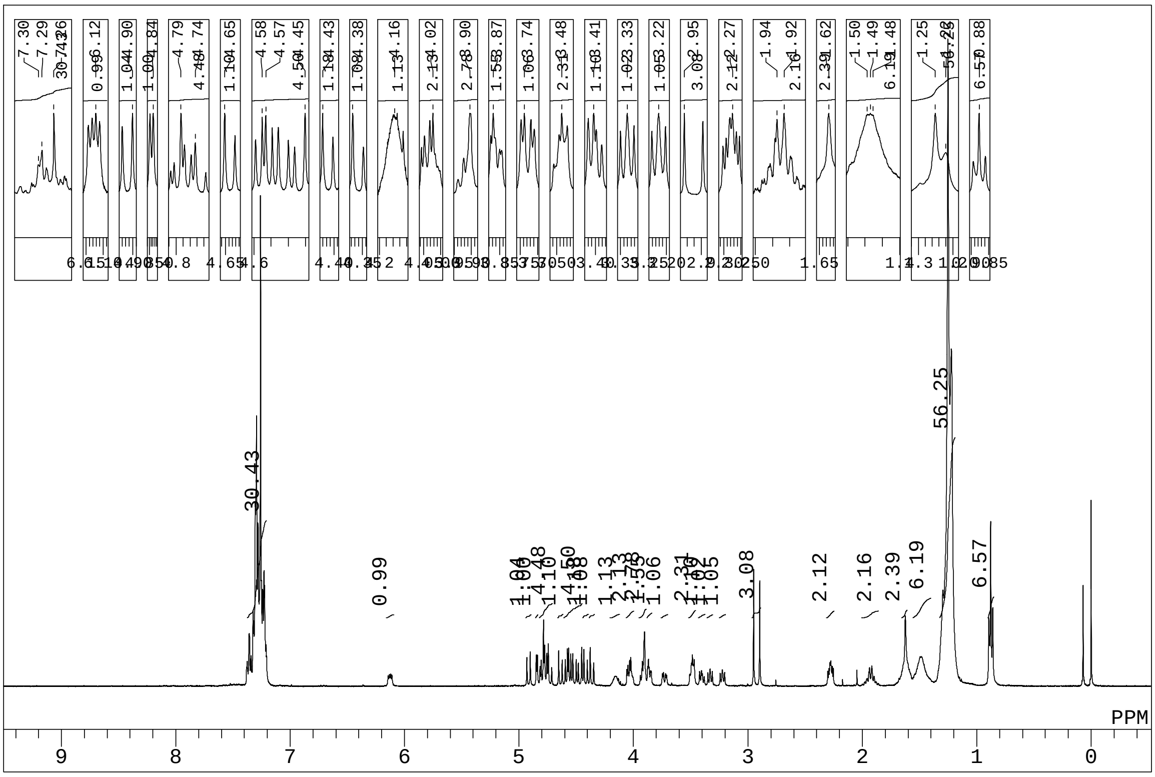

^{13}C NMR spectrum for compound 11a

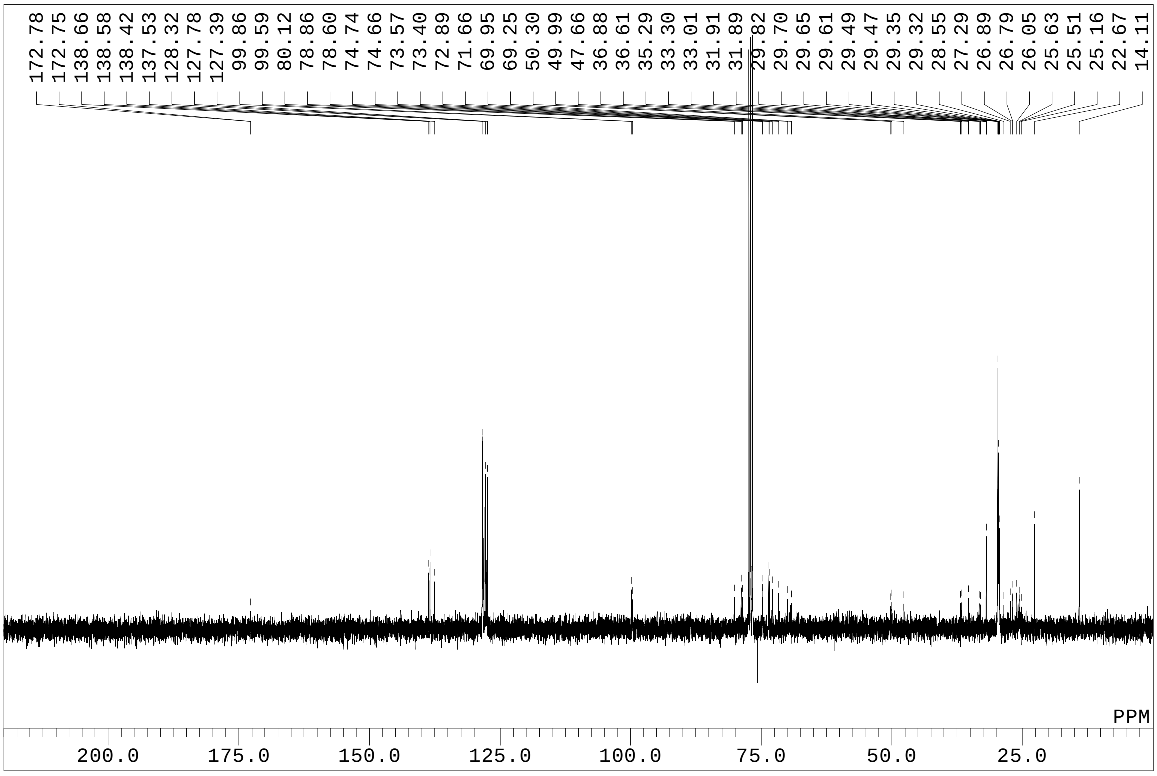

^{1}H NMR spectrum for compound 11b

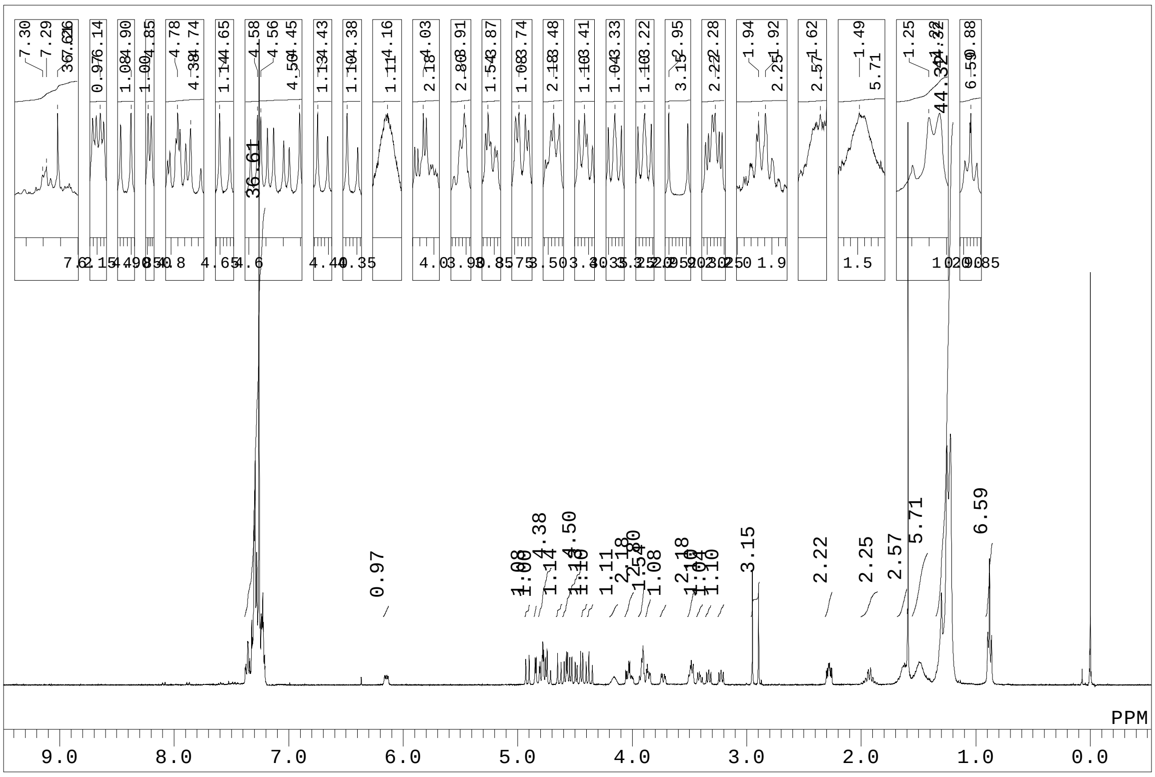

^{13}C NMR spectrum for compound 11b

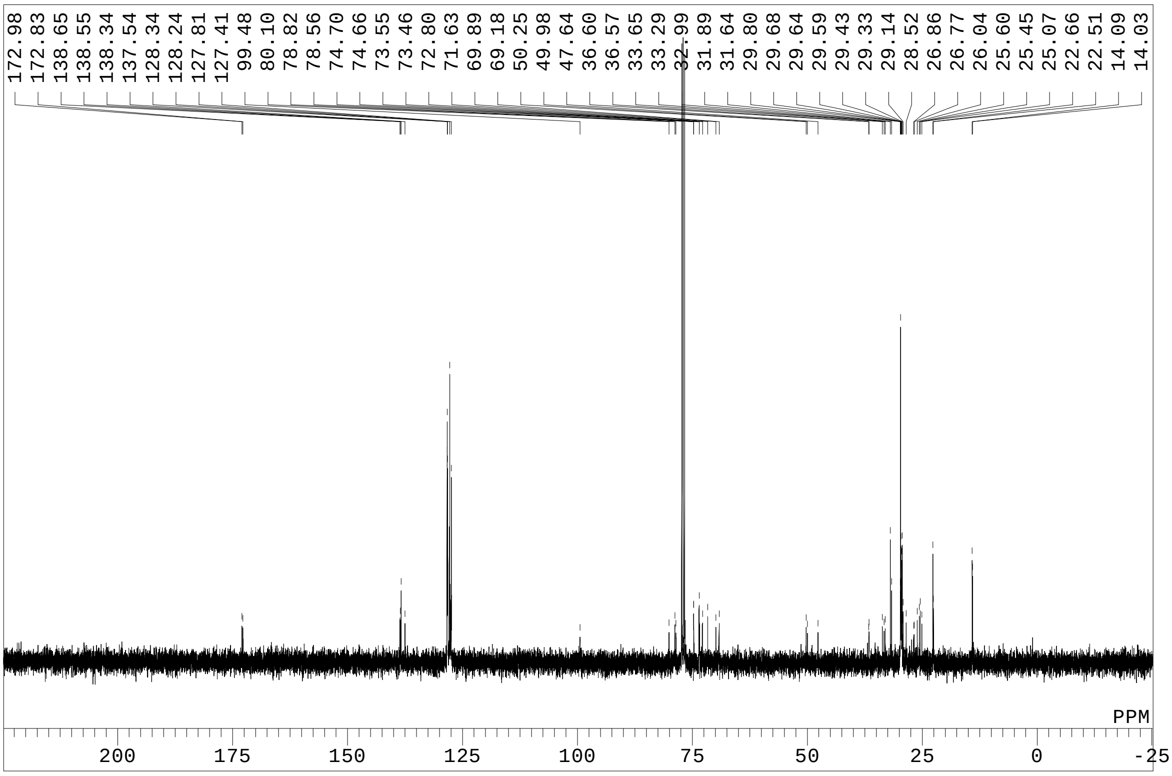

^{1}H NMR spectrum for compound 4a

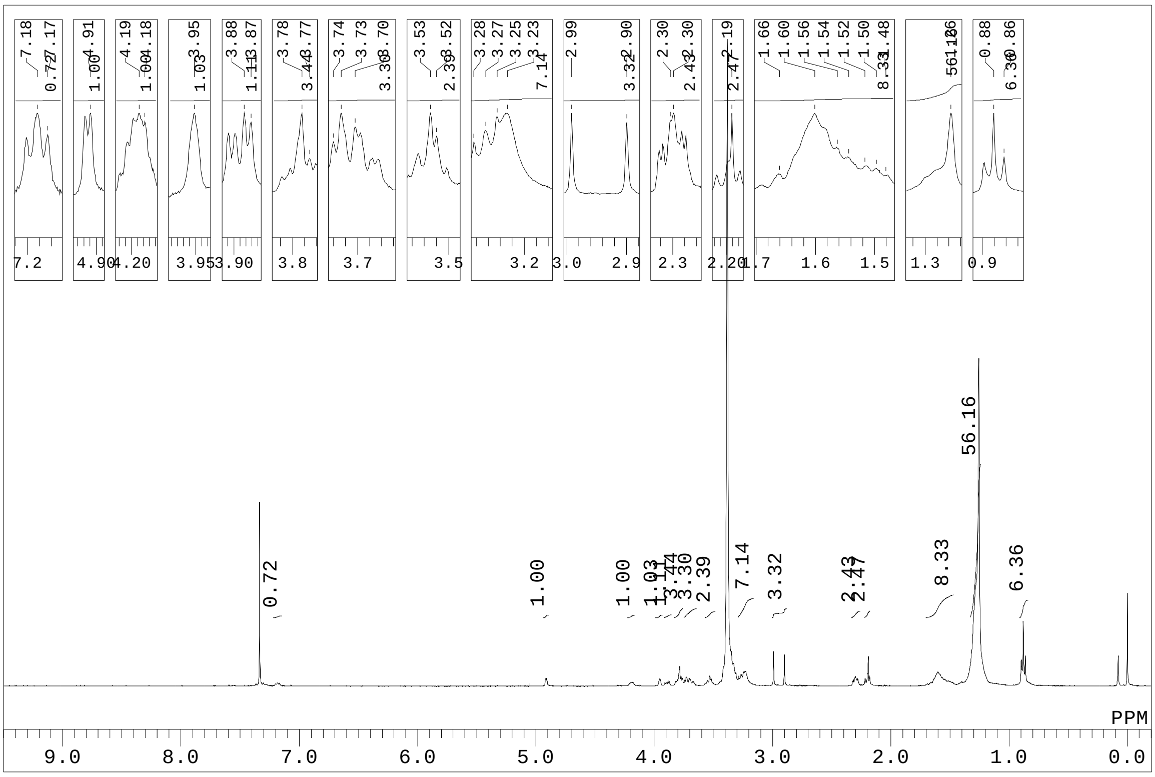

^{13}C NMR spectrum for compound 4a

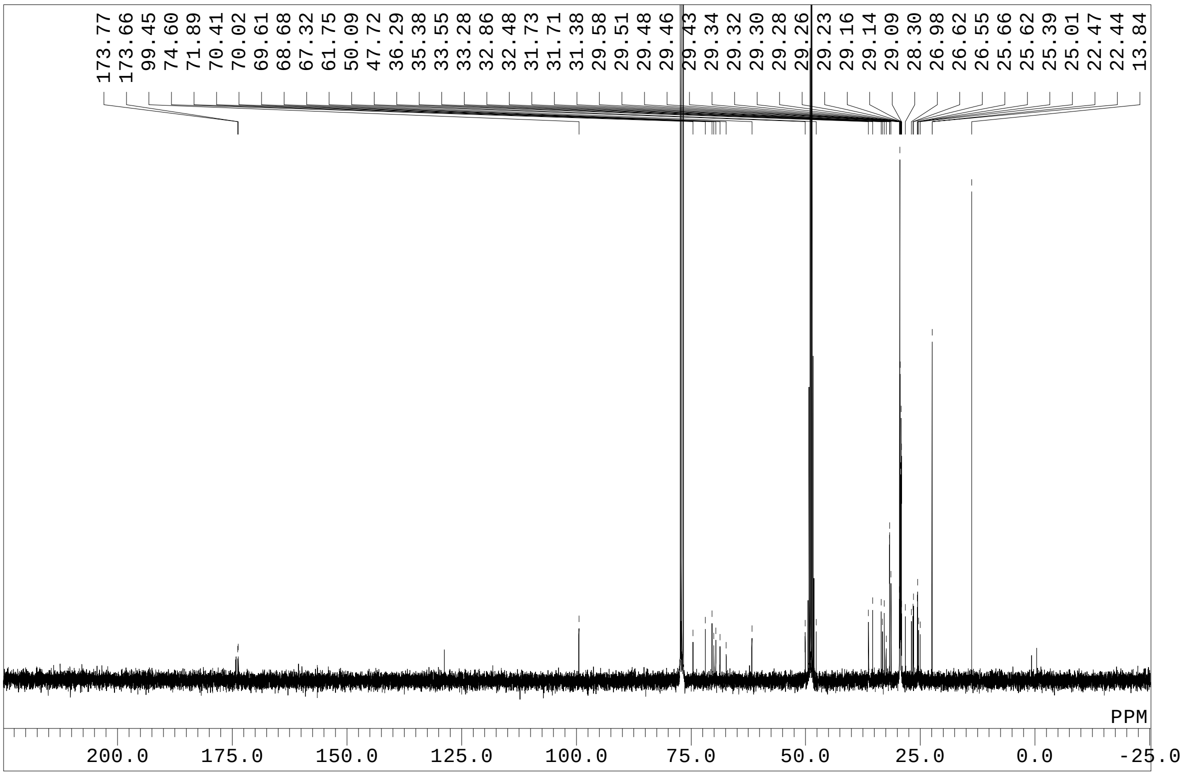

^{1}H NMR spectrum for compound 4b

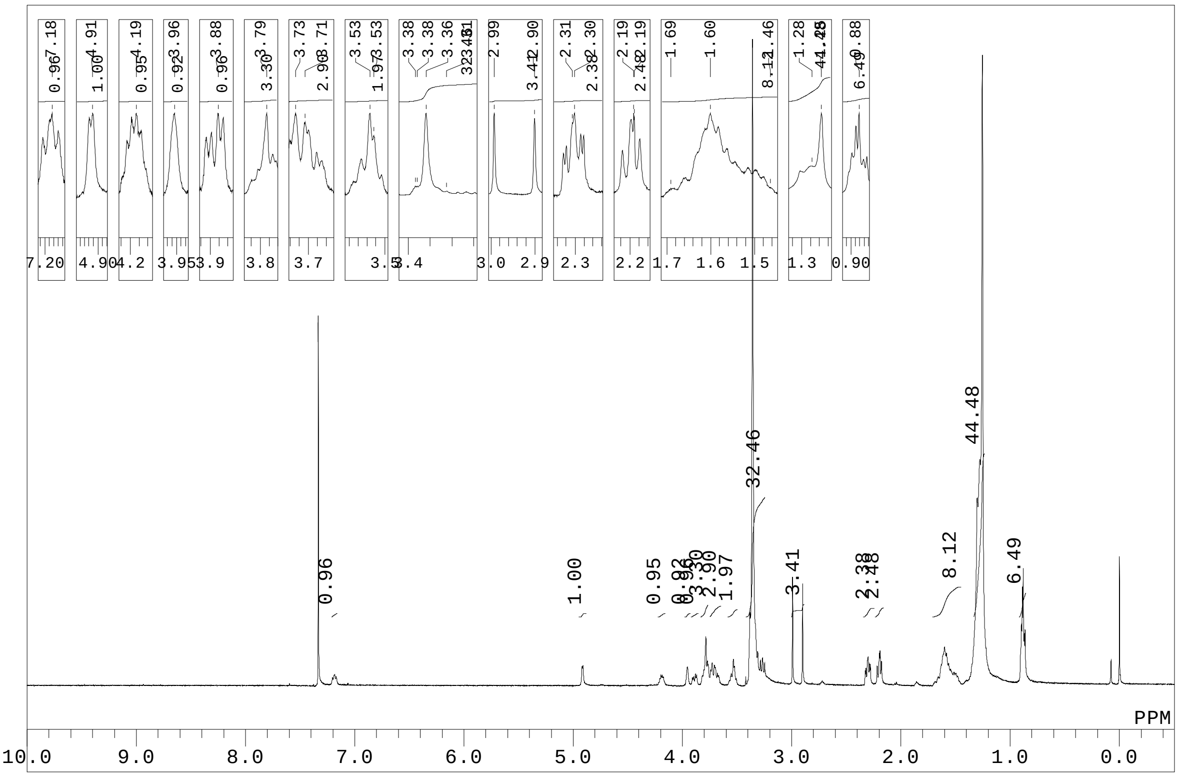

^{13}C NMR spectrum for compound 4b

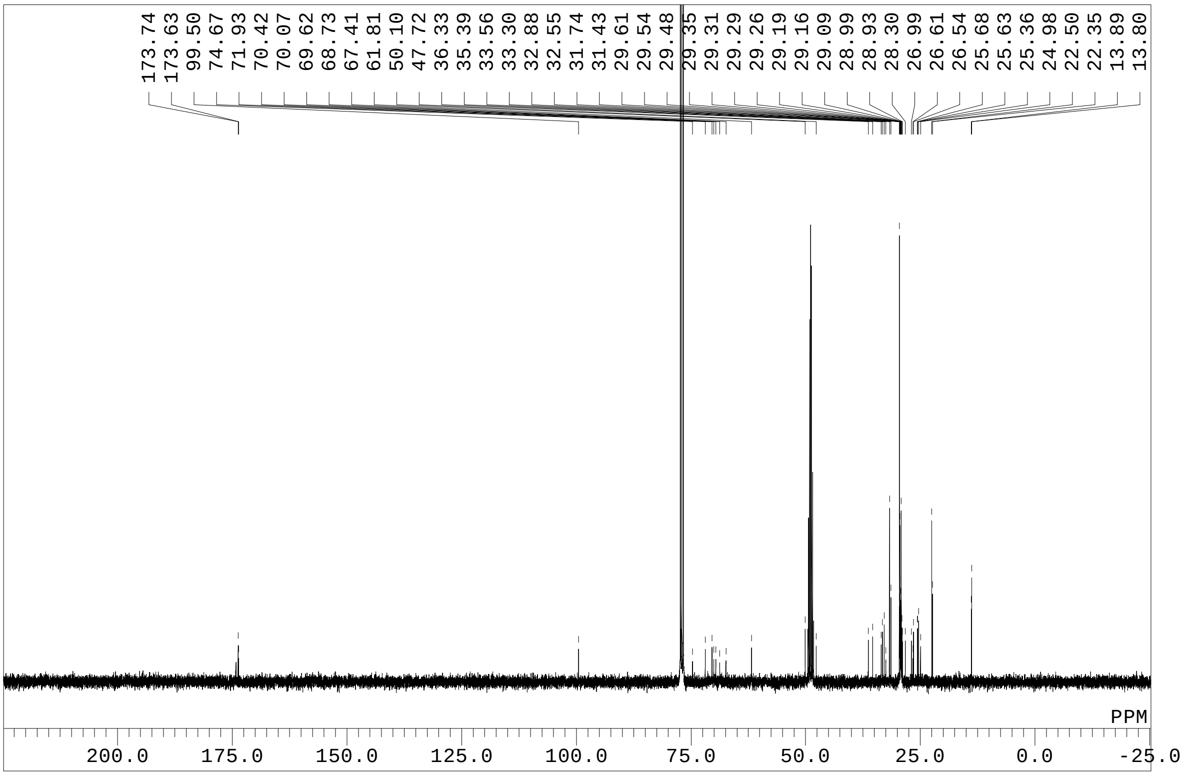

^{1}H NMR spectrum for compound 5a

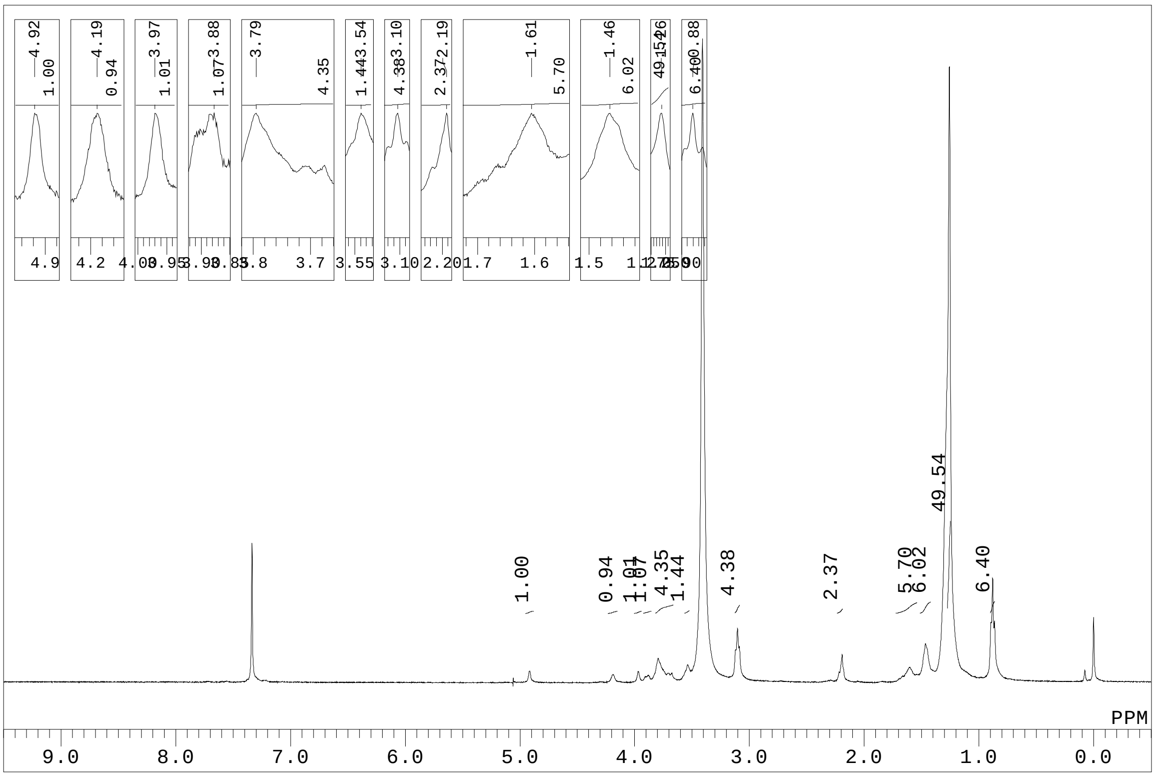

^{13}C NMR spectrum for compound 5a

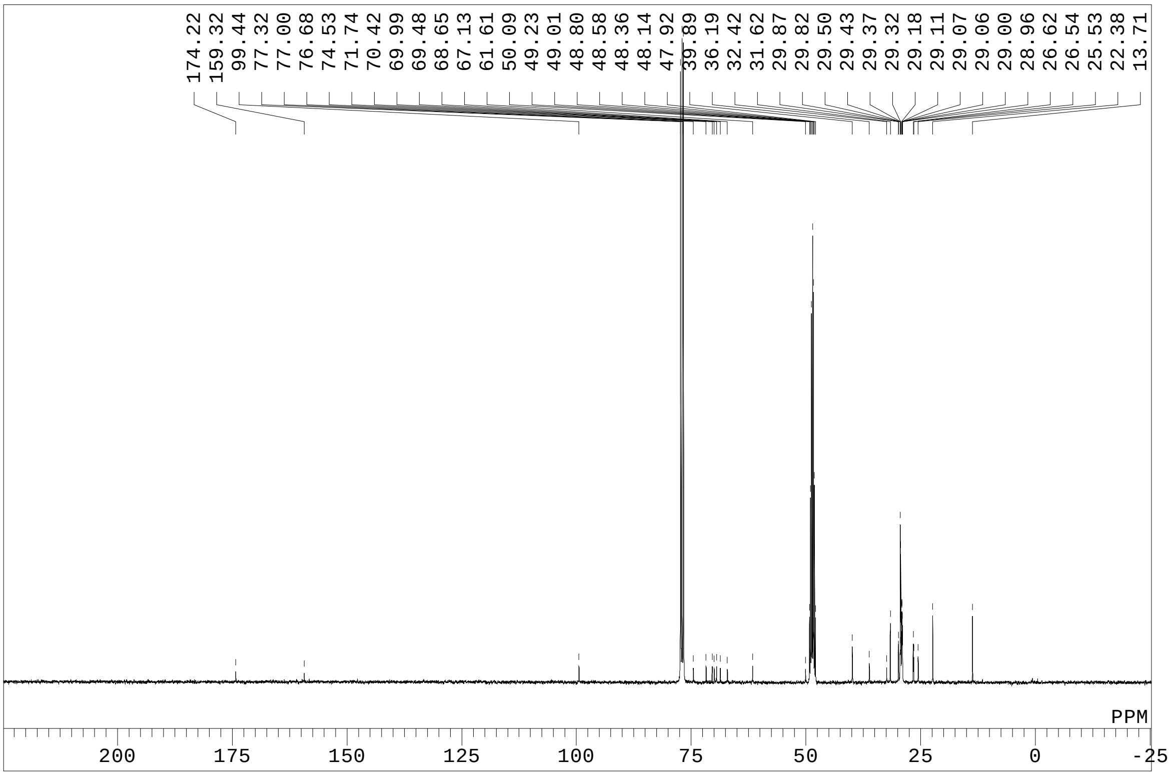

^{1}H NMR spectrum for compound 5b

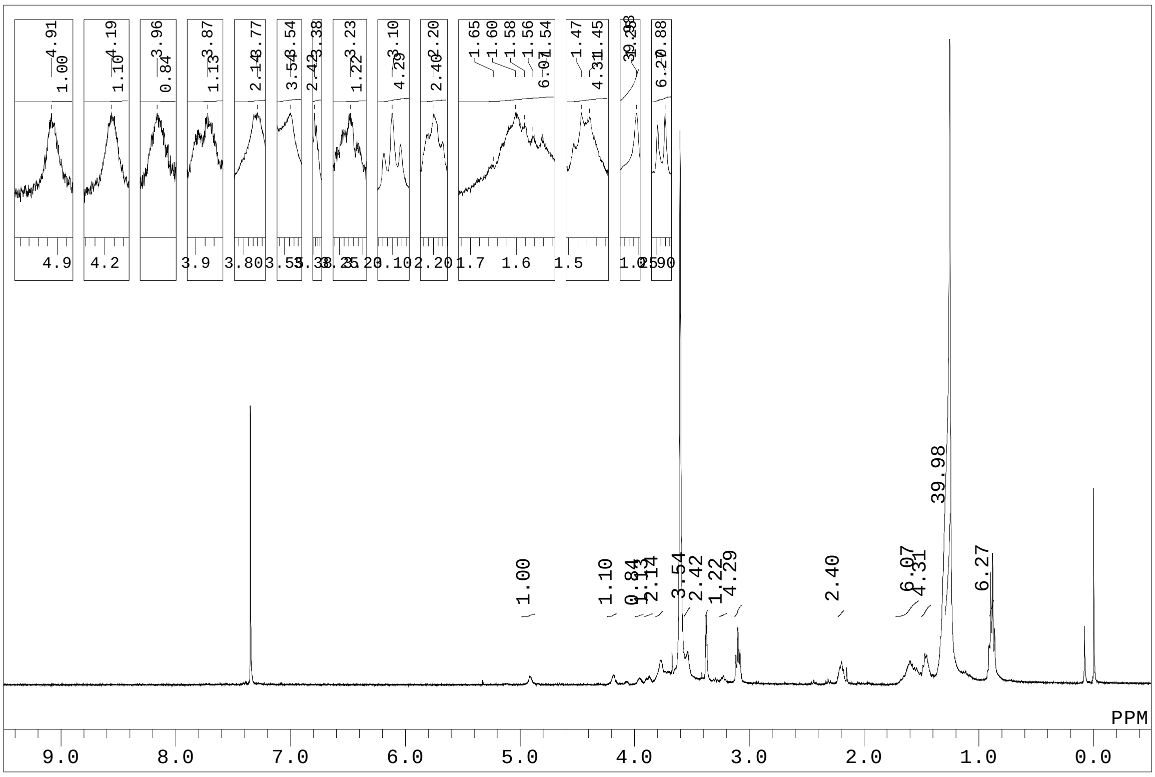

^{13}C NMR spectrum for compound 5b

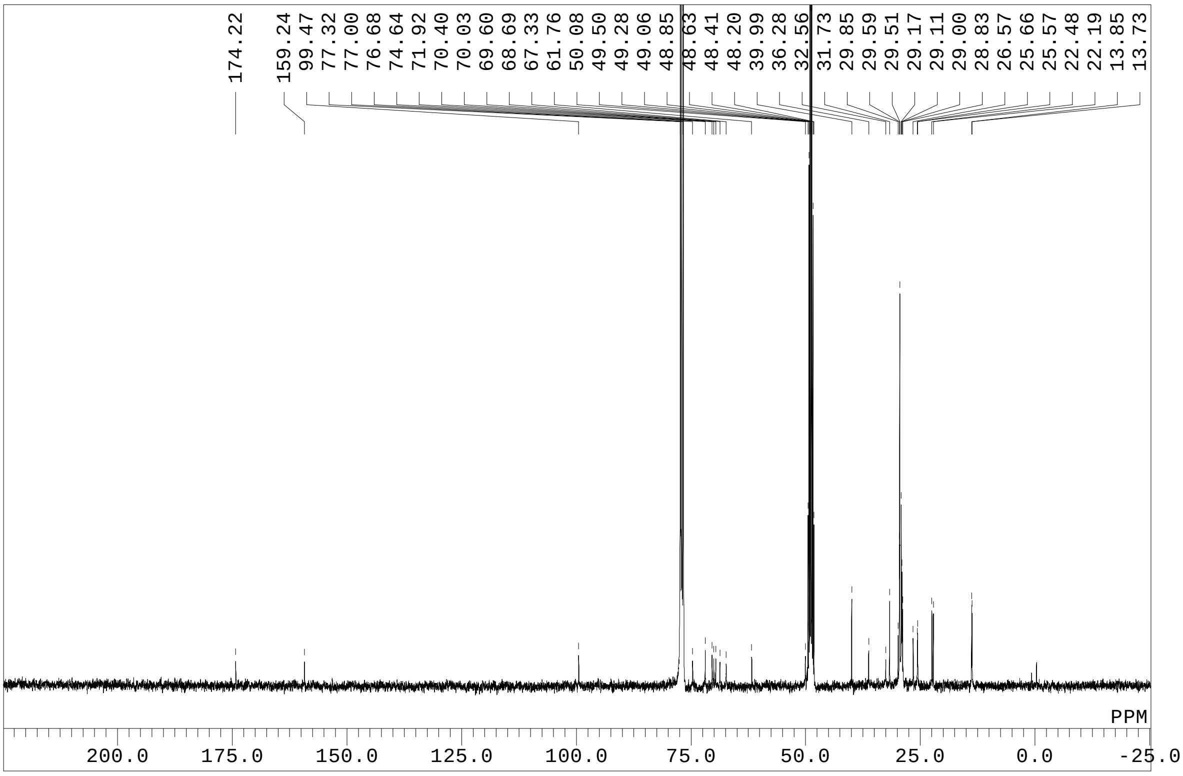

^{1}H NMR spectrum for compound 18a

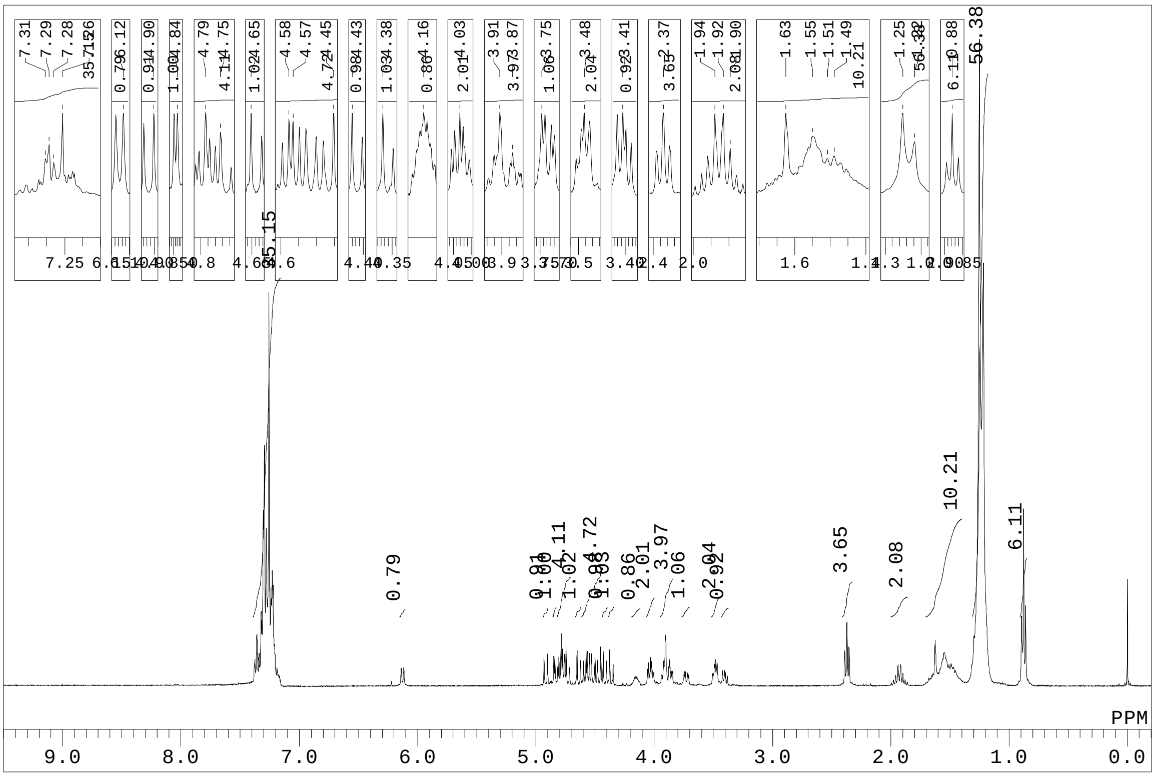

^{13}C NMR spectrum for compound 18a

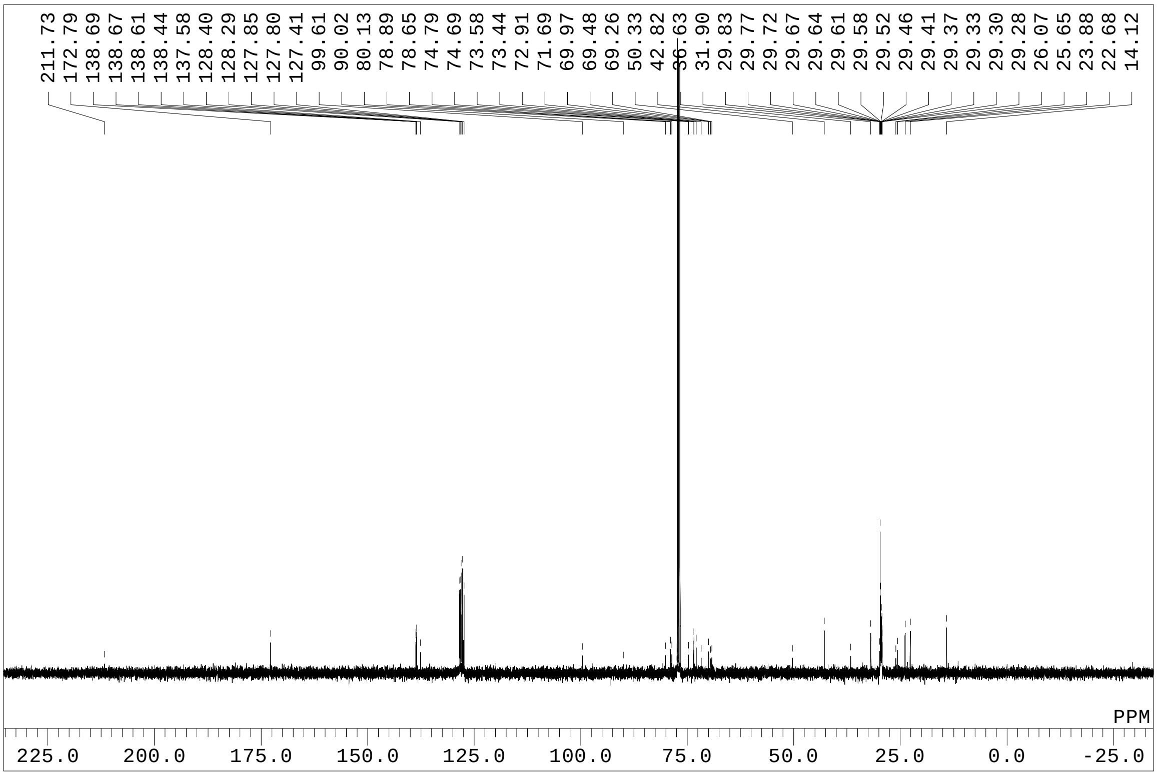

^{1}H NMR spectrum for compound 18b

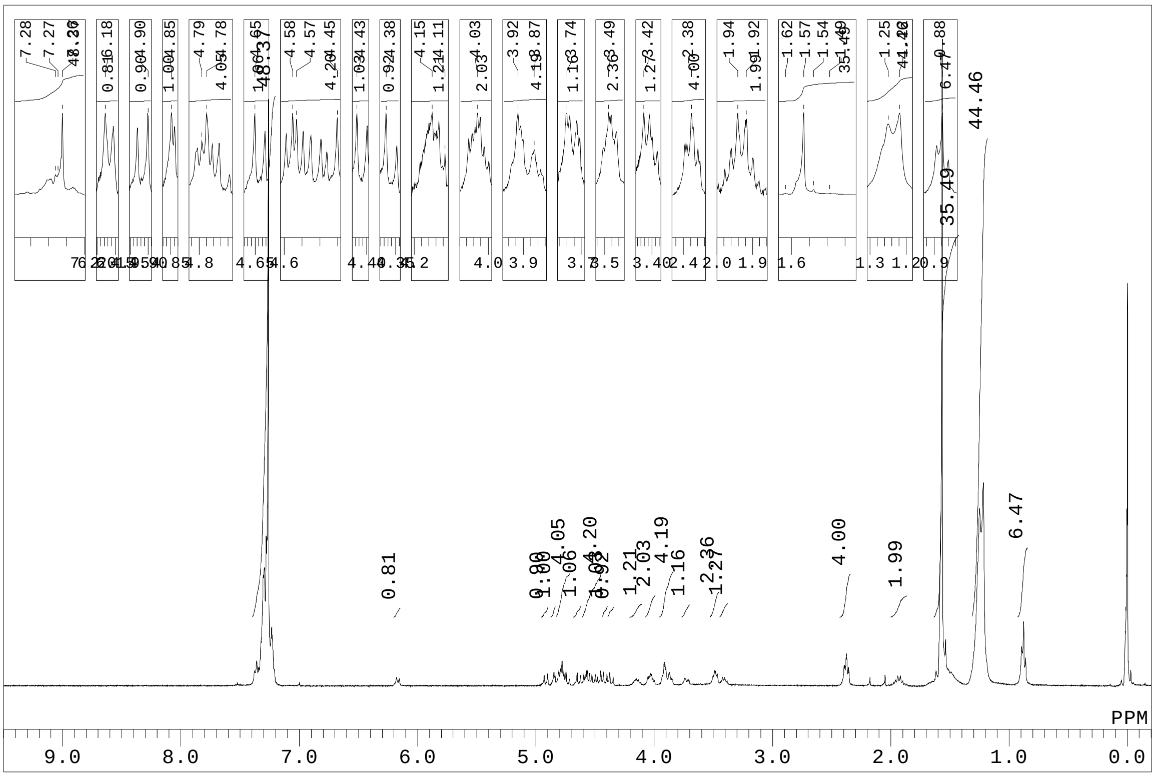

^{13}C NMR spectrum for compound 18b

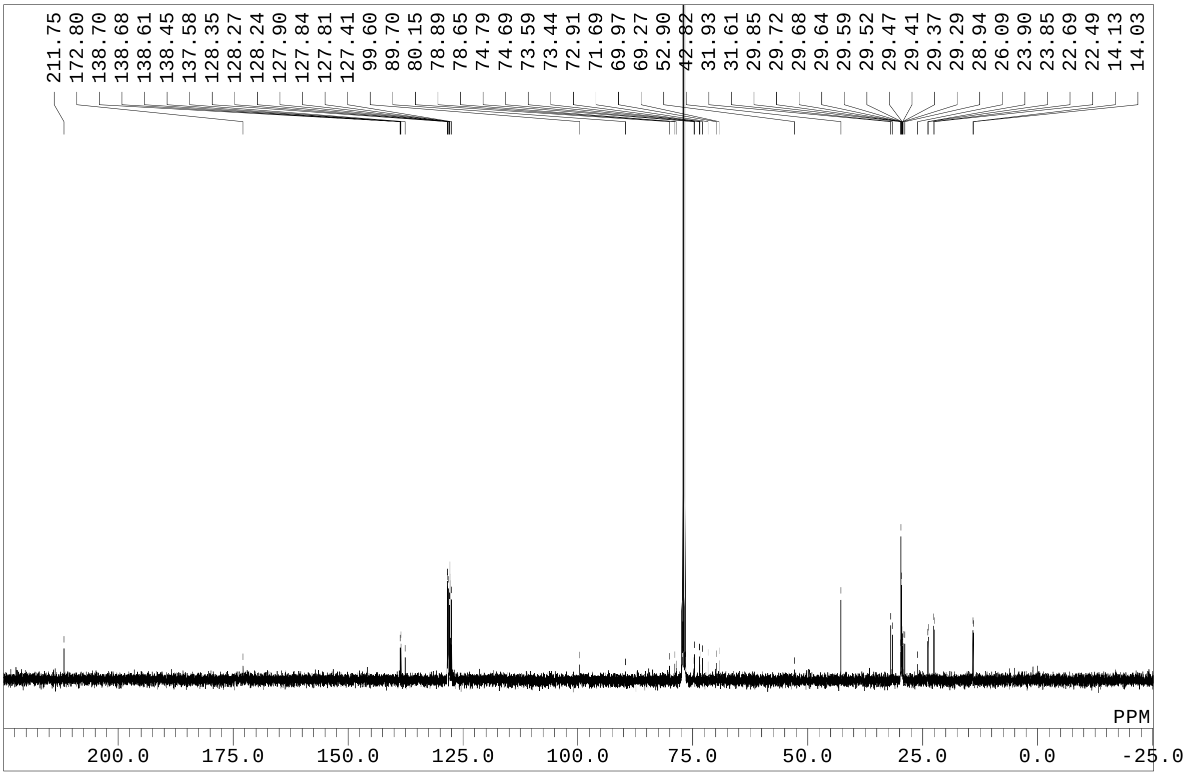

^{1}H NMR spectrum for compound 7a

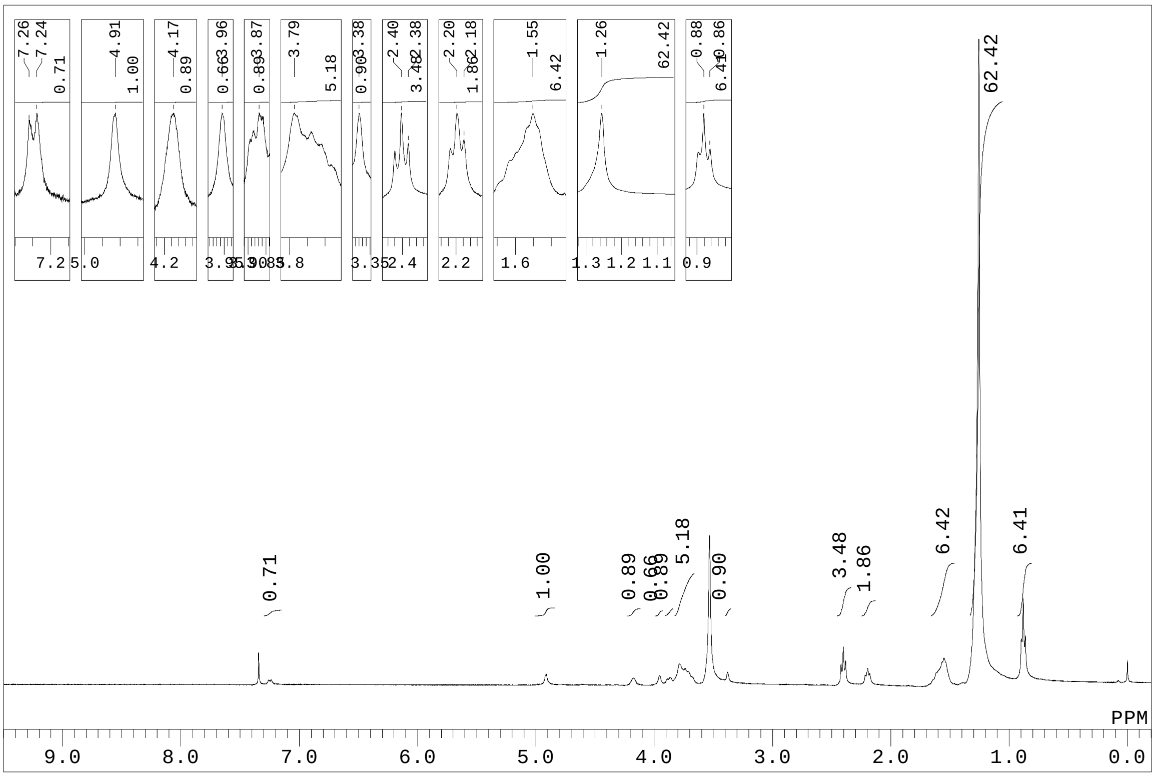

^{13}C NMR spectrum for compound 7a

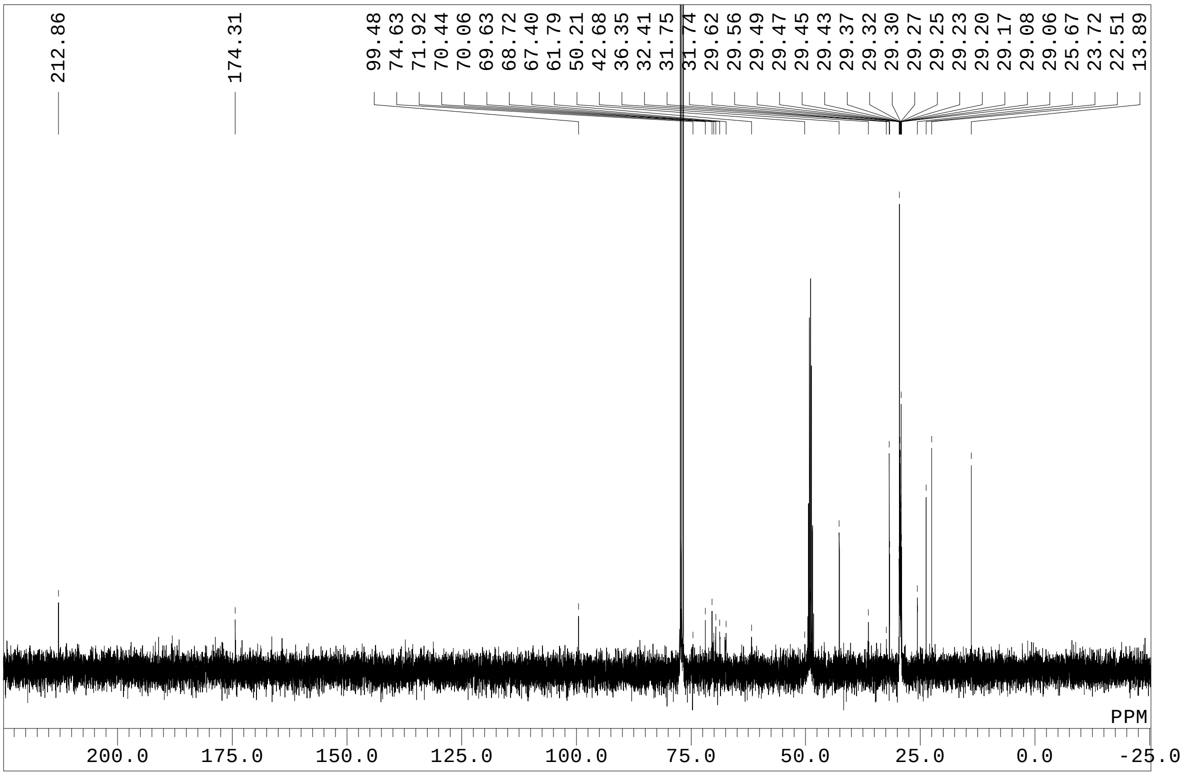

^{1}H NMR spectrum for compound 7b

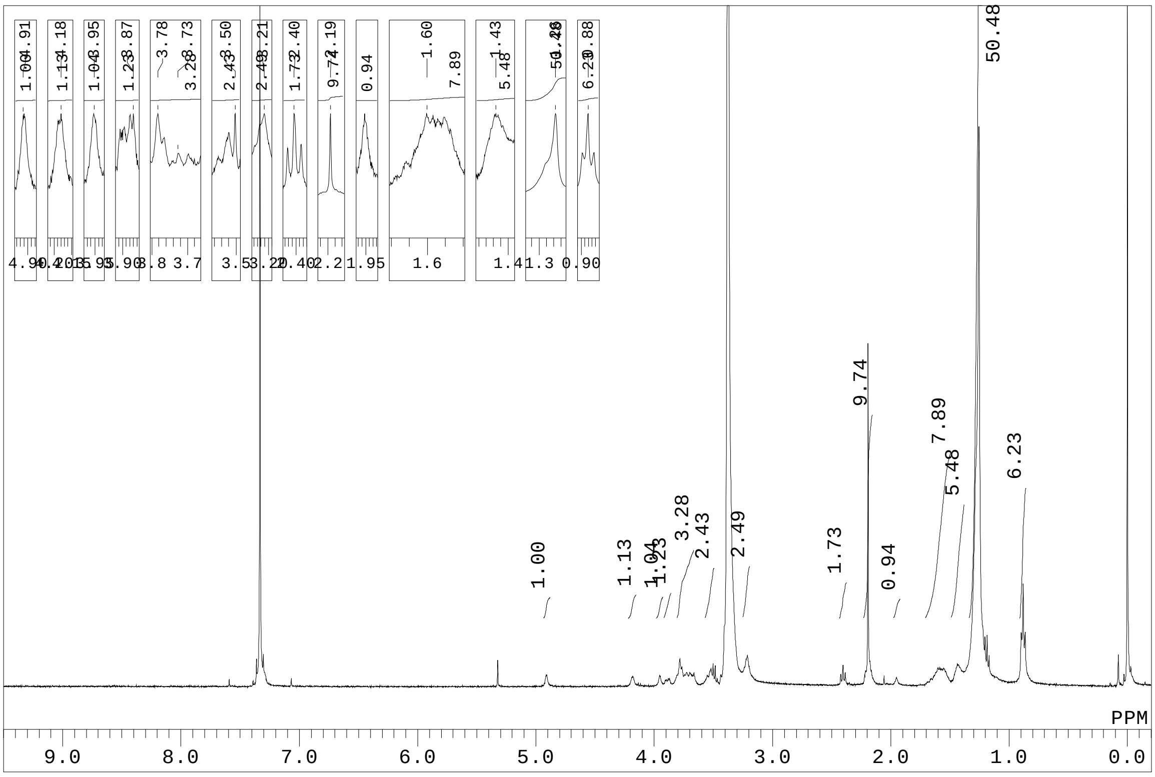

^{13}C NMR spectrum for compound 7b

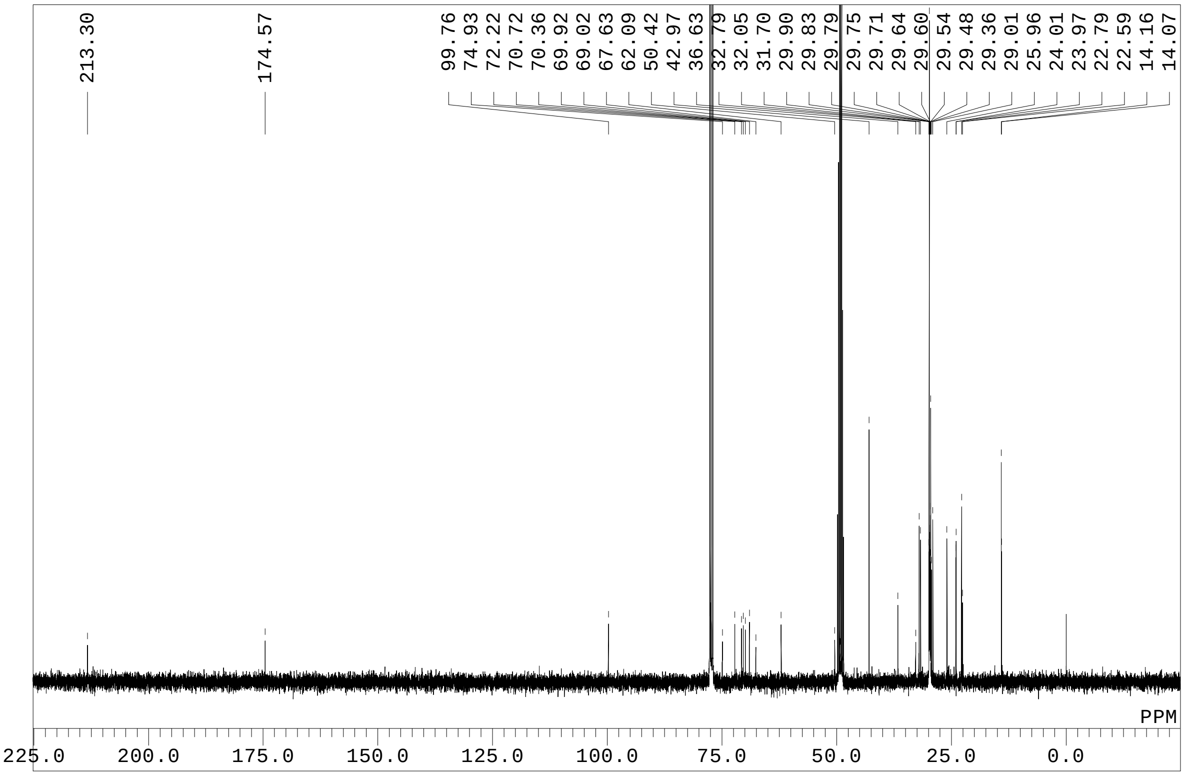

^{1}H NMR spectrum for compound S4

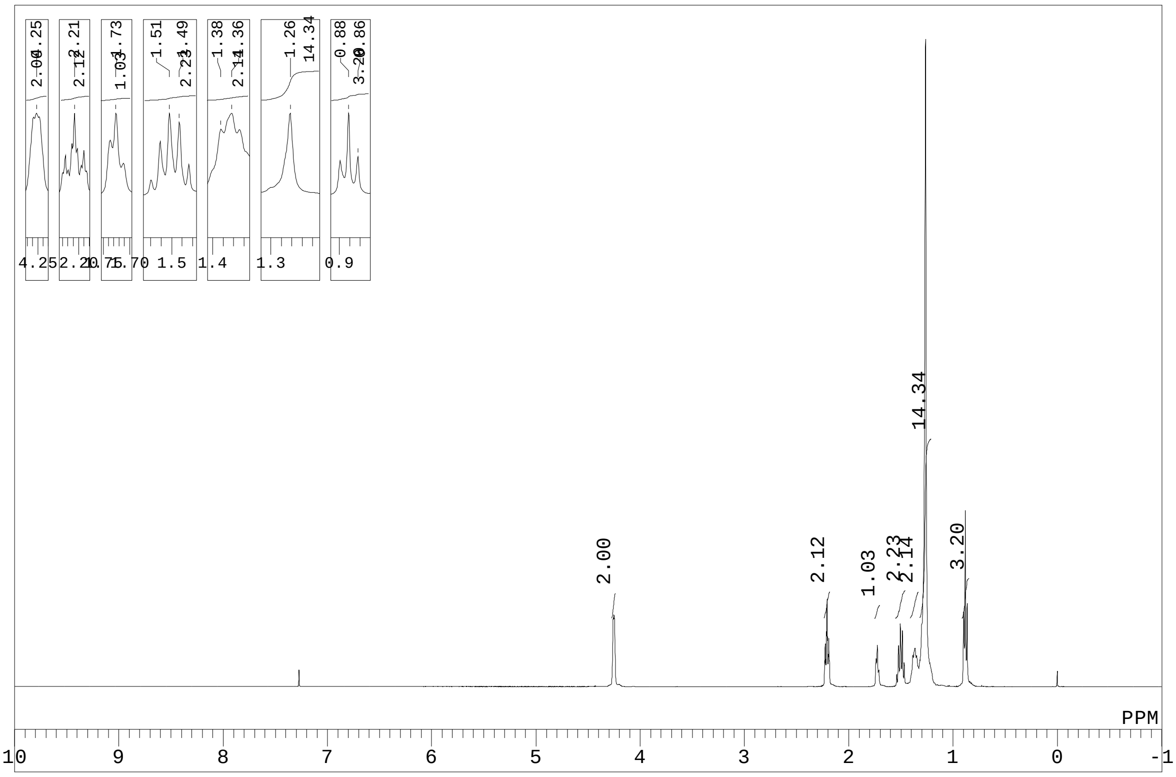

^{13}C NMR spectrum for compound S4

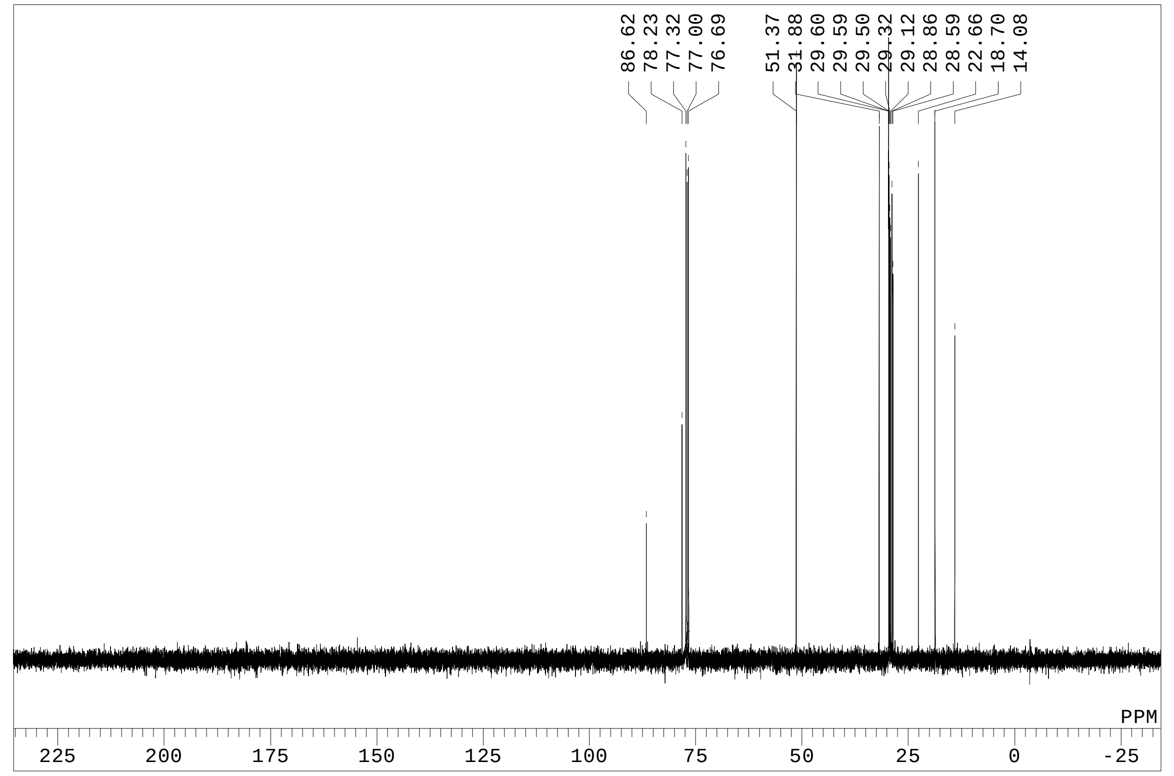

^{1}H NMR spectrum for compound S5

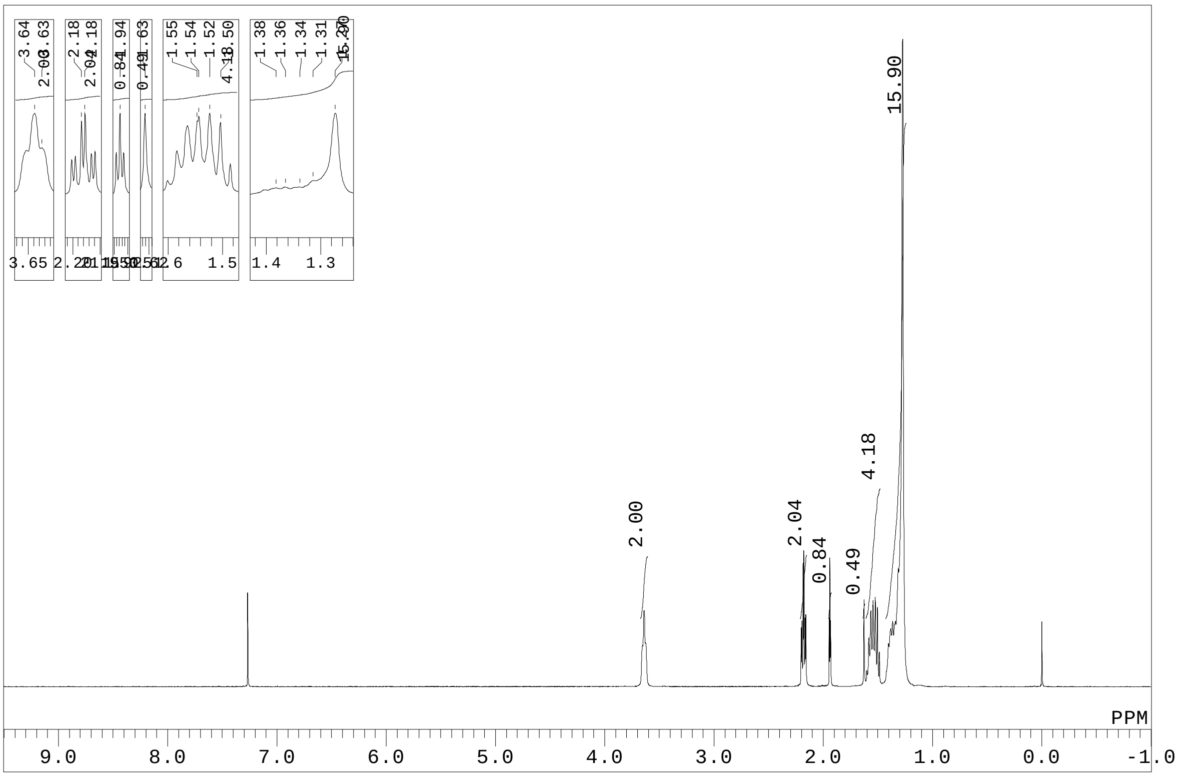

^{13}C NMR spectrum for compound S5

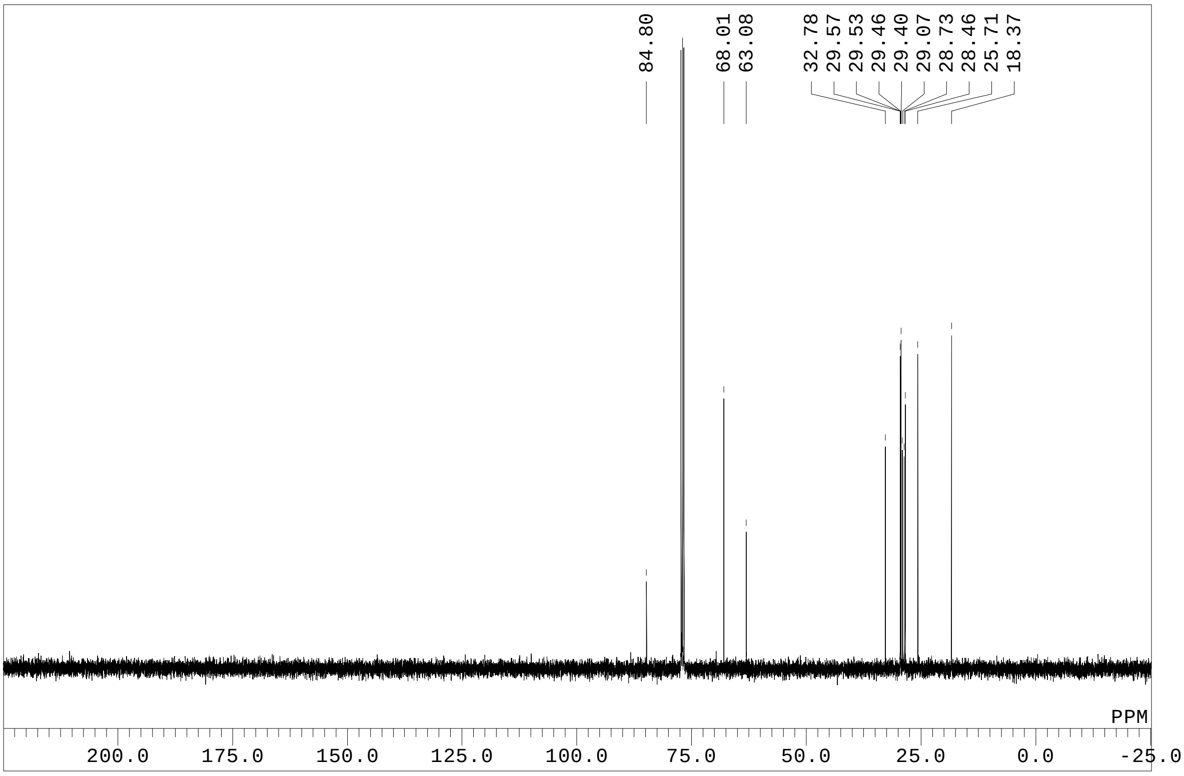

^{1}H NMR spectrum for compound S6

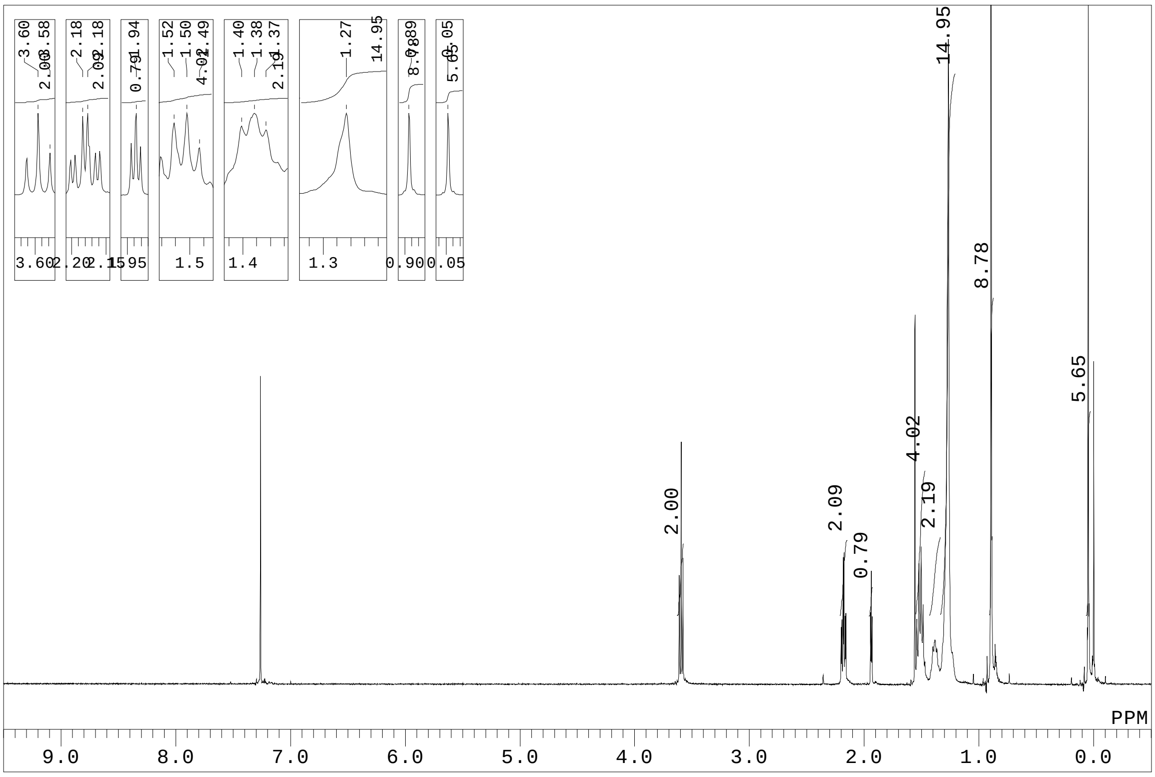

^{13}C NMR spectrum for compound S6

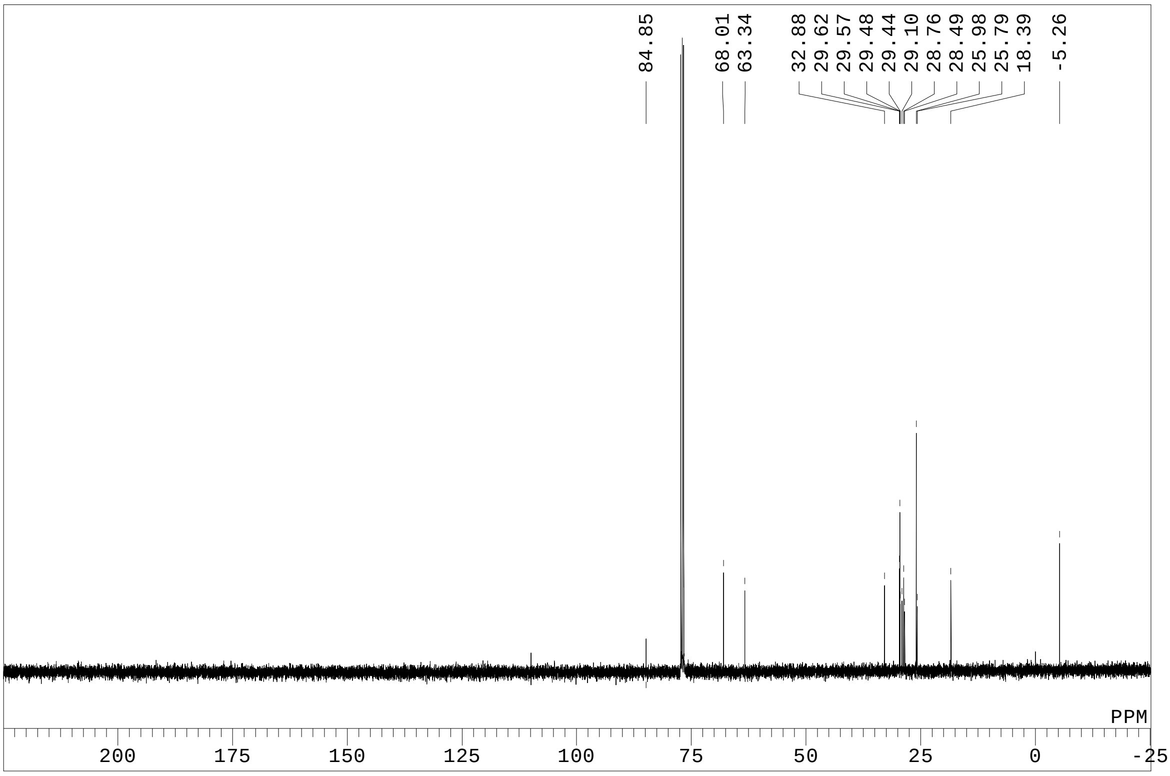

^{1}H NMR spectrum for compound 20a

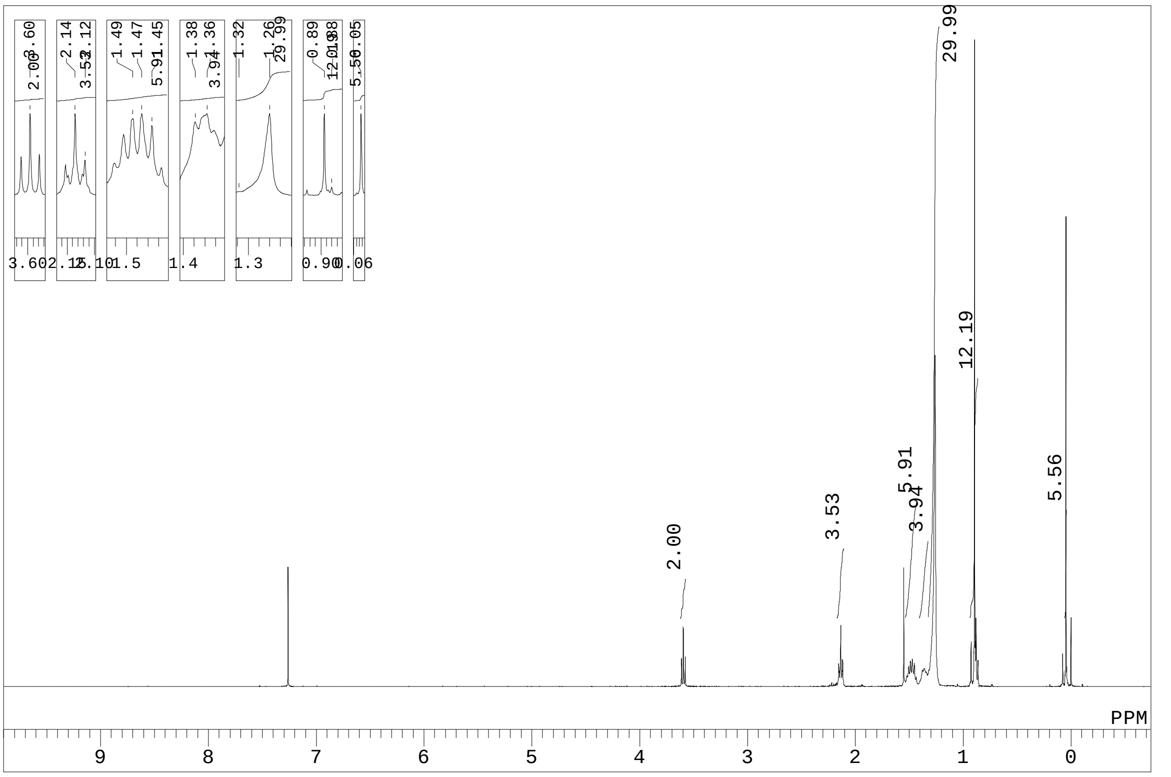

^{13}C NMR spectrum for compound 20a

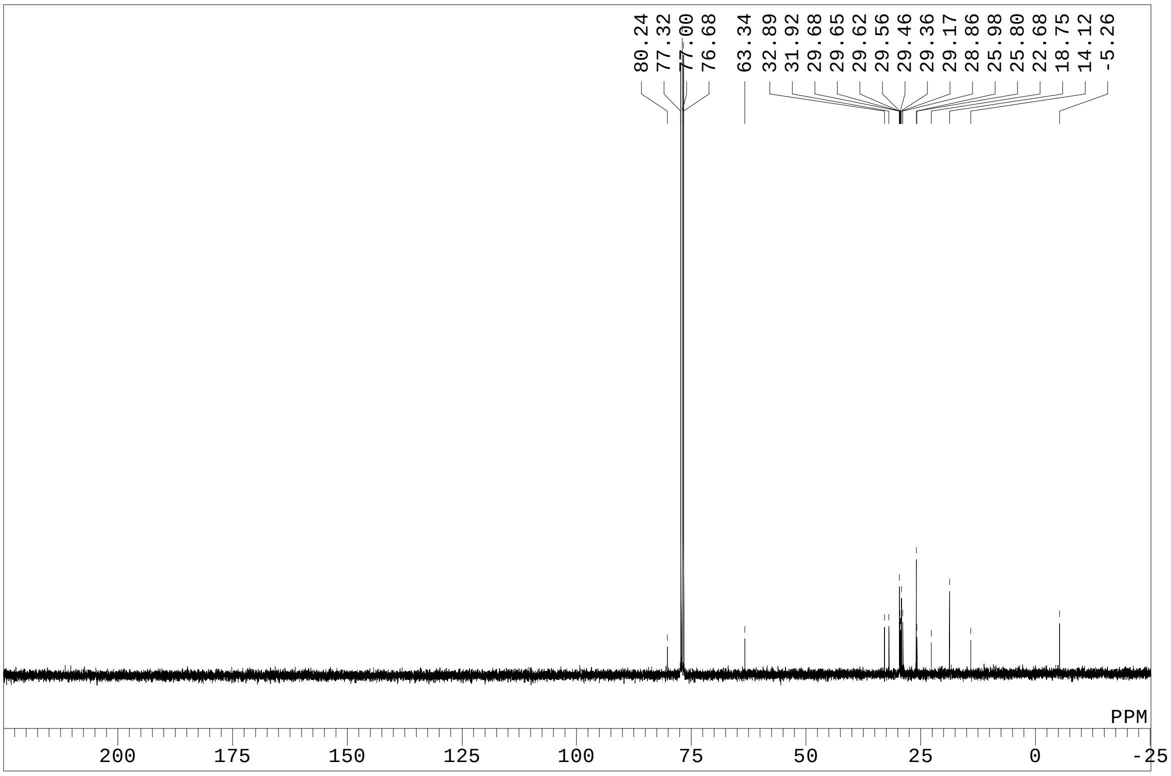

^{1}H NMR spectrum for compound 20b

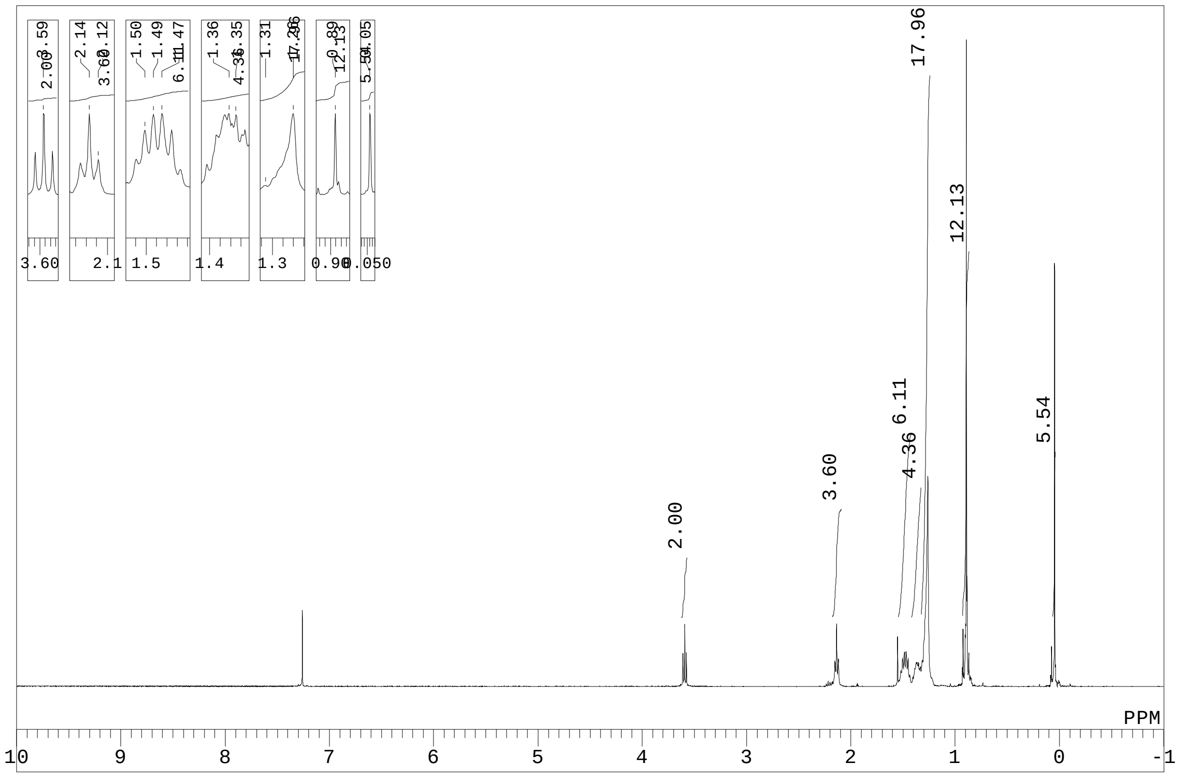

^{13}C NMR spectrum for compound 20b

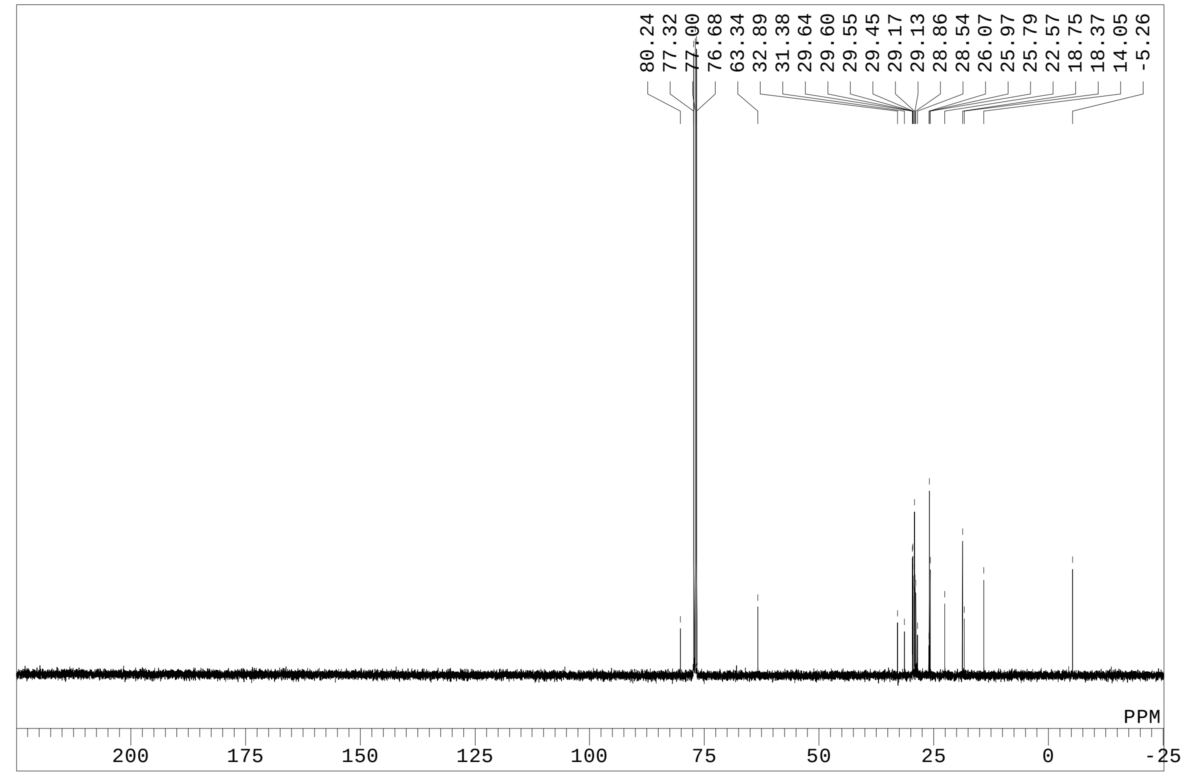

^{1}H NMR spectrum for compound S7

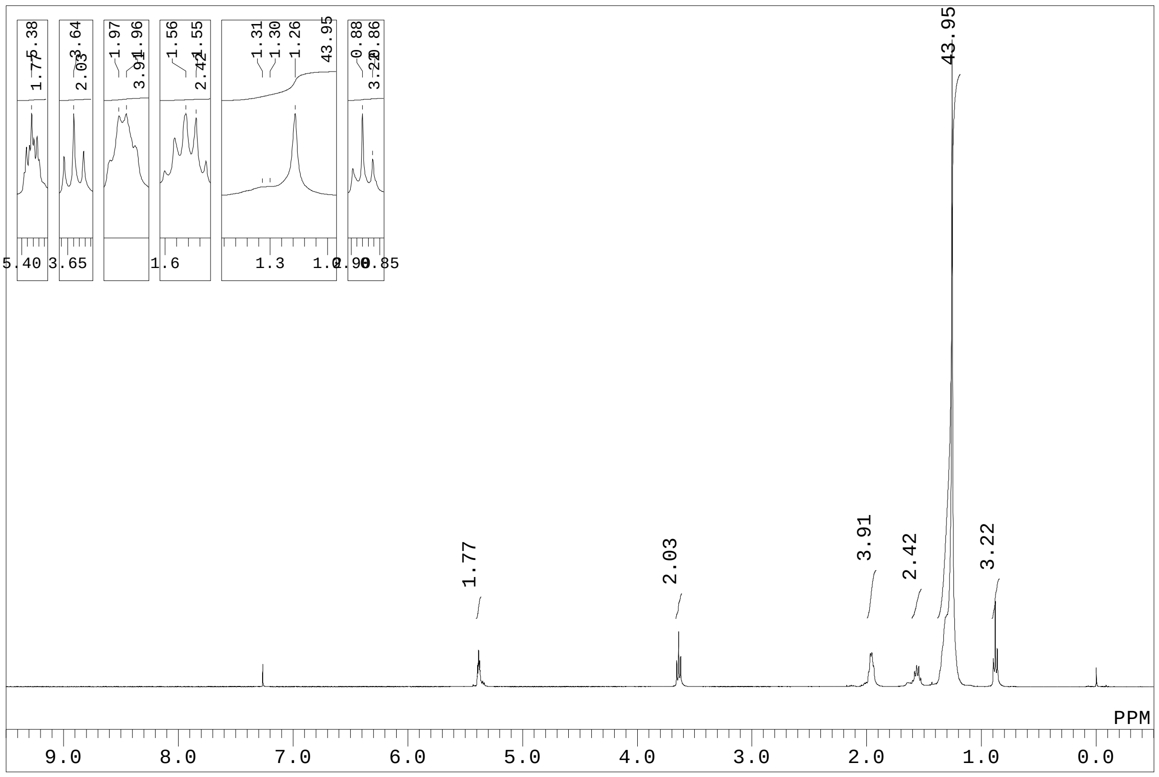

^{13}C NMR spectrum for compound S7

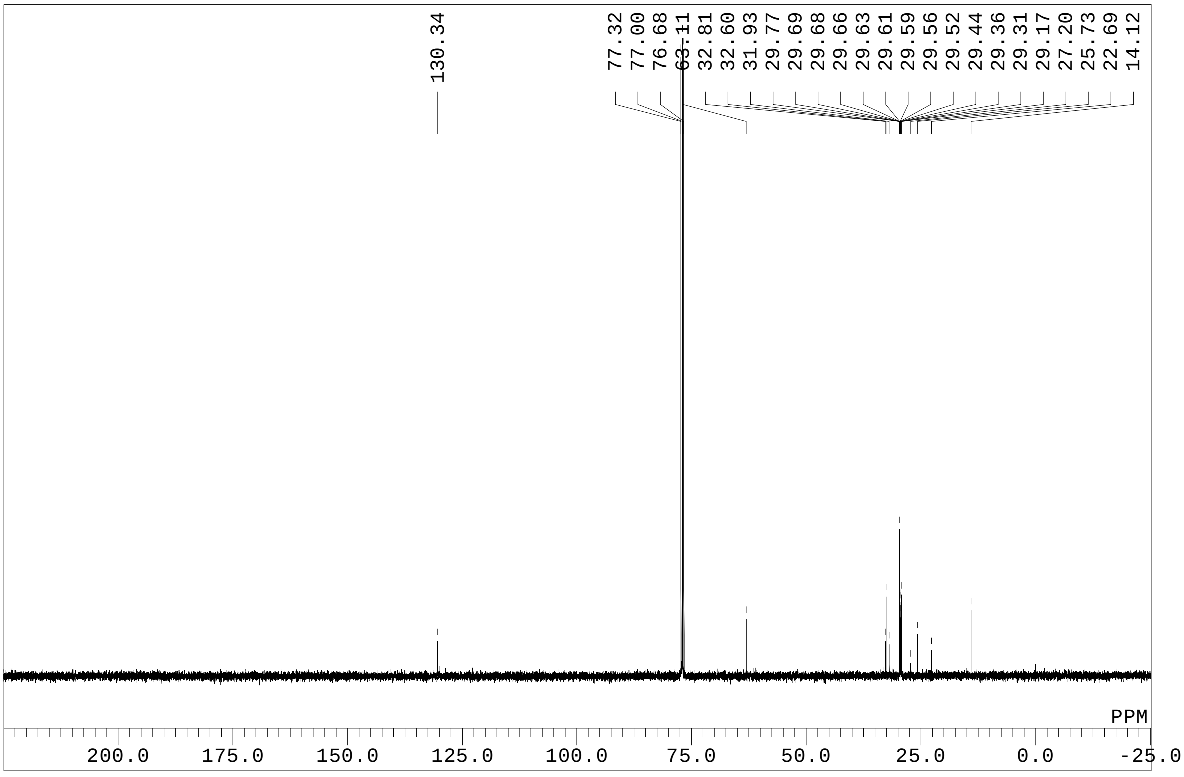

^{1}H NMR spectrum for compound 21a

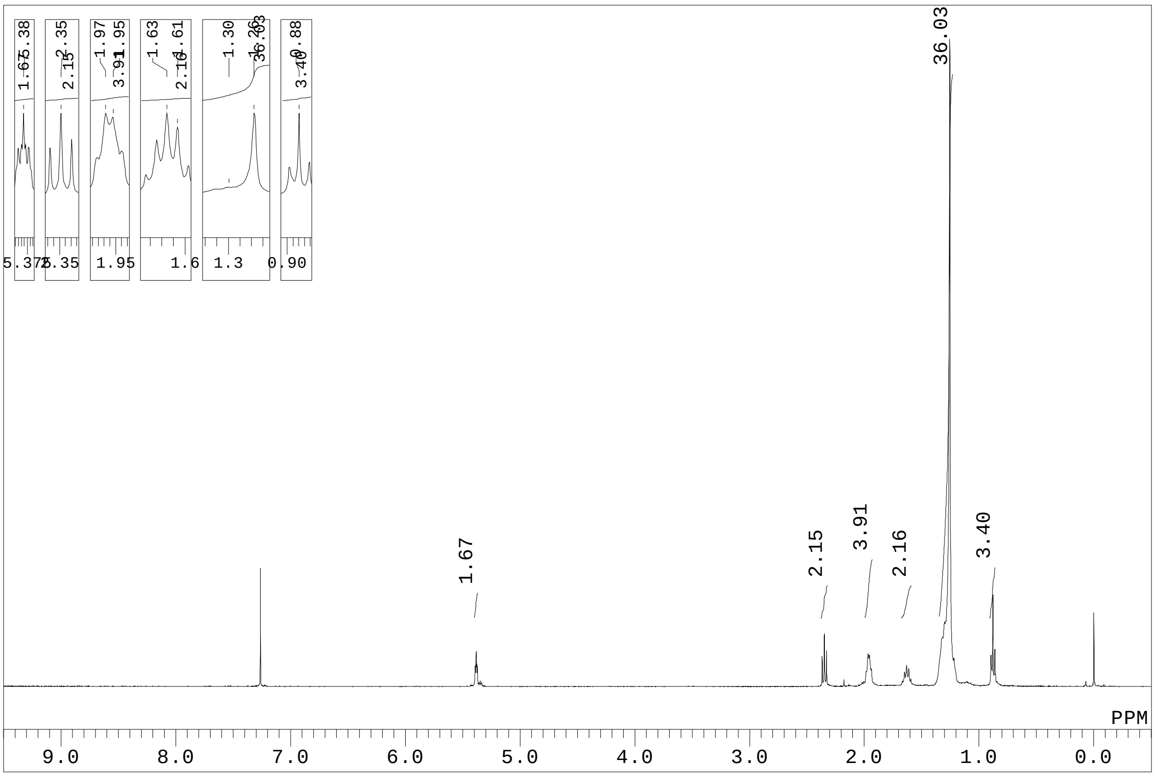

^{13}C NMR spectrum for compound 21a

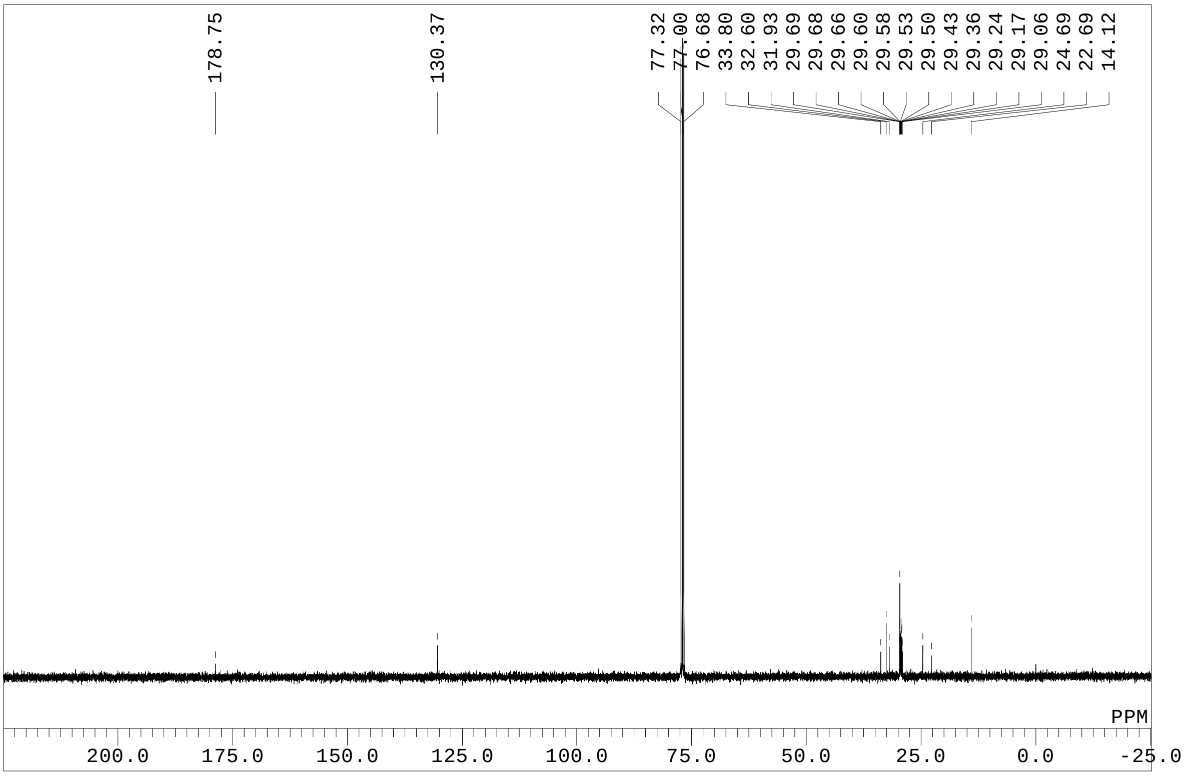

^{1}H NMR spectrum for compound S8

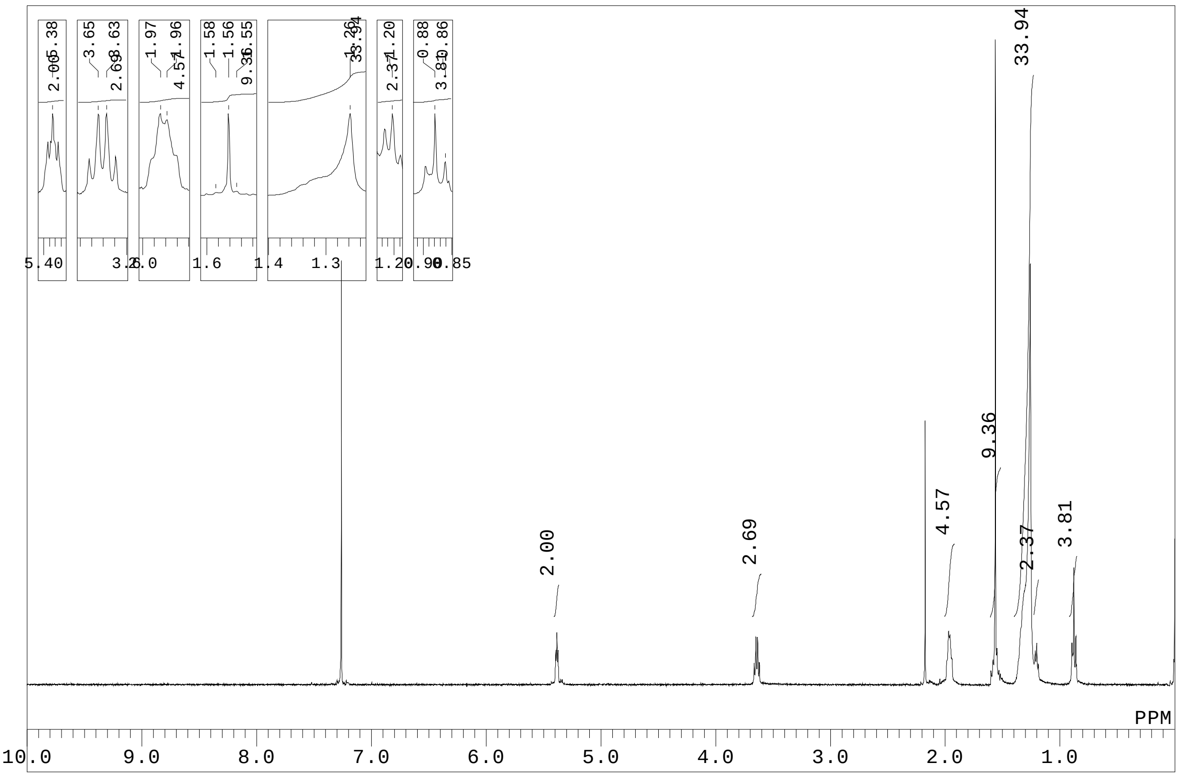

^{13}C NMR spectrum for compound S8

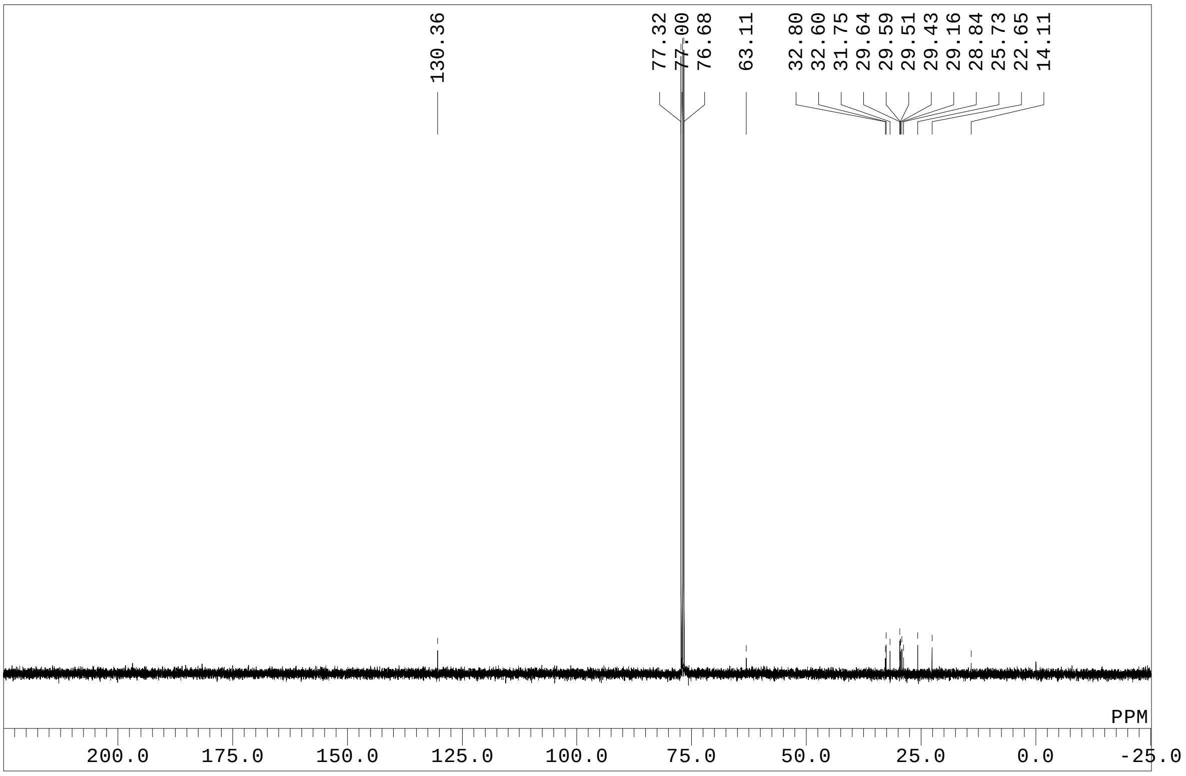

^{1}H NMR spectrum for compound 21b

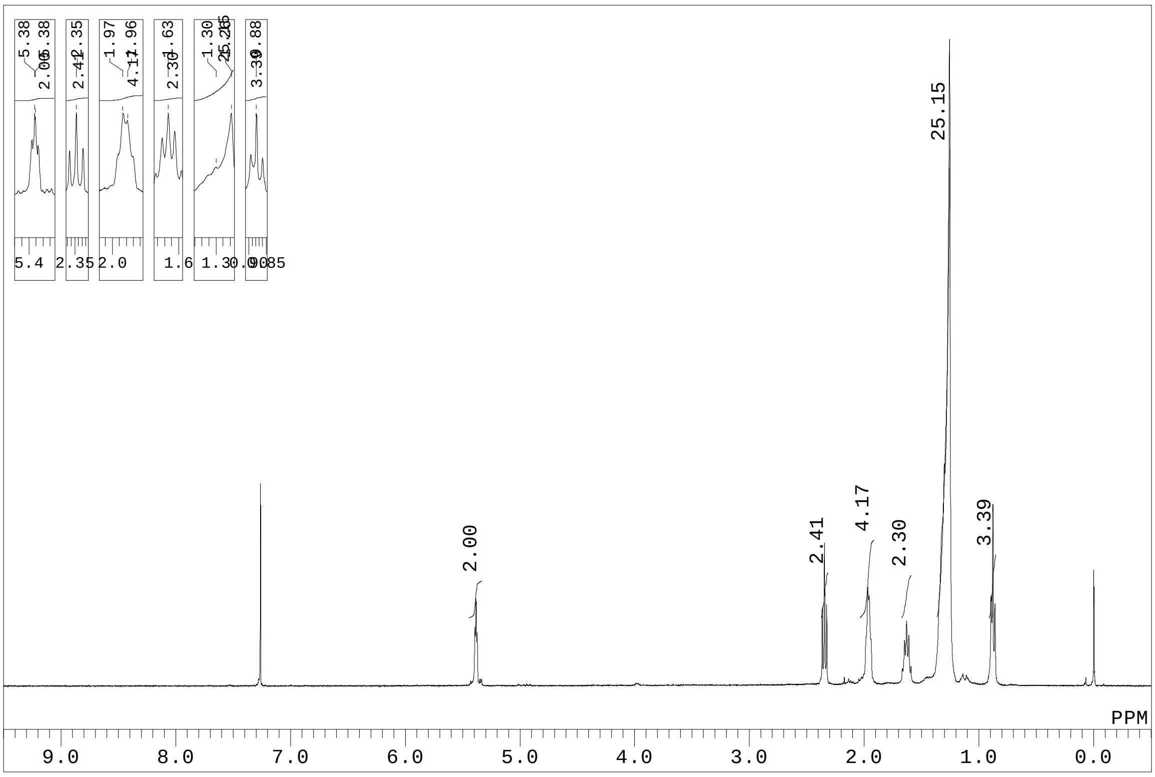

^{13}C NMR spectrum for compound 21b

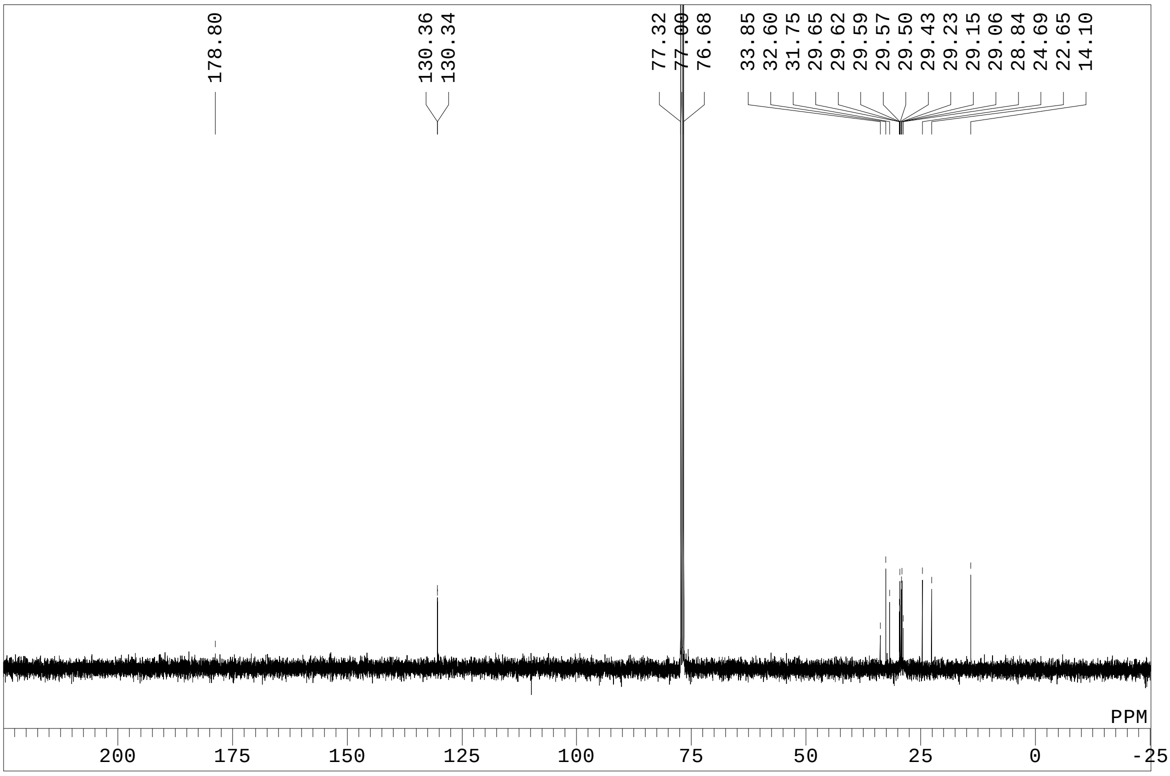

^{1}H NMR spectrum for compound S9

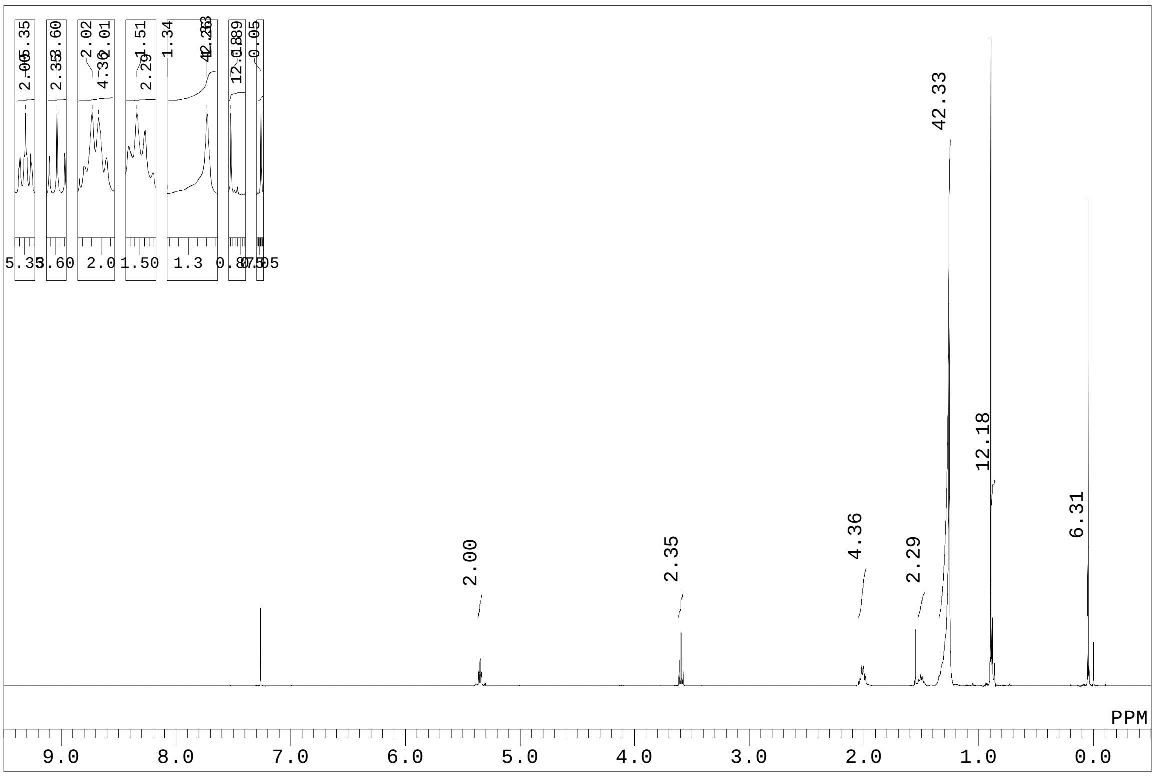

^{13}C NMR spectrum for compound S9

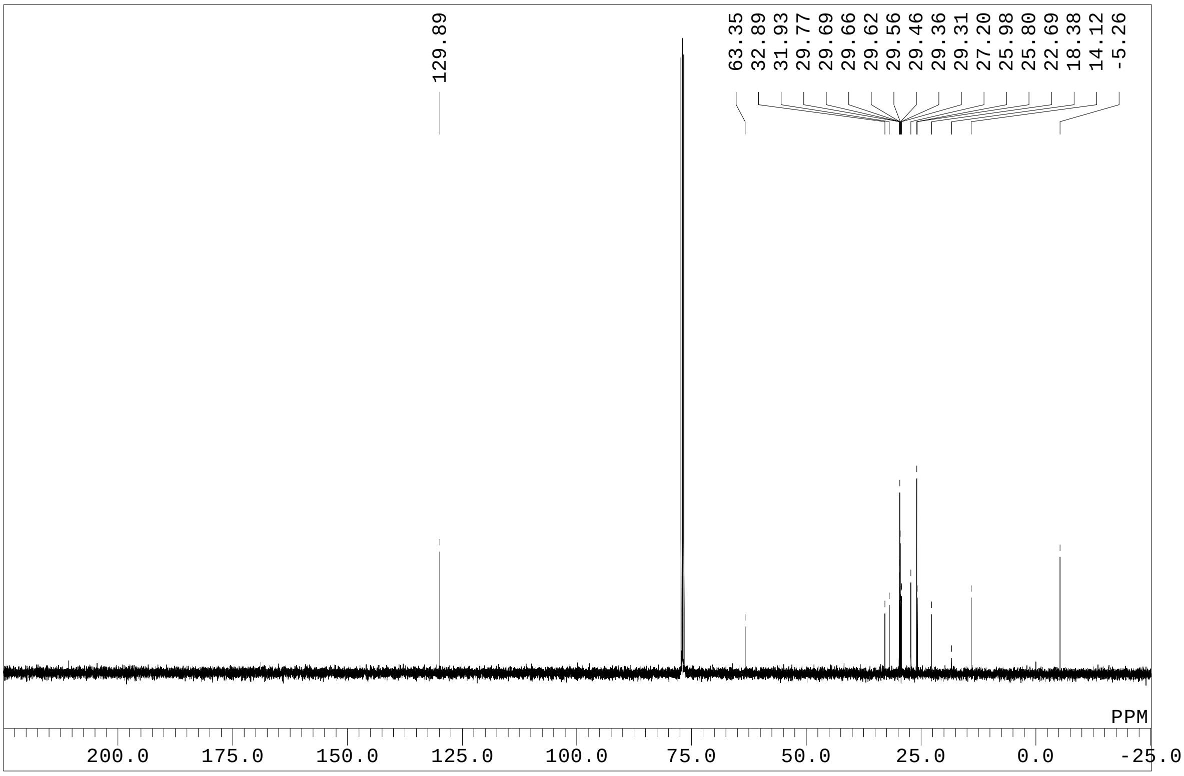

^{1}H NMR spectrum for compound 22a

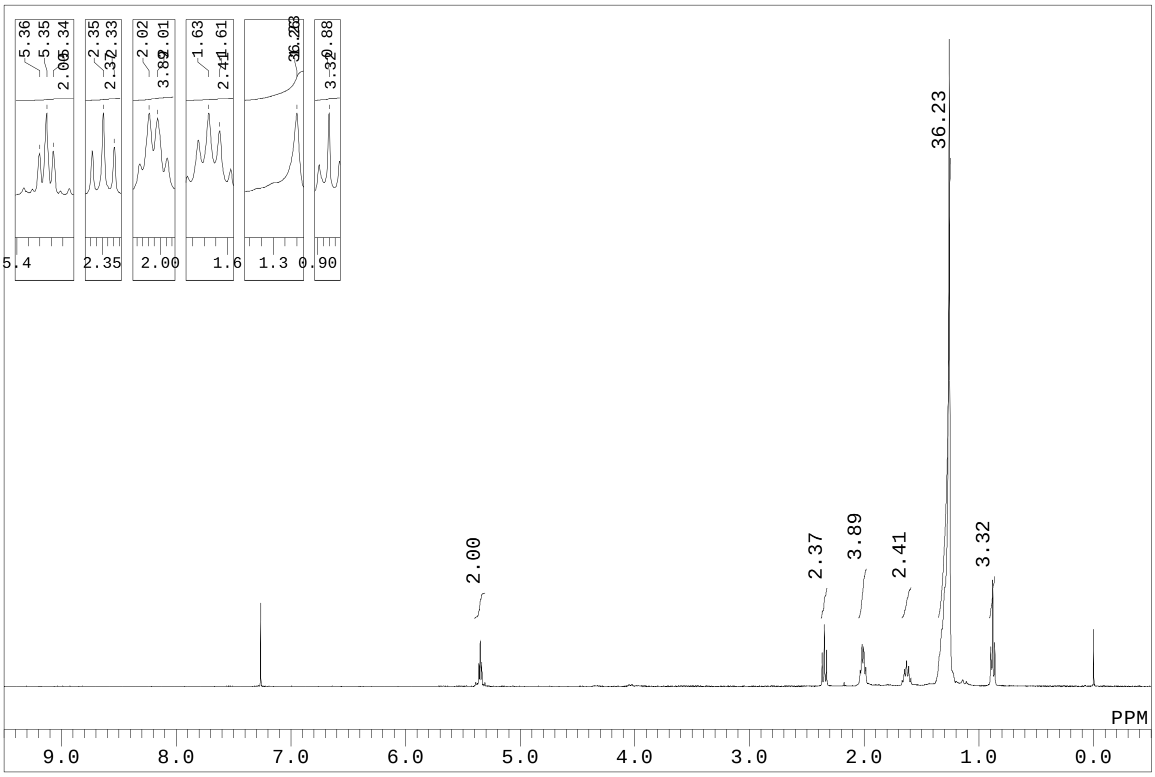

^{13}C NMR spectrum for compound 22a

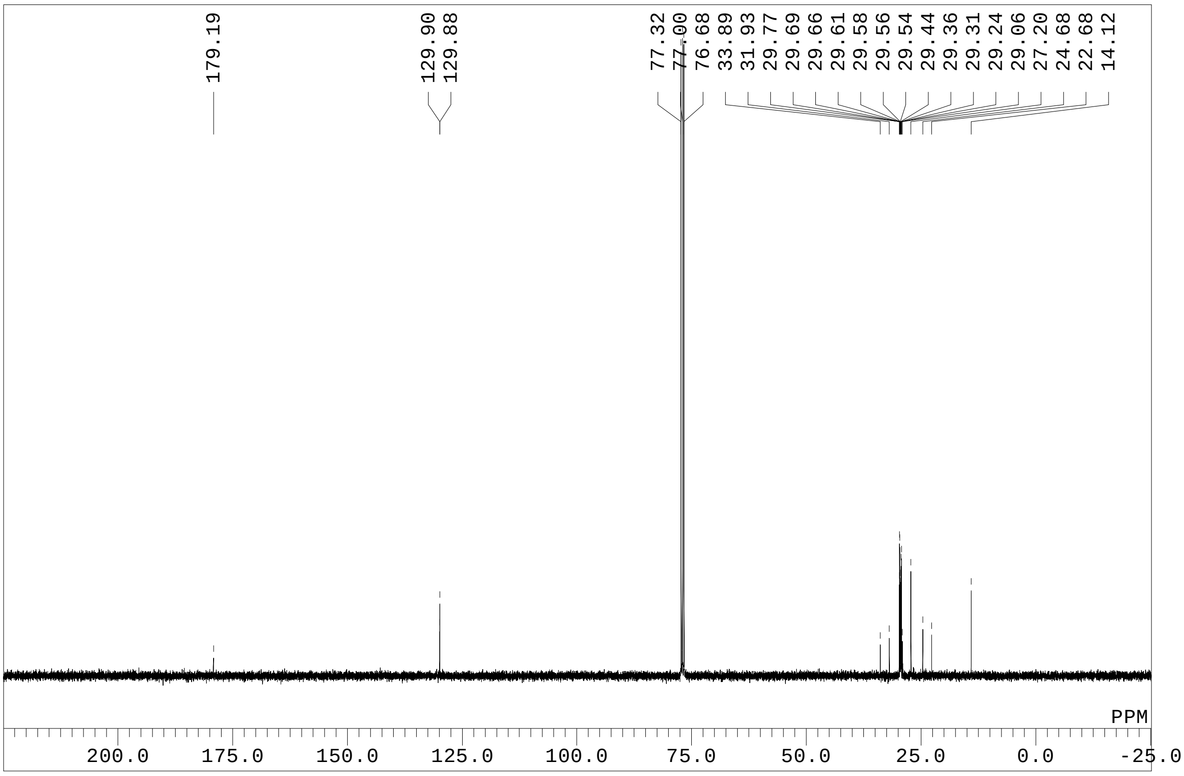

^{1}H NMR spectrum for compound S11

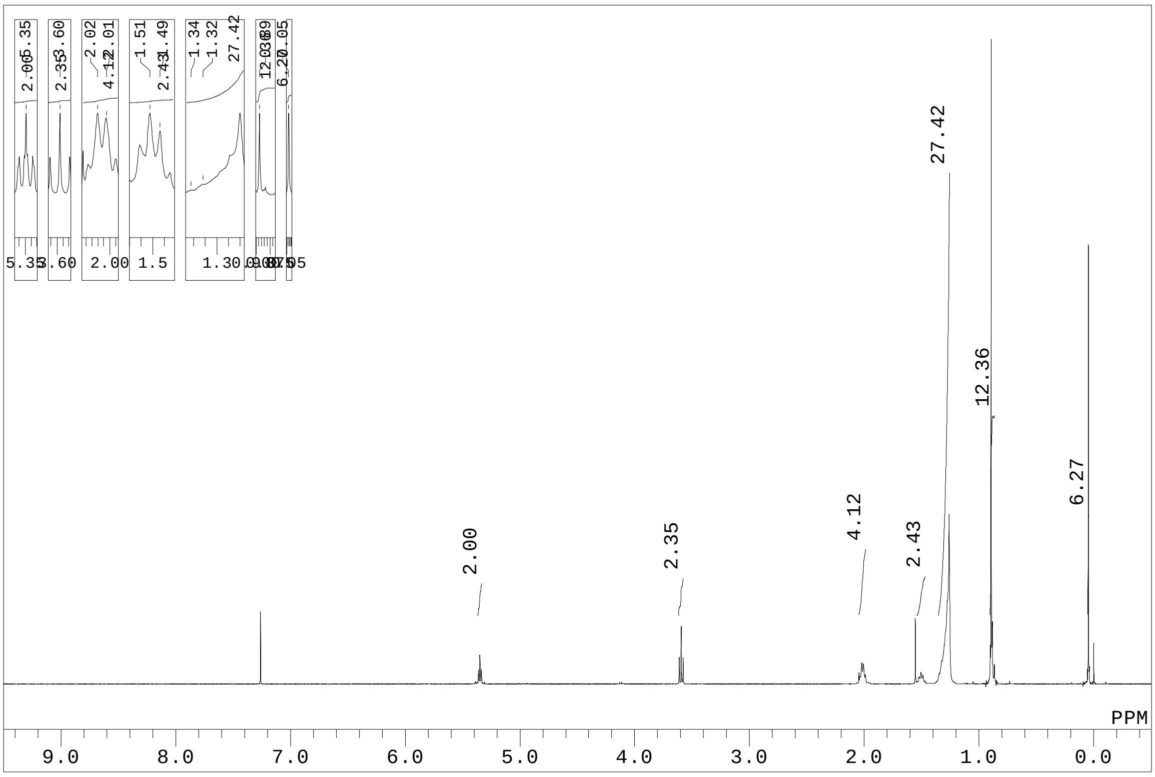

^{13}C NMR spectrum for compound S11

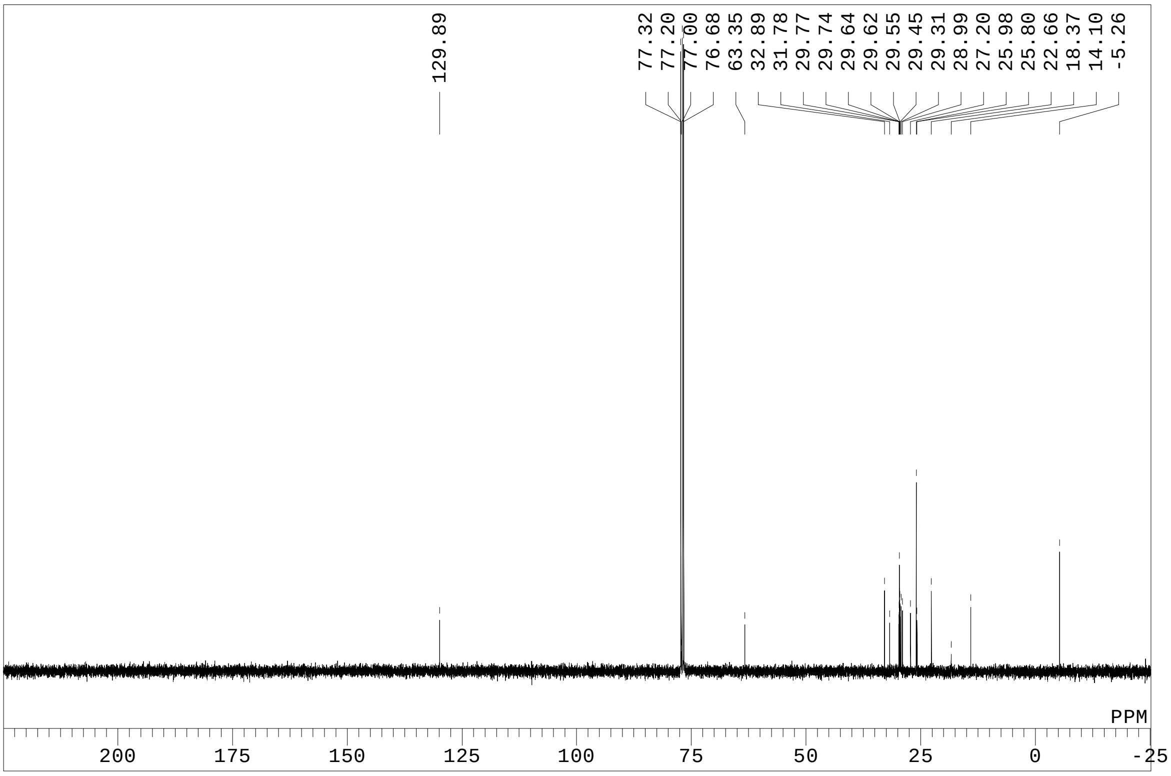

^{1}H NMR spectrum for compound 22b

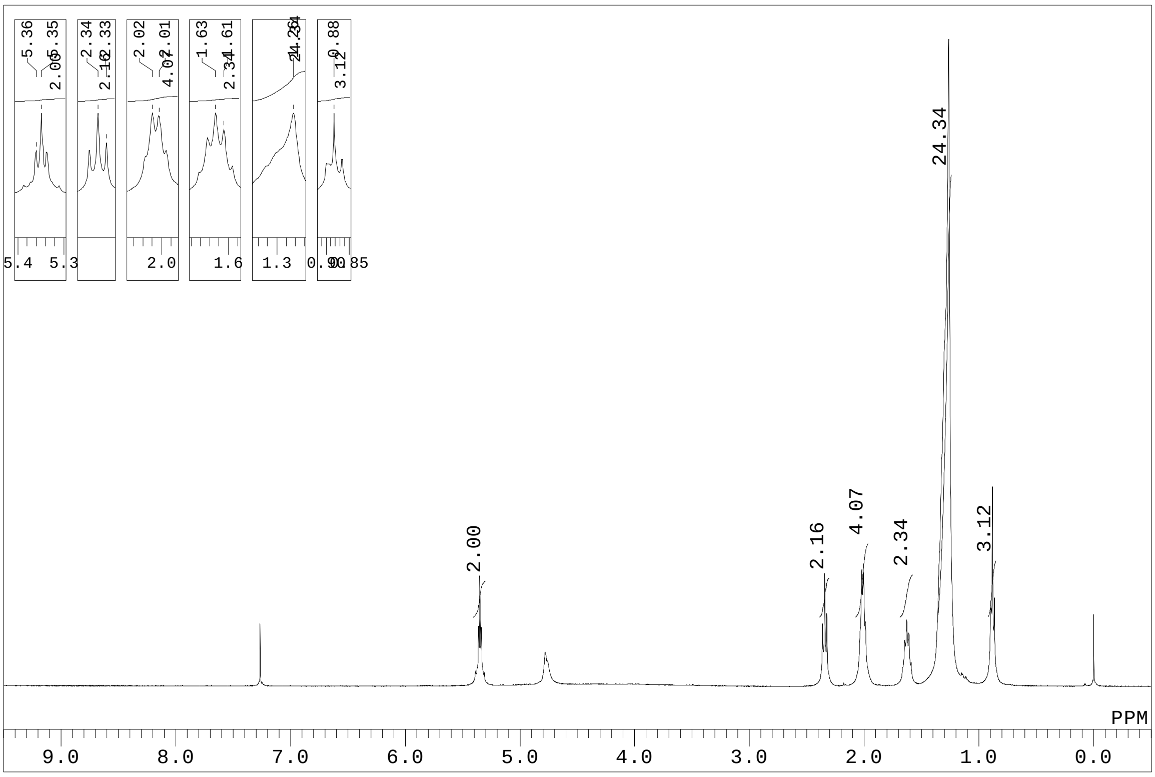

^{13}C NMR spectrum for compound 22b

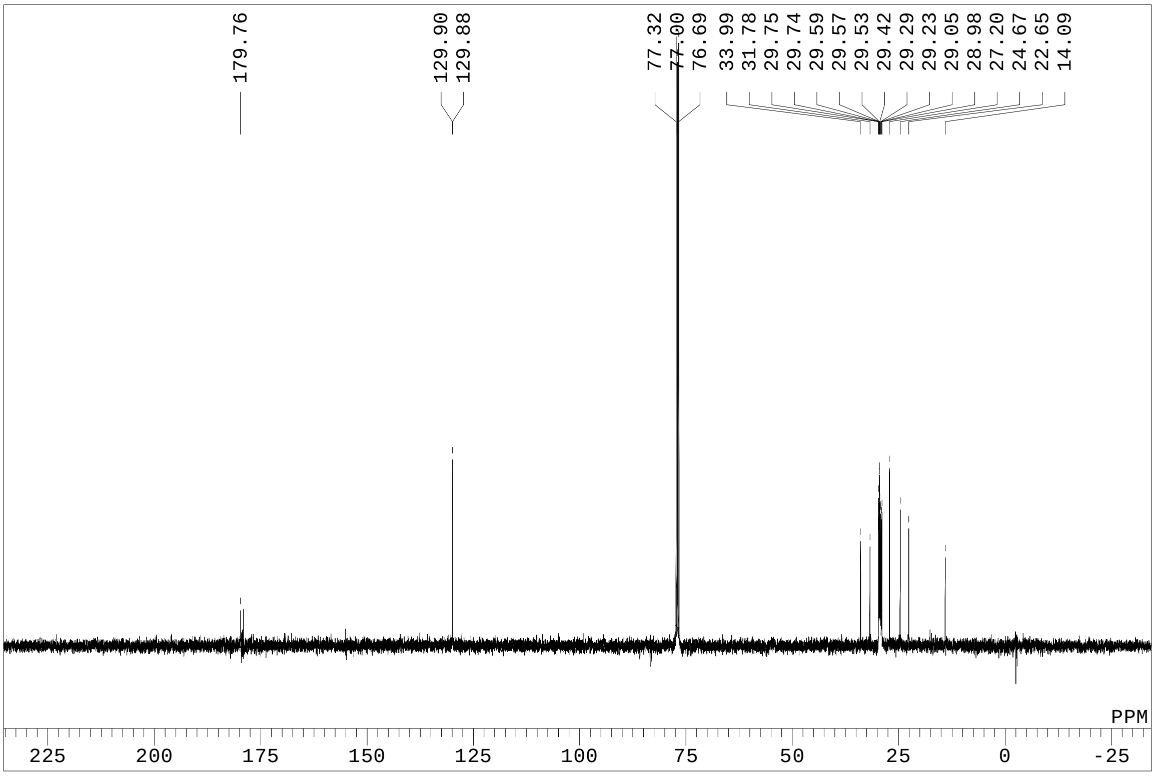

^{1}H NMR spectrum for compound 8a

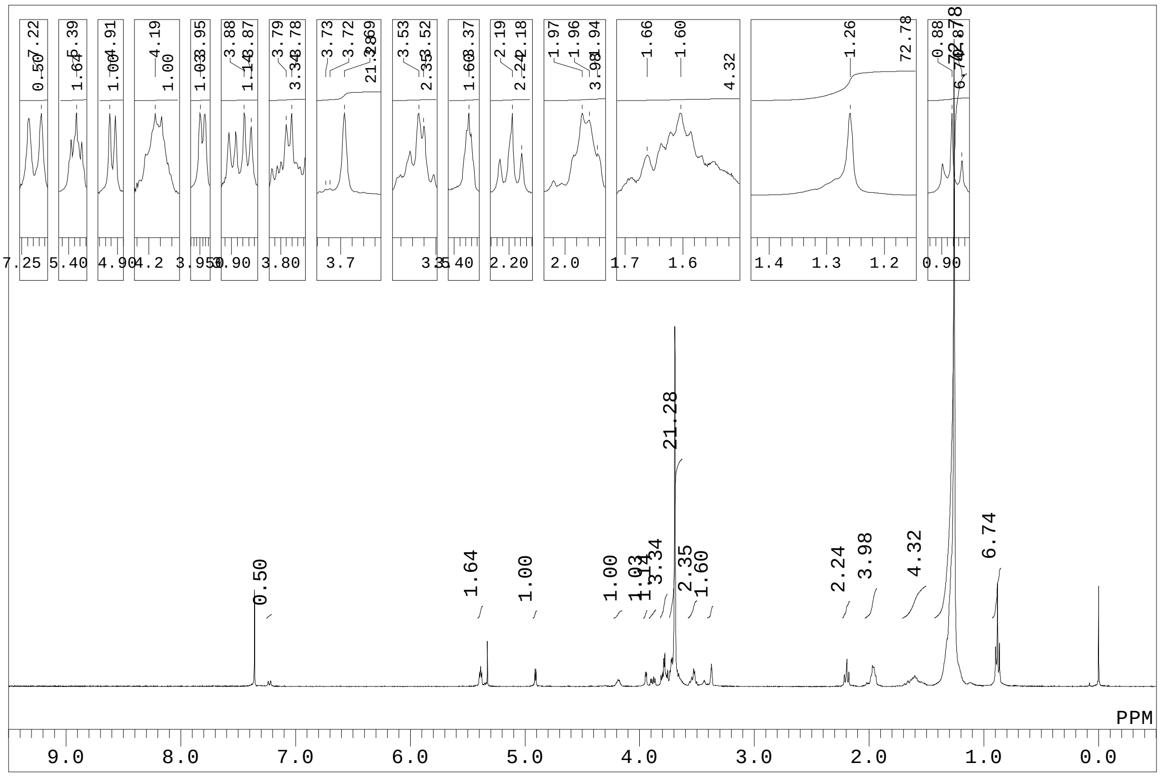

^{13}C NMR spectrum for compound 8a

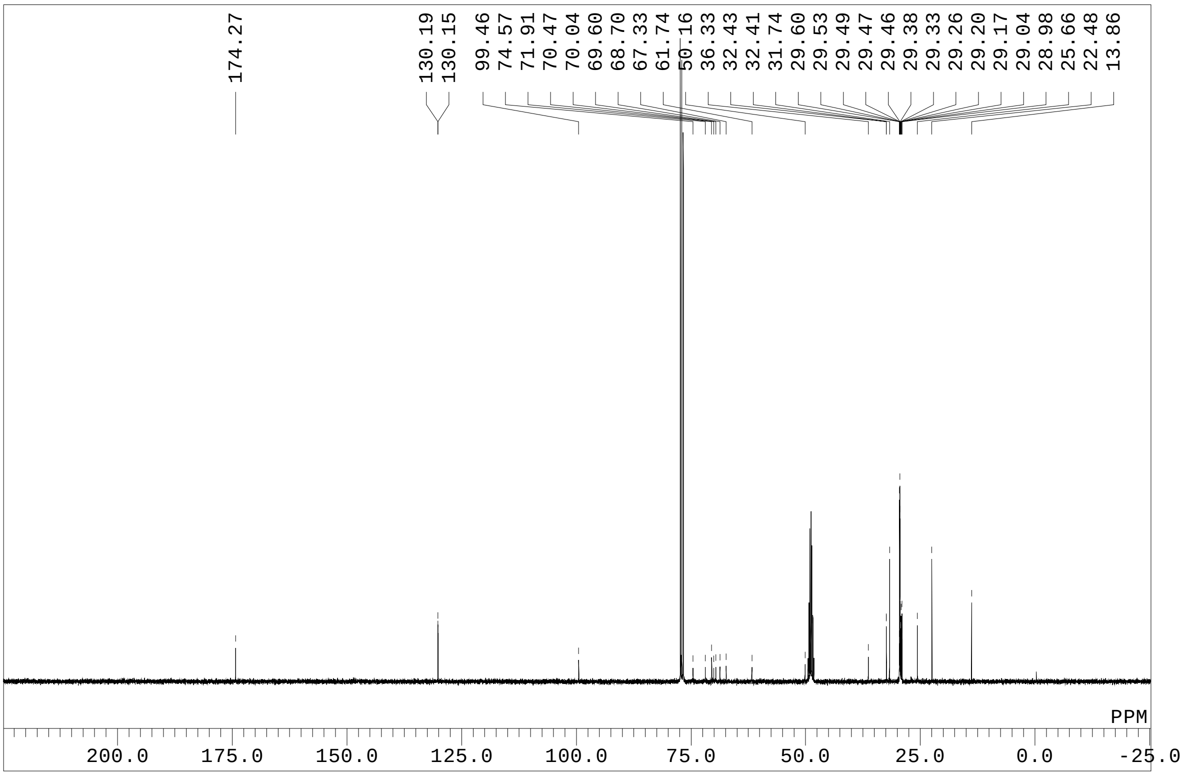

^{1}H NMR spectrum for compound 8b

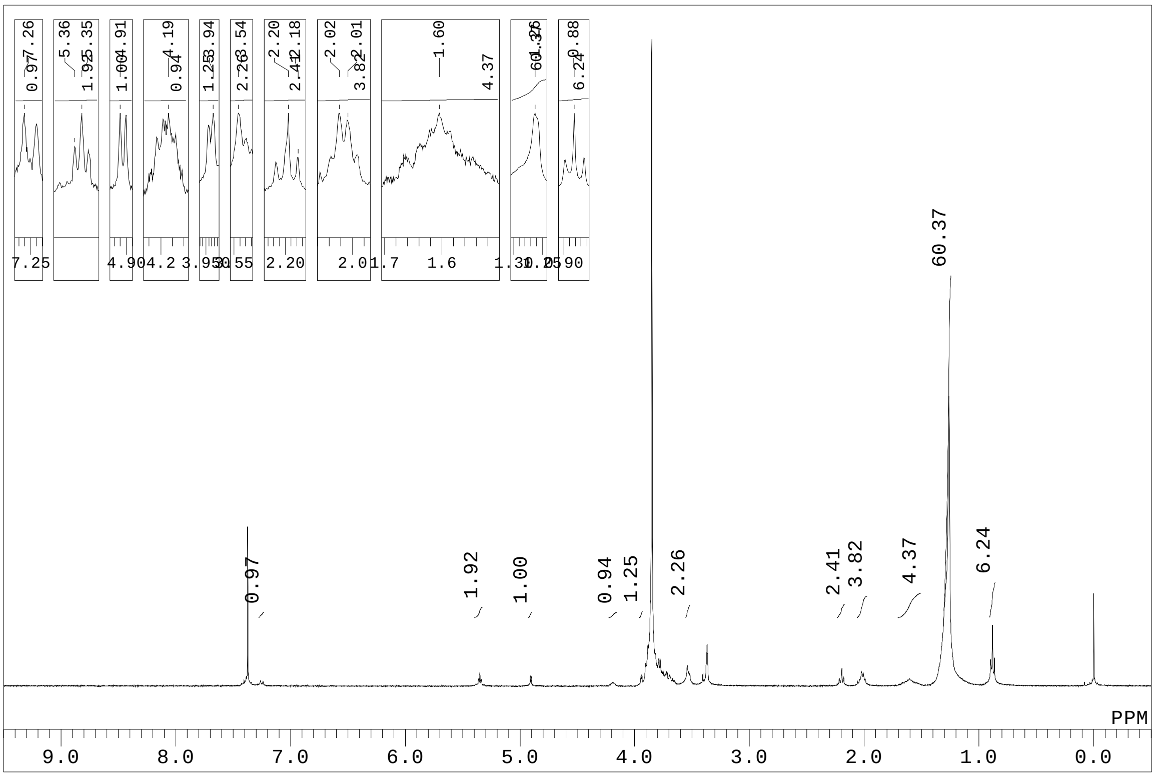

^{13}C NMR spectrum for compound 8b

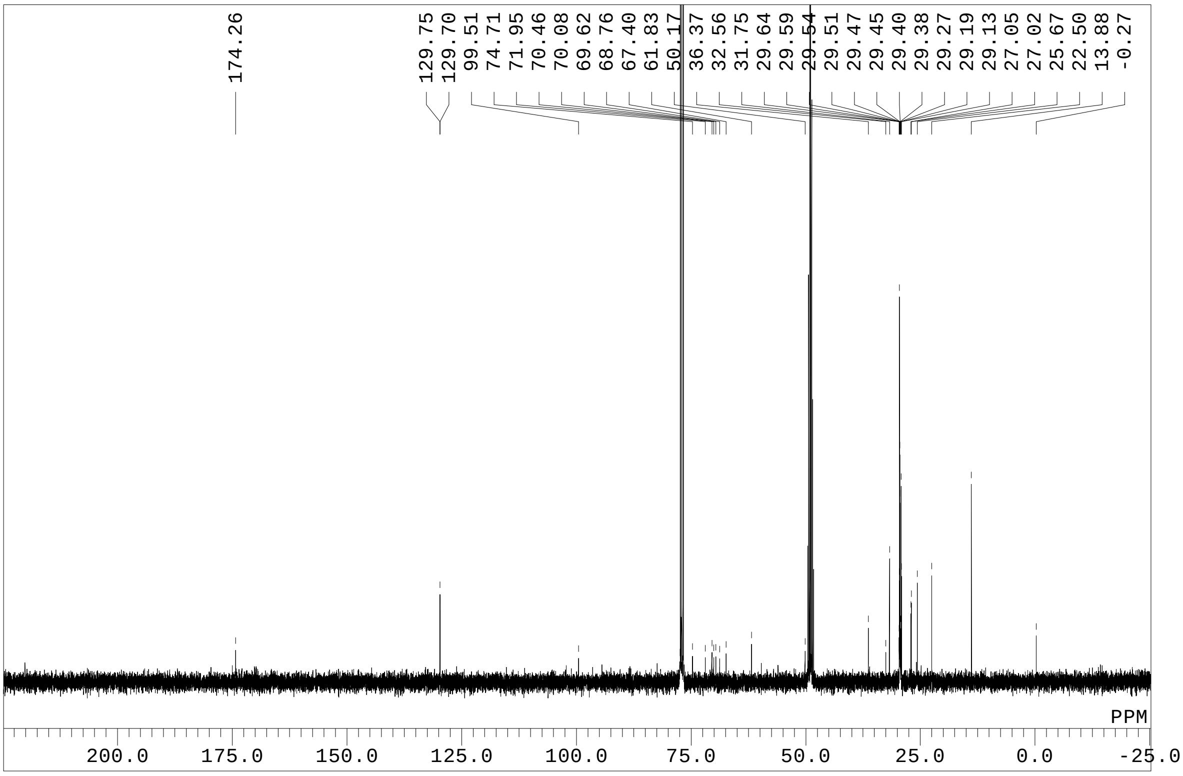

^{1}H NMR spectrum for compound 9a

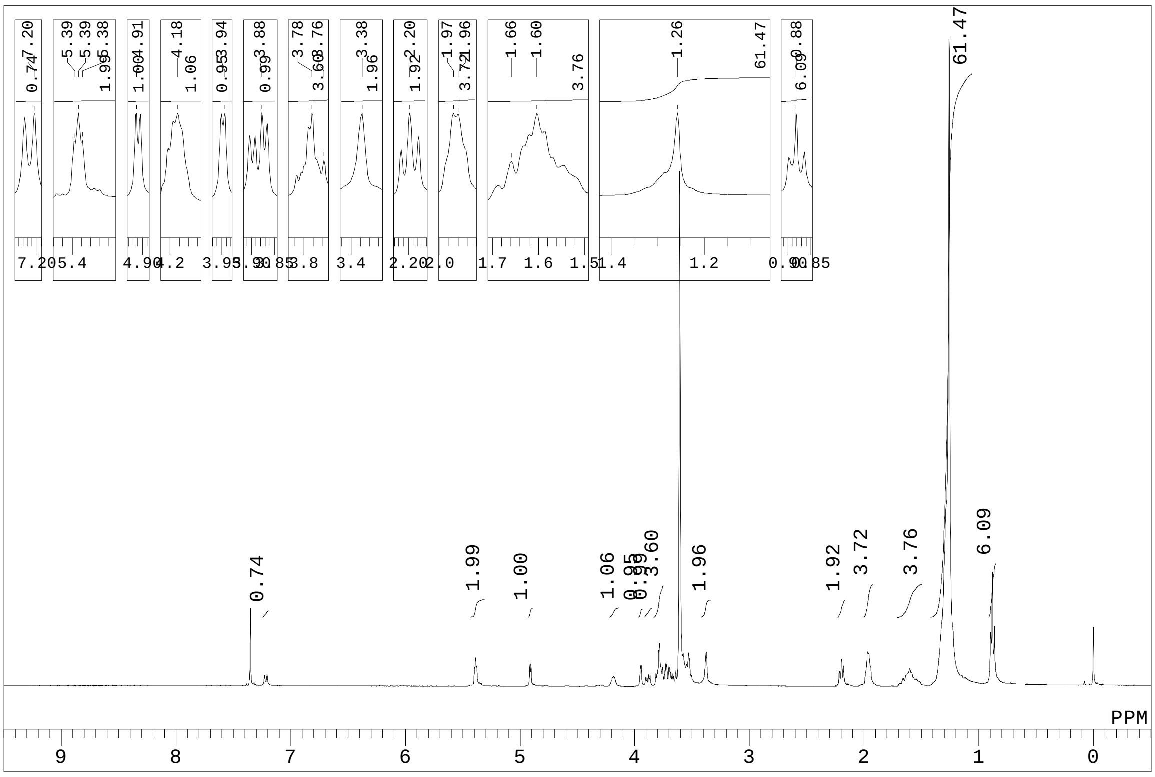

^{13}C NMR spectrum for compound 9a

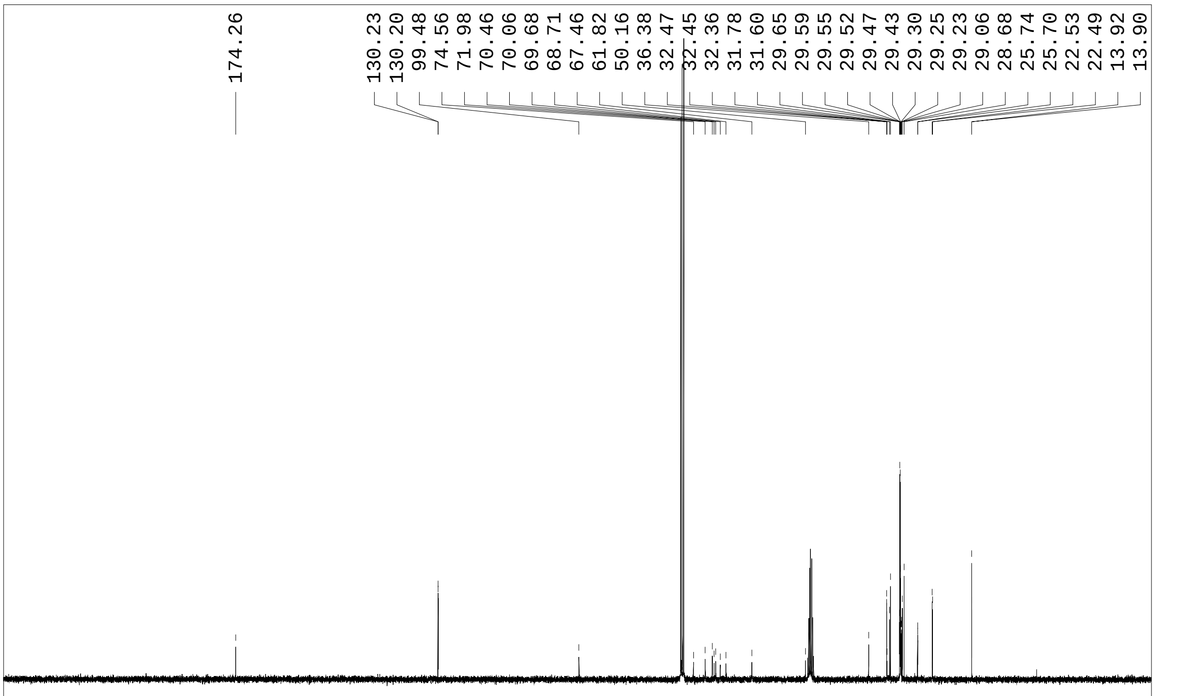

^{1}H NMR spectrum for compound 9b

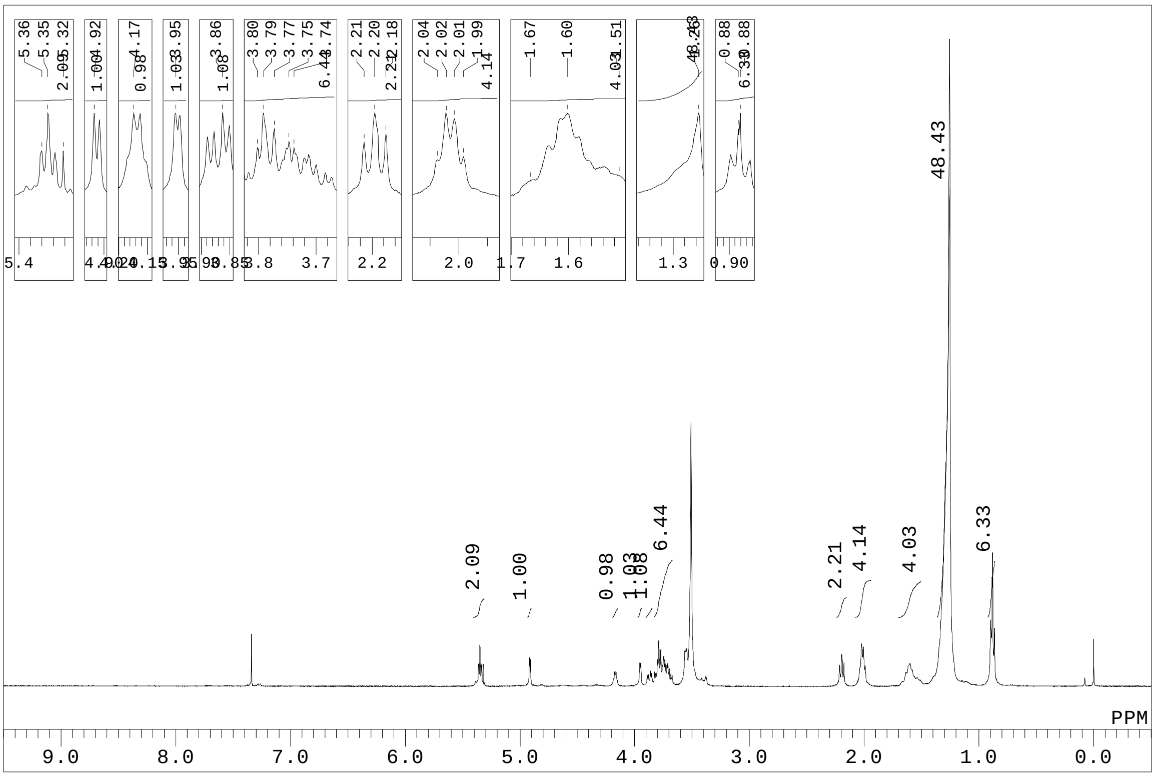

^{13}C NMR spectrum for compound 9b

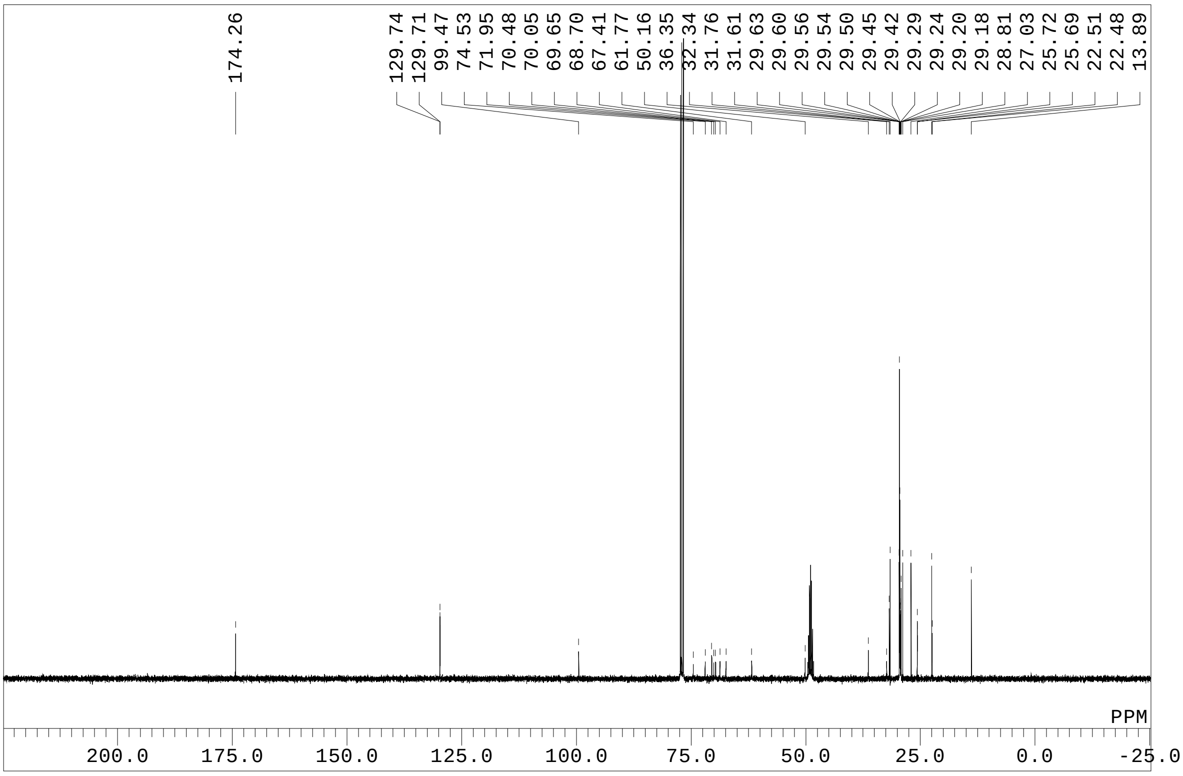

Supplement: Supplementary file 1 — Supplementary information [file 41598_2020_72280_MOESM1_ESM.pdf]
